# Structure–Optical Property Relationships in AMM′Q_3_ Chalcogenides

**DOI:** 10.1021/acs.chemmater.6c00212

**Published:** 2026-06-10

**Authors:** Ayat Tassanov, Huiju Lee, Daniel W. Spainhour, Yi Xia, James M. Hodges

**Affiliations:** † Department of Chemistry, 215862 The Pennsylvania State University, University Park, Pennsylvania 16802, United States; ‡ Department of Mechanical and Materials Engineering, 6685 Portland State University, Portland, Oregon 97201, United States

## Abstract

Ten ACuHfQ_3_ (A = Na, K, Rb, Cs; Q = S, Se, Te) chalcogenides were prepared via high-temperature solid-state methods and structurally characterized using single-crystal X-ray diffraction. The type-I AMM′Q_3_ structures are defined by the arrangement of HfQ_6_ octahedra (O) and CuQ_4_ tetrahedra (T) in their layers, which adopt either OTOT (*Cmcm*, *Pnma*) or OOTT (*Pnma*, *P*2_1_/*m*) connectivity. UV–vis absorption and photoluminescence spectra indicate optical band gaps ranging from 1.2 to 1.7 eV and reveal that OTOT and OOTT variants have direct and indirect band gaps, respectively. Density functional theory calculations corroborate these experimental observations, demonstrating that this structure–property relationship extends across the broader type-I AMM′Q_3_ family. A survey of reported compounds reveals trends linking ionic radii to structure, wherein systems with larger alkali (A^+^) cations and small chalcogen (Q^2–^) anions preferentially adopt OTOT connectivity. To formalize this trend, a machine learning model was trained on the expanded data set and used to classify compounds as either OTOT or OOTT based on ionic radii descriptors. From the model, we construct a two-dimensional structure–property map that provides a semiquantitative framework for predicting the phase and optical features in related quaternary systems. Together, these results establish a structure–property relationship in an important class of chalcogenides and demonstrate a strategy for targeting optical properties through integrated experiment and theory.

## Graphical abstract

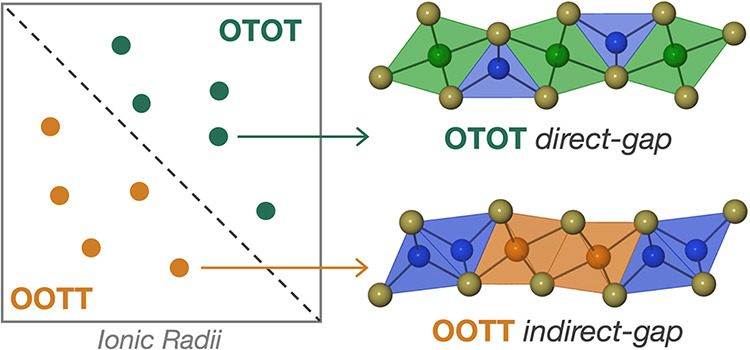

## Introduction

Metal chalcogenides are a diverse class of semiconductors with electronic features that make them well-suited for optoelectronic applications. While binary and ternary chalcogenides remain the most studied, multinary systems can have augmented functionality and provide additional levers for tuning properties.^1–9^ For example, quaternary A_2_M^II^M^IV^Q_4_ (A = alkali; Q = chalcogen) are nonlinear optical materials that mitigate harmful two-photon absorption exhibited in ternaries.^10–12^ Similarly, chemical substitution at the M^V^ site in sulvanite-derived ACu_2_M^V^Q_4_ can be used to impart Rashba spin-splitting and modulate its carrier mobility.^13,14^ Still, vast multinary phase space remains unexplored due to experimental limitations and the large number of possible elemental permutations.^16,17^

In the past decade, high-throughput computational methods have emerged as a powerful tool for assessing vast composition space.^18–22^ These studies can serve as guides for experimentalists seeking to synthesize compounds with targeted properties in a predictive manner. In a recent study, Pal et al. predicted more than 600 thermodynamically stable AMM′Q_3_ (A = s- or p-block metal; M/M′ = d- or f-block metal; Q = S, Se, Te) compounds, which belong to a large family of chalcogenides that includes more than 200 known examples.^23^ The AMM′Q_3_ quaternaries crystallize in seven different layered structure types and can be further categorized by the oxidation states of the constituent atoms, including type-I A^+^M^+^M′^4+^(Q^2–^)_3_, type-II A^2+^M^+^M′^3+^(Q^2–^)_3_, and type-III A^+^M^2+^M′^3+^(Q^2–^)_3_ variants.^24^ Their rich compositional and structural diversity provides an array of chemical tools for tuning their transport properties, which makes them candidates for thermoelectric and photovoltaic applications, among others.^25–27^

More recently, several type-I ACuZrQ_3_ chalcogenides were reported by Laing et al. and shown to be direct-gap semiconductors with ultralow thermal conductivity.^28^ Motivated by this work and our interest in understanding the bonding disparities between Zr­(IV) and Hf­(IV) chalcogenides, we have been exploring the Hf-containing analogues.

Here, we report the structure and optical properties of 10 ACuHfQ_3_ (A = Na, K, Rb, Cs; Q = S, Se, Te) compounds. The materials were synthesized using high-temperature solid-state methods and structurally characterized with single-crystal X-ray diffraction (SCXRD). The ACuHfQ_3_ products adopt three of the four known phases from the type-I subfamily and have structures composed of [CuHfQ_3_]^−^ layers separated by A^+^ countercations. We categorize the structures according to the connectivity of the HfQ_6_ octahedra (O) and CuQ_4_ tetrahedra (T) building blocks found in their [CuHfQ_3_]^−^ layers, which can be arranged in either OTOT or OOTT repeating sequences. UV–vis spectra indicate compounds with structures containing OTOT layers (*Cmcm*) have direct band gaps, while those with OOTT layers (*P*2_1_/*m* and *Pnma*) exhibit spectroscopic features that are consistent with indirect-gap semiconductors. The corresponding emission spectra, along with band structures calculated using density functional theory (DFT), provide corroborating evidence for the optical structure–property relationship, which extends to the ACuZrQ_3_ analogues.

An assessment of the type-I compounds reported in the structural databases reveals a correlation between the ionic radii of the constituent atoms and polyhedral connectivity in the AMM′Q_3_ structure. To better formalize this empirical observation, we use the expanded type-I data set to train a machine learning (ML) model that categorizes the phases as either OTOT or OOTT according to the ionic radii of the quaternary compounds. ML approaches have emerged in recent years as a platform for utilizing large data sets in materials discovery research.^29–33^ The model is used to construct a two-dimensional (2D) structure–property map and define a semiquantitative tolerance factor for predicting the phase and optical properties of arbitrary type-I quaternary chalcogenides. Phase-property diagrams offer experimentalists an intuitive guide for targeting materials, and we anticipate they will play an increasingly important role as theory and experiment continue to become more integrated.

## Results

### Synthesis and Structural Characterization

Crystals of the ACuHfQ_3_ compounds were first grown in an alkali chloride (ACl) flux and structurally characterized using single-crystal X-ray diffraction (SCXRD). In brief, stoichiometric amounts of elemental precursors were charged into fused silica tubes along with the appropriate ACl flux in a 1:2 reactant-to-flux ratio. For the Rb- and Cs-containing examples, presynthesized A_2_Q binary chalcogenide precursors were used (see the Experimental Section). The tubes were flame-sealed under dynamic vacuum, heated to 1173 K in a programmable furnace, and then soaked for 24 h. The reactions were then slow-cooled (10 K/h) to 873 K, followed by radiative cooling to room temperature, yielding black, needle-like crystals (Figure S1). Crystals were physically separated from the flux and found to be suitable for SCXRD characterization. Bulk powders were prepared using a self-flux approach, where stoichiometric amounts of precursors were reacted in flame-sealed fused silica tubes at 1273 K for 24 h. Upon radiative cooling, the 1 g ingots were pulverized into fine powders using a mortar and pestle for further characterization. Full details regarding the synthesis of the ACuHfQ_3_ compounds and A_2_Q binary precursors can be found in the Experimental Section.

Structural data for the ten ACuHfQ_3_ compounds are summarized in Table 1, and full refinement details and crystallographic tables are located in the Supporting Information. Four of the quaternaries adopt the orthorhombic *Cmcm* space group and are isostructural with KCuZrS_3_,^34^ which is the most common phase found in the AMM′Q_3_ family. Figure 1A shows the *Cmcm* structure viewed along the *a*-axis, displaying anionic [CuHfQ_3_]^−^ layers separated by counterbalancing A^+^ cations. In this structure, the covalent slabs have HfQ_6_ octahedra and CuQ_4_ tetrahedra arranged in a repeating OTOT sequence. For comparison, Figure 1B shows the related Eu_2_CuS_3_-type structure viewed along the same direction, which also has layers displaying the characteristic OTOT connectivity.^35^ The other two phases adopted by type-I AMM′Q_3_ quaternaries are shown in Figure 1C,D, both of which have the alternate OOTT polyhedral sequence in their [CuHfQ_3_] layers. Of the 10 Hf-containing examples, only the NaCuHfS_3_ quaternary adopts the *Pnma* structure shown in Figure 1C (NaCuTiS_3_-type).^36^ Here, the polyhedra in the layers are tilted in alternating directions when moving along the *b*-axis. Five of the compounds crystallize in the monoclinic *P*2_1_/*m* space group, including all of the tellurides, which is displayed in Figure 1D. The monoclinic phase has similar layers as the *Pnma* structure but with the polyhedra tilted in the same direction for each layer. We note that the TlCuTiTe_3_ structural prototype is the least common in the family.^37^

**Table 1:** Crystallographic Data for ACuHfQ_3_ Compounds^a^

empirical formula	NaCuHfS_3_	RbCuHfS_3_	CsCuHfS_3_	KCuHfSe_3_	RbCuHfSe_3_	CsCuHfSe_3_
space group	*Pnma*	*Cmcm*	*Cmcm*	*P*2_1_/*m*	*Cmcm*	*Cmcm*
*a* (Å)	12.8840(2)	3.7261(2)	3.7443(2)	8.5861(4)	3.8665(3)	3.88180(10)
*b* (Å)	3.6908(1)	14.5594(8)	15.2863(9)	3.8755(1)	15.0787(1)	15.8371(4)
*c* (Å)	9.8722(2)	9.7569(5)	9.7795(5)	9.4339(4)	10.1499(7)	10.1703(3)
*V* (Å^3^)	469.44(2)	529.31(5)	559.74(5)	293.64(2)	591.76(8)	625.23(3)
β (deg)	90	90	90	110.706(5)	90	90
ρ (g/cm^3^)	5.111	5.317	5.591	5.859	6.335	6.500
μ (Cu Kα)/mm^–1^	57.532	61.348	97.459	62.914	66.088	97.864
*R*_int_	0.0497	0.0340	0.0382	0.0242	0.0252	0.0263
*R*_1_, w*R*_2_ [*I* > 2σ(*I*)]	0.0538, 0.1544	0.0472, 0.1200	0.0264, 0.0750	0.0446, 0.1257	0.0379, 0.1062	0.0404, 0.1082
*R*_1_, w*R*_2_ [all data]	0.0550, 0.1561	0.0494, 0.1228	0.0268, 0.0752	0.0448, 0.1258	0.0381, 0.1068	0.0410, 0.1089
Δρ_min_, Δρ_max_ (e/Å^3^)	3.13, −2.17	2.74, −1.88	1.25, −2.01	2.63, −2.59	1.91, −1.68	2.39, −1.89

empirical formula	NaCuHfTe_3_	KCuHfTe_3_	RbCuHfTe_3_	CsCuHfTe_3_
space group	*P*2_1_/*m*	*P*2_1_/*m*	*P*2_1_/*m*	*P*2_1_/*m*
*a* (Å)	8.2105(1)	8.5914(3)	9.4034(7)	10.8326(8)
*b* (Å)	4.0344(5)	4.1129(2)	4.1366(3)	3.9617(3)
*c* (Å)	10.2118(1)	10.6995(5)	10.3921(8)	10.8989(8)
*V* (Å^3^)	321.12(7)	351.39(3)	371.91(5)	406.43(6)
β (deg)	108.317(1)	111.654(5)	113.068(9)	119.665(1)
ρ (g/cm^3^)	6.700	6.275	6.343	6.192
μ (Cu Kα)/mm^–1^	138.334	131.119	126.789	143.184
*R*_int_	0.0898	0.0445	0.0499	0.0711
*R*_1_, w*R*_2_ [*I* > 2σ(*I*)]	0.0693, 0.1804	0.0474, 0.1259	0.0586, 0.1518	0.0577, 0.1415
*R*_1_, w*R*_2_ [all data]	0.0780, 0.1899	0.0481, 0.1271	0.0644, 0.1562	0.0757, 0.1508
Δρ_min_, Δρ_max_ (e/Å^3^)	3.03, −2.83	2.63, −3.02	2.63, −2.54	3.25, −3.65

a Full details regarding data collection and structural refinements can be found in the Supporting Information.

**Figure 1:**
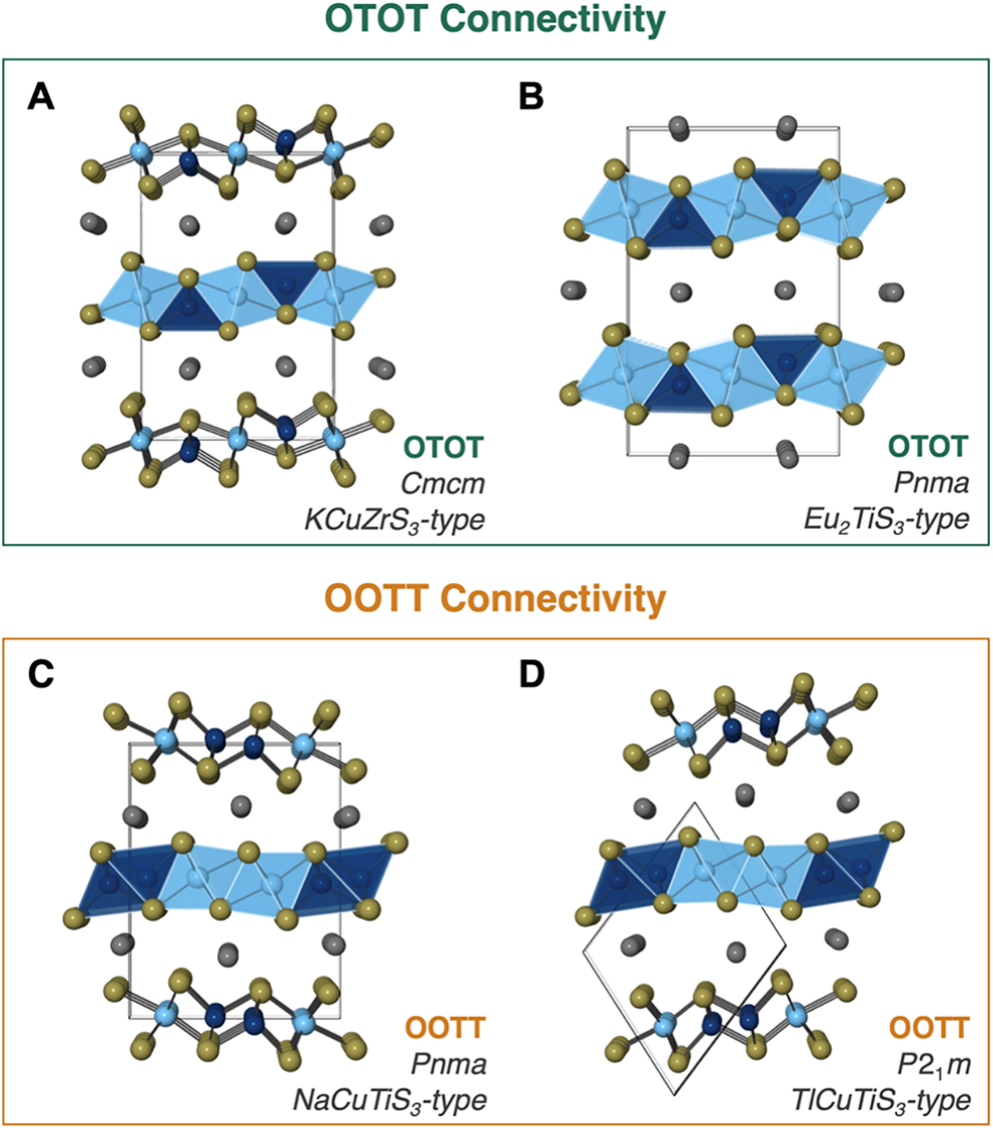
Crystal structures of Type-I AMM′Q_3_ compounds grouped by connectivity of octahedral (O) and tetrahedral (T) building blocks. (A, B) OTOT and (C, D) OOTT arrangements are shown. Notably, structures (B) and (C) both adopt *Pnma* symmetry but differ in connectivity.

Bulk powders of the title compounds were structurally characterized using powder X-ray diffraction (pXRD), and the diffractograms for the sulfides are shown in Figure 2A–D. The previously reported KCuHfS_3_ material was also prepared and characterized for comparison. In each case, the experimental patterns are in good agreement with the simulated diffractograms, all expected reflections are observed, and no significant impurity phases are detected. Differences in relative peak intensities between experimental and simulated patterns are attributed to preferred orientation, which is commonly observed in layered chalcogenides. In particular, reflections corresponding to planes parallel to the [CuHfQ_3_]^-^ slabs, such as the (200) and (400) peaks, are systematically enhanced, a phenomena that has been observed in other AMM′Q_3_ systems.^38,39^ These reflections are indexed for clarity. Figure S2A–D shows the diffractograms for the ACuHfSe_3_ series, which are consistent with the simulated patterns but exhibit a greater degree of preferred orientation, particularly in compounds that adopt the *P*2_1_/*m* phase. Attempts to prepare bulk powders of the ACuHfTe_3_ tellurides were unsuccessful, yielding complex mixtures of phases that could not be reliably indexed. Accordingly, the optical properties of the tellurides are not included in this report.

**Figure 2:**
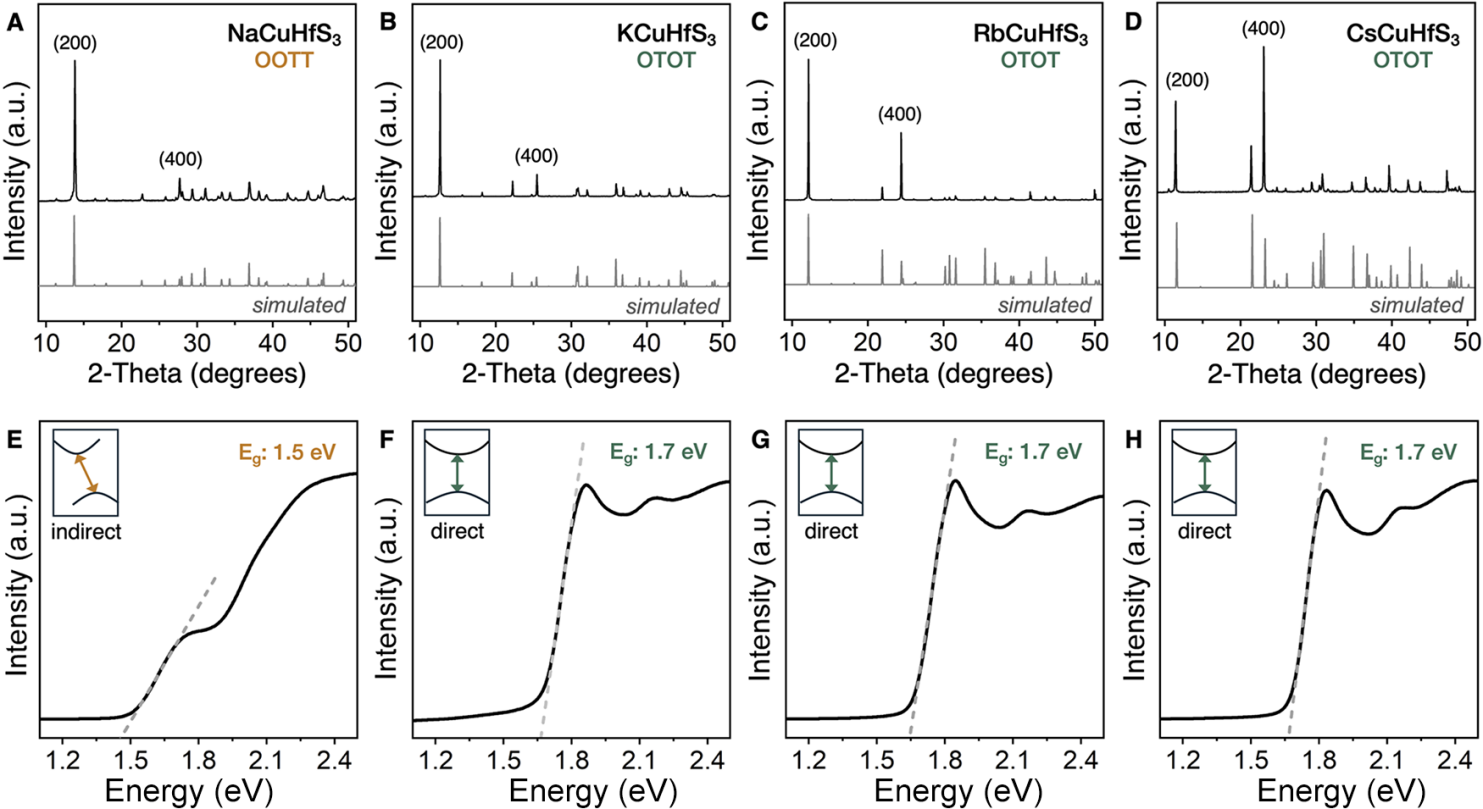
Powder X-ray diffraction and absorption spectra for ACuHfS_3_ sulfides. Experimental diffractograms for (A) NaCuHfS_3_, (B) KCuHfS_3_, (C) RbCuHfS_3_, and (D) CsCuHfS_3_ are consistent with simulated patterns (gray). (E–H) Absorption spectra show a gradual onset for NaCuHfS_3_ (indirect, *E*_g_ ≈ 1.5 eV) and sharper onsets for KCuHfS_3_, RbCuHfS_3_, and CsCuHfS_3_ (direct, *E*_g_ ≈ 1.7 eV).

### UV–vis and Photoluminescence (PL) Spectroscopy

The absorption spectra of the ACuHfQ_3_ sulfides and selenides were collected with a UV–vis spectrophotometer operating in diffuse-reflectance mode. The data were converted to Kubelka–Munk (KM) absorption plots, and the band gaps (*E*_g_) of the crystalline solids were estimated by extrapolating the linear region of the absorption curves to the energy axis. We note that although Tauc plots can improve the accuracy of *E*_g_ measurements in many systems, the analysis is not valid unless several criteria are met.^40^ In such cases, absorption spectra can be used to identify trends and make qualitative assessments regarding electronic transitions. Previous AMM′Q_3_ studies have employed such methods,^28^ and accordingly, we use the absorption spectra here to estimate the *E*_g_ for each of the samples.

Figure 2E shows the absorption spectra for NaCuHfS_3_, which has the *Pnma* structure with OOTT layers (NaCuTiS_3_-type). The spectra show an onset energy at 1.5 eV and a linear region with a gradual slope when compared to the other Hf-containing sulfides (Figure 2F–H). The ACuHfS_3_ (A = K, Rb, Cs) compounds adopt the *Cmcm* phase with OTOT connectivity in their layers and exhibit spectra with sharp onset energies at 1.7 eV and nearly identical secondary absorption features. On their own, the absorption data did not conclusively confirm the presence of direct or indirect band gaps prior to examining the band structures (see below). Still, it is clear that the absorption spectra for NaCuHfS_3_ are qualitatively different from the other sulfides, the latter having comparatively sharp onset energies more consistent with direct-gap materials. The data also indicate that the alkali metal does not influence the band gap of materials with the same structure type.

The absorption data for the ACuHfSe_3_ selenides are shown in Figure S2E–H. Here, the Na- and K-containing products adopt the *P*2_1_/*m* (OOTT) structure, while the Rb and Cs variants have the *Cmcm* (OTOT) phase. Spectra for the compounds with OOTT and OTOT layers exhibit different features, with the latter having linear regions with steeper slopes. Again, the data indicate that the size of the alkali cations does not influence the magnitude of the band gap, with NaCuHfSe_3_ and KCuHfSe_3_ having *E*_g_ values of 1.3 eV, and RbCuHfSe_3_ and CsCuHfSe_3_ are both estimated to be 1.2 eV.

Photoluminescence (PL) spectroscopy was used to further characterize the ACuHfS_3_ (A = Na, Rb, Cs) sulfides. Figure 3A and 3B show the emission spectra along with absorption profiles for NaCuHfS_3_ (*Pnma*, OOTT) and CsCuHfS_3_ (*Cmcm*, OTOT), respectively. Qualitatively, the spectra for NaCuHfS_3_ (OOTT) are clearly distinct from those of CsCuHfS_3_ (OTOT), showing a broad PL peak at 1.4 eV and a sharp PL peak at 1.7 eV, respectively. Again, the optoelectronic features of the OOTT phase are consistent with an indirect-gap semiconductor, showing weaker intensity, larger Stokes shift, and broader peaks than those of the OTOT phase. We note that the low-energy limit of the detector for the PL spectrometer was 1.3 eV, and for this reason, interpretable emission could not be collected for the quaternary selenides.

**Figure 3:**
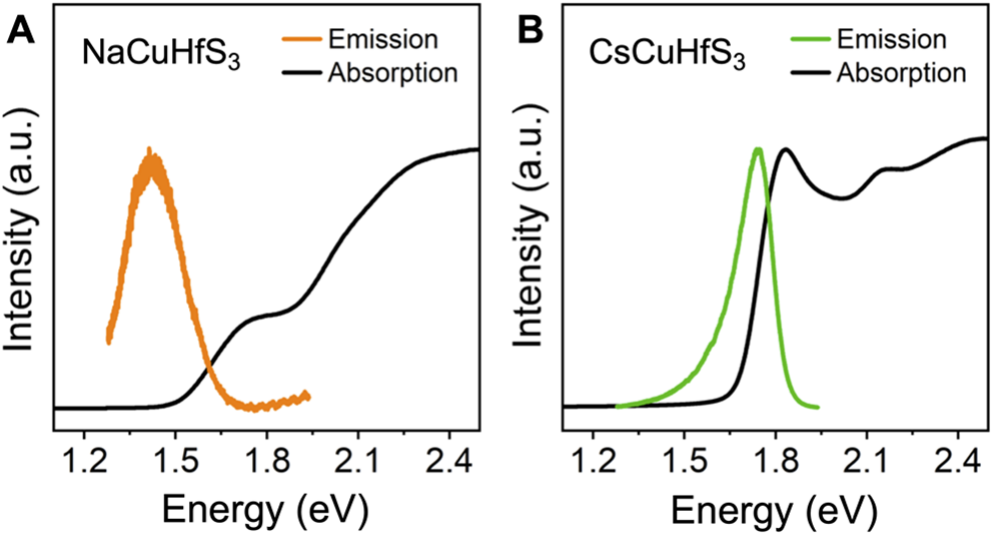
Photoluminescence spectra of (A) NaCuHfS_3_ and (B) CsCuHfS_3_. Emission is shown in color and absorption in gray. The OOTT phase exhibits a larger Stokes shift than the OTOT phases.

To determine if the optoelectronic trends extend to other members of the type-I subclass, the Zr-containing ACuZrQ_3_ (A = Na, K; Q = S, Se) congeners were prepared, and the absorption spectra are shown in Figure S3. Here, the spectra for NaCuZrS_3_ (OTOT) exhibit a sharper onset energy and steeper linear region of the absorption curve when compared to NaCuZrSe_3_ (OOTT), with estimated *E*_g_ values of 1.4 and 1.0 eV, respectively. The absorption spectra for KCuZrS_3_ (OTOT) and KCuZrSe_3_ (OTOT) are also consistent with the trends observed in the Hf series, both exhibiting absorption curves with steep linear regions and *E*_g_ values estimated to be 1.4 and 0.9 eV, respectively. Again, the alkali metal does not have a strong influence on the *E*_g_, although the Zr-containing quaternaries have systematically lower E_g_ values (see Discussion).

### Band Structure Calculations

Band structure diagrams for the 10 ACuHfQ_3_ compounds were calculated using density functional theory (DFT)^41,42^ and can be found in Figure S4 along with corresponding partial density of states (pDOS). The diagrams for CsCuHfS_3_ (*Cmcm*, OTOT) and KCuHfSe_3_ (*P*2_1_/*m*, OOTT) are shown in Figure 4 with a narrowed energy range in order to compare the electronic features of the OTOT and OOTT phases. For both examples, the valence band maxima (VBM) are composed of both Cu and S/Se orbitals, while contributions to the conduction band minima (CBM) are primarily from Hf orbitals. We note that it is common for Cu d and S/Se p orbitals to contribute to the DOS at the VBM in metal chalcogenides.^43–45^ The band structures reveal CsCuHfS_3_ has a direct transition (Γ–Γ) calculated to be 0.78 eV, while KCuHfSe_3_ has an indirect gap (Γ–Y) of 0.42 eV. The calculated *E*_g_ values are systematically lower than those estimated from absorption spectra due to underestimation of band gaps in DFT calculations when using the Perdew–Burke–Ernzerhof (PBE) exchange–correlation functional.^46^ Nevertheless, the calculations corroborate the experimental data, showing that the OTOT structures are expected to have direct band gaps while the OOTT structures are indirect-gap semiconductors.

**Figure 4:**
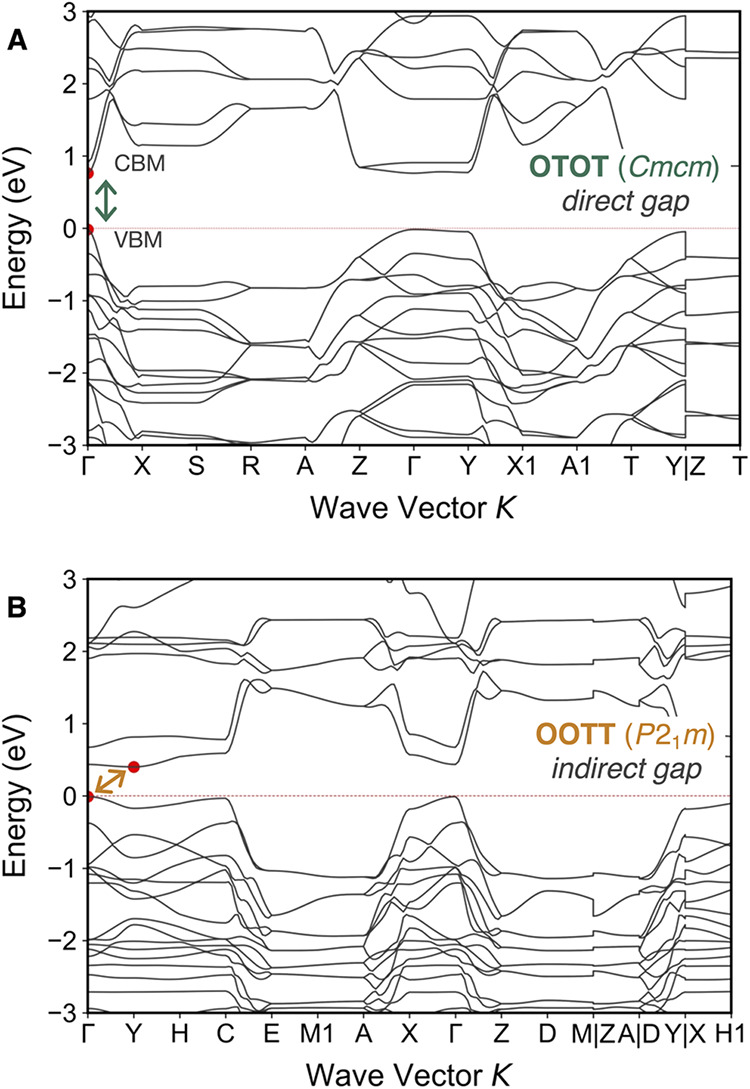
DFT-calculated band structures of representative ACuHfQ_3_ phases showing (A) a direct band gap for the OTOT (*Cmcm*) phase and (B) an indirect band gap for the OOTT (*P*2_1_/*m*) phase.

## Discussion

The spectroscopic data for the ACuHfQ_3_ chalcogenides, together with DFT-calculated band structures, reveal a clear structure–property relationship in type-I AMM′Q_3_ chalcogenides, in which the connectivity of the structural building blocks governs whether the materials exhibit direct or indirect band gaps. Specifically, compounds with layers composed of M'Q_6_ octahedra (O) and MQ_4_ tetrahedra (T) arranged in an OTOT sequence (*Cmcm*, *Pnma*) have direct band gaps, while those adopting the alternate OOTT connectivity (*Pnma*, *P*2_1_/*m*) exhibit indirect band gaps. A survey of the AMM′Q_3_ literature corroborates this trend.^28^ The difference in band gap character of the OTOT and OOTT structures could arise from changes in the M′–Q orbital overlap associated with the differing connectivities, since subtle changes in bonding can facilitate a change from a direct to indirect transition.^47^

Figure 5A summarizes the phase space for the ACuZrQ_3_ and ACuHfQ_3_ compositions, where the phases are categorized as either OOTT or OTOT. Notably, the Hf-containing examples show a higher prevalence for the OOTT structural variants. This is somewhat unexpected, as Zr^4+^ and Hf^4+^ are often structurally interchangeable due to their similar size and bonding characteristics.^48–50^ However, the empirically observed M′–Q bond distances in both systems are larger than expected from tabulated values used in bond valence sum analysis. For example, in CsCuHfS_3_ and CsCuZrS_3_, the average M′–S bond distances (2.575 and 2.593 Å, respectively) exceed the expected values of 2.39 and 2.41 Å. These deviations reflect the complex bonding environment in the quaternary framework, where the chalcogen anions coordinate M′, Cu, and alkali cations. Subtle differences between Zr and Hf are therefore amplified by the structural framework, resulting in distinct connectivity preferences.

**Figure 5:**
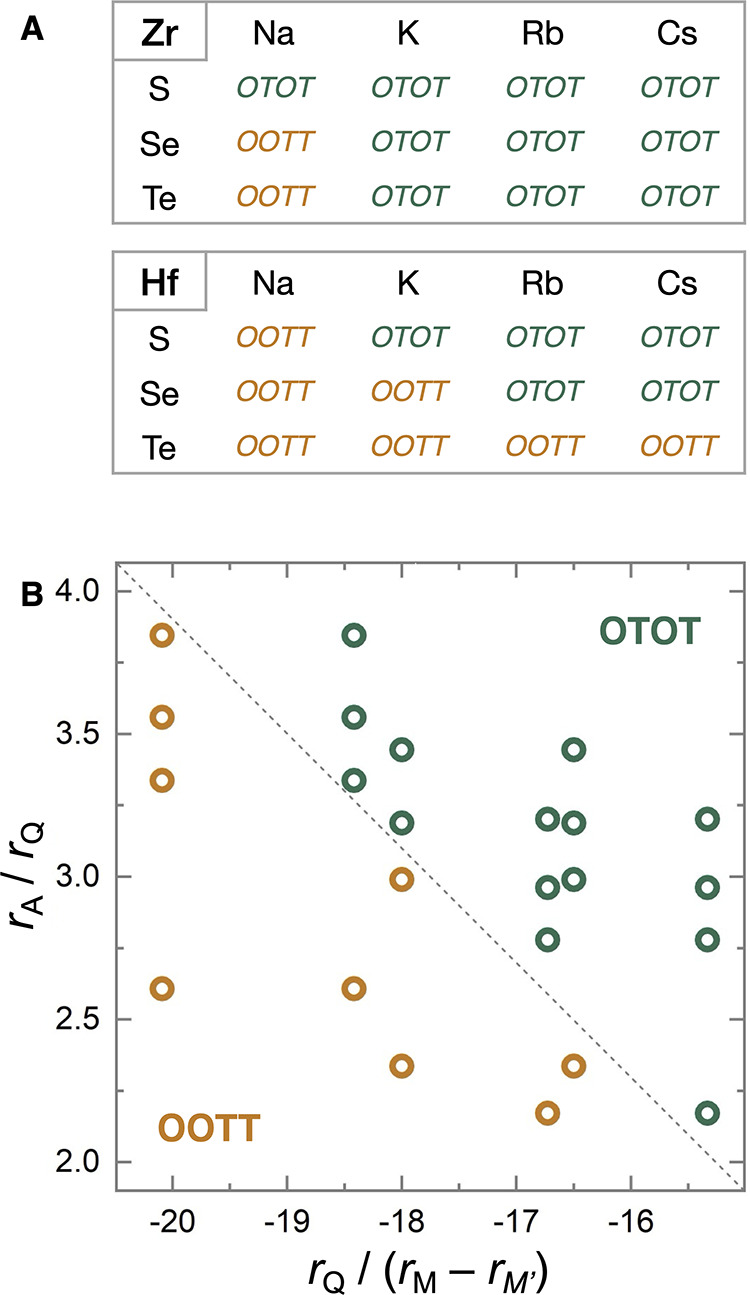
(A) Structural trends in the ACuM′Q_3_ (M′ = Zr, Hf) phase space. The OOTT phases are favored in the Hf analogues and with decreasing Q^2–^ anion and A^+^ cation size. (B) Structure–property map showing OTOT and OOTT phases as a function of ionic radii. The dashed line indicates the approximate boundary between connectivity types.

The distribution of phases further indicates that OTOT connectivity is favored with increasing A^+^ cation size and decreasing Q^2–^ size, corresponding to more ionic compositions. This is supported by differences in cation–cation interactions across the alternate polyhedral configurations. Table S1 displays the Cu–Cu distances for all of the ACuHfQ_3_ phases. In OOTT structures, the Cu–Cu distances are notably shorter, reflecting a more constrained and distorted framework that is accompanied by systematically smaller band gaps. In contrast, OTOT structures lack such short cation–cation contacts and are therefore more consistent with an ionic bonding environment.

To develop a semiquantitative framework for predicting the preferred sequence of polyhedral building blocks (either OTOT or OOTT) based on ionic radii in quaternary compounds, we constructed a machine learning model consisting of a two-dimensional descriptor using the SISSO (Sure Independence Screening and Sparsifying Operator) algorithm.^51^ We used as input the classification of polyhedral building blocks from experimentally synthesized compounds (Figure 5B). The SISSO model was trained using the ionic radii of the four constituent elements, sourced from the Mendeleev database.^52^ The SISSO algorithm achieved 100% classification accuracy using two derived descriptors: *r*(Q)/(*r*(M) – *r*(M′)) and *r*(A)/*r*(Q). As illustrated in Figure 5B, the decision boundary between the OTOT and OOTT regions effectively serves as a semiquantitative tolerance factor, further illustrating the dependence of the preferred sequence of polyhedral building blocks on the ionic radii of the constituent elements.

## Conclusion

The nature of the electronic transition in chalcogenide semiconductors is an important design parameter in optoelectronic applications. However, this can be difficult to predict when approaching systems that cover broad composition space. Here, we use the type-I AMM′Q_3_ family of chalcogenides to demonstrate how ML-derived structure–property diagrams can be used as a guide for synthesizing semiconductors with targeted electronic features. The ten ACuHfQ_3_ compounds described in this report adopt structures with different arrangements of HfQ_6_ octahedra (O) and CuQ_4_ tetrahedra (T) in their layers. Spectroscopic analysis of the semiconductors shows that the arrangement of polyhedral building blocks defines their electronic structure, where compounds having OTOT and OOTT layers are found to be direct- and indirect-gap materials, respectively. The preferred structure adopted by the quaternaries is dictated by the constituent ionic radii. By training a machine learning model using the broader type-I data, we construct a structure–property map based on simple ionic radii, which can be used to target quaternary chalcogenides with desired electronic attributes. We anticipate the approach will readily extend to other large families of multinary compounds, providing a tool that can accelerate exploratory research.

## Materials and Methods

### Materials

The following materials were used as received: copper chunks (Cu, 99.999%), sulfur chunks (S, 99.999%), selenium shot (Se, 99.999%), and tellurium chunks (Te, 99.999%) were purchased from American Elements. Sodium chunks (Na, 98%), potassium chunks (K, 98%), and cesium metal (Cs, 99.8%) were purchased from Fisher Scientific. The following materials were dried in a vacuum oven set to 200 °C for 24 h and then stored in a nitrogen-filled glovebox: sodium chloride (NaCl, 99%), potassium chloride (KCl, 99%), rubidium chloride (RbCl, 99%), and cesium chloride (CsCl, 99%) were purchased from Sigma-Aldrich.

### Synthesis of Binary Alkali Chalcogenides

A_2_Q (A = Rb, Cs; Q = S, Se, Te) binary precursors were synthesized by reacting Cs or Rb metal with the respective chalcogen in liquid ammonia, as described previously.^53^ Approximately 20 g of the targeted binary chalcogenide was prepared in each reaction. In a typical reaction, Cs or Rb metal was gently heated in a nitrogen-filled glovebox, and then pipetted into a 250 mL three-neck round-bottom flask equipped with a borosilicate stir bar. A glass stopcock equipped with a Teflon valve was placed at the center port of the flask and the side ports were capped with glass stoppers. The airtight flask was then transferred and connected to a Schlenk line that was purged with nitrogen. Approximately 150 mL of ammonia was condensed into the flask using a dry ice/acetone bath (−77 °C), which dissolved the alkali metal to form a dark blue solution. Finely ground chalcogen was carefully added under an increased flow of nitrogen and the mixture was stirred for several hours. 5% excess of the stoichiometric amount of chalcogen was used to minimize presence of unreacted alkali metal in the final product. Upon completion of the reaction, the solution above the off-white precipitate became clear. The dry ice/acetone bath was removed to allow the ammonia to evaporate, and the product was then dried under vacuum overnight before being stored in a nitrogen-filled glovebox for future use.

### Flux Synthesis of ACuHfQ_3_ Crystals

Crystals of the titled compounds were first grown in the appropriate alkali halide flux. Approximately 1 g of the targeted ACuHfQ_3_ quaternary was prepared in each reaction. Carbon-coated tubes were charged with stoichiometric amounts of elemental precursors (1 g) and covered with 2 g of the appropriate alkali halide flux, then flame-sealed under dynamic vacuum (10^–3^ Torr). The reaction vessels were then placed in a programmable furnace and heated to 450 °C at 100 °C/h, held at 450 °C for 5 h, and then heated to 1000 °C at a rate of 100 °C/h. After dwelling at 1000 °C for 24 h, the vessels were cooled to 600 °C at a rate of 10 °C/h, followed by radiative cooling to room temperature. Needle-like crystals were separated from the flux by hand and found to be suitable for structural characterization via single-crystal X-ray diffraction.

### Direct Synthesis of ACuHfQ_3_ Compounds

Ingots of the titled sulfides and selenides were synthesized by reacting stoichiometric amounts of elemental or binary (Rb_2_Q, Cs_2_Q) precursors. Approximately 1 g of the targeted ACuHfQ_3_ quaternary was prepared in each reaction. Precursors were first loaded into carbon-coated tubes in a nitrogen-filled glovebox and then flame-sealed under dynamic vacuum (10^–3^ Torr). The sealed reaction vessels were placed into a programmable furnace and heated to 450 °C at a rate of 100 °C/h and then dwelled for 5 h. The temperature was then increased to 1000 °C at the same rate, and the reactions were dwelled at that temperature for 72 h. The samples were then cooled to 500 °C at a rate of 10 °C/h, followed by radiative cooling to room temperature. The resulting ingots were pulverized into fine powders prior to pXRD and UV–vis characterization, and the samples were stored in a nitrogen-filled glovebox.

### Single-Crystal X-ray Diffraction

Single crystals of all new compounds were coated with paratone oil, mounted on a Nylon loop, and measured on a Rigaku Synergy Custom system with a HyPix-Arc 150 diffractometer at 40 kV and 30 mA. Frames were collected at 173 K using Oxford Cryo-stream for cooling crystals during data collection. The radiation source was Cu Kα radiation (λ = 1.5406 Å) for all samples. Absorption corrections were applied using both multiscan and numerical methods based on crystal shapes; for a subset of crystals, numerical absorption correction yielded improved refinement statistics and was adopted as the final correction, while multiscan correction was retained for the remaining crystals. Space group assignments were based on systematic absences, normalized structure factor statistics (*E* statistics), agreement factors for equivalent reflections, and successful refinement of the structure. The structures were solved by direct methods, expanded through successive difference Fourier maps using SHELXT, and refined against all data using the SHELXL-2014 software package as implemented in Olex2. Weighted *R* factors, *R*_w_, and all goodness-of-fit indicators are based on *F* ^2^. Summary diffraction and refinement statistics can be found in the Supporting files.

### Powder X-ray Diffraction

Powder X-ray diffraction patterns were collected on a Bruker D2 PHASER equipped with a Cu Kα X-ray source (λ = 1.5406 Å) with a voltage of 30 kV and a current of 15 mA. Simulated pXRD patterns and visualization of the crystal structures were done using the Vesta software package.

### UV–Vis Spectroscopy

Absorption data for the ACuHfQ_3_ powders were collected in diffuse-reflectance mode on a Shimadzu UV-3600 UV–Vis–NIR spectrophotometer. BaSO_4_ was used as a reflectance standard. The reflectance data were transformed to absorption using the Kubelka–Munk function, *F*(*R*), defined as (1 – *R*)^2^/2*R*, where *R* is the diffuse reflectance. The direct-gap formulation of the Tauc plots were constructed by plotting (*F*(*R*)*h*ν)^2^ vs *h*ν, and the (direct) band gaps were determined by extrapolating the linear regions of the plots to the energy axis.

### Photoluminescence Spectroscopy

Photoluminescence spectra were obtained by using the LabRAM Soleil Raman microscope (Horiba) equipped with a 532 nm laser. The spectrometer was calibrated by using a polystyrene reference. The laser power was tested on all of the samples to ensure no modification of the material by the laser. The grating used for the measurement was 600 gr/mm, and the confocal hole was set to 500 μm. Data were collected with the 50× LWD objective lens (numerical aperture (NA) 0.5), 30 s acquisition time, 2 accumulations between 540 and 1100 nm with a laser power between 0.6 and 0.96 mW.

### Band Structure Calculations

We carried out density functional theory (DFT)^41,42^ calculations using the Vienna Ab initio Simulation Package (VASP)^54–56^ to investigate the electronic band structures and density of states for the ten new ACuHfQ_3_ compounds. Structural optimizations and electronic structure calculations were performed using the projector-augmented wave (PAW)^57^ method with plane-wave basis sets. The generalized gradient approximation (GGA)^58^ with the Perdew–Burke–Ernzerhof (PBE) exchange–correlation functional^59^ was employed throughout. A kinetic energy cutoff of 520 eV was used for all calculations. Brillouin-zone sampling was carried out using *k*-point meshes corresponding to a minimum reciprocal-space spacing of 0.15 Å^–1^. Convergence thresholds were set to 10^–5^ eV for energy and 10^–2^ eV/Å for forces.

### Machine Learning Model

For the classification of polyhedral building-block sequences (OOTT versus OTOT), we employed the SISSO (Sure Independence Screening and Sparsifying Operator) framework^51^ in its classification mode with a single task. The training set contained 24 labeled compounds, split into two classes with 15 and 9 samples with OTOT and OOTT arrangement, respectively. We used the ionic radii of the four constituent elements, sourced from the Mendeleev database,^52^ and constructed an expanded feature space using only basic arithmetic operators (+), (−), (*), (/) with a maximum feature complexity of two operations. Candidate features were filtered by magnitude to remove numerically ill-conditioned terms, and 100 features were retained after sure independence screening. We then identified an interpretable two-dimensional descriptor using L0 sparsifying optimization, selecting the top-ranked model as demonstrated in the main text.

## Supplementary Material



## Notes

Optical images; pXRD and UV–vis data; band structures with density of states; and full crystallographic data tables (PDF)

## References

[1] Liao J. H., Marking G. M., Hsu K. F., Matsushita Y., Ewbank M. D., Borwick R., Cunningham P., Rosker M. J., Kanatzidis M. G. (2003). α- and β-A_2_Hg_3_M_2_S_8_ (A = K, Rb; M = Ge, Sn):
Polar Quaternary Chalcogenides with Strong Nonlinear Optical Response. J. Am. Chem. Soc..

[2] Wessler G. C., Zhu T., Sun J.-P., Harrell A., Huhn W. P., Blum V., Mitzi D. B. (2018). Band Gap
Tailoring and Structure-Composition Relationship
within the Alloyed Semiconductor Cu_2_BaGe_1–*x*
_Sn_
*x*
_Se_4_. Chem. Mater..

[3] Lekse J. W., Moreau M. A., McNerny K. L., Yeon J., Halasyamani P. S., Aitken J. A. (2009). Second-Harmonic
Generation and Crystal Structure of
the Diamond-like Semiconductors Li_2_CdGeS_4_ and
Li_2_CdSnS_4_. Inorg. Chem..

[4] Akopov G., Hewage N. W., Yox P., Viswanathan G., Lee S. J., Hulsebosch L. P., Cady S. D., Paterson A. L., Perras F. A., Xu W. (2021). Synthesis-enabled exploration
of chiral and polar multivalent quaternary sulfides. Chem. Sci..

[5] Hodges J. M., Xia Y., Malliakas C. D., Alexander G. C. B., Chan M. K. Y., Kanatzidis M. G. (2018). Two-Dimensional
CsAg5Te3–xSx Semiconductors: Multi-anion Chalcogenides with
Dynamic Disorder and Ultralow Thermal Conductivity. Chem. Mater..

[6] Perryman J. T., Velázquez J. M. (2021). Design
Principles for Multinary Metal Chalcogenides:
Toward Programmable Reactivity in Energy Conversion. Chem. Mater..

[7] Viti M. A., Li Z., Laing C. C., Wolverton C., Kanatzidis M. G. (2025). Compositional
Design Guides Property Control in A_2_M_2+*x*
_Ti_1–(*x*/4)_Q_4_ Semiconductors. J. Am. Chem. Soc..

[8] Viti M. A., Li Z., Liu Z., Wolverton C., Kanatzidis M. G. (2025). Dimensional
Evolution Guides Property Control in the AnCu_4–*n*
_TiS_4_ Semiconductor Series. J. Am. Chem. Soc..

[9] Tassanov A., Lee H., Xia Y., Hodges J. M. (2024). Rational Pathways to Ordered Multianion
Chalcogenides Using Retrosynthetic Crystal Chemistry. J. Am. Chem. Soc..

[10] Jana S., Gabilondo E. A., Mongkhonratanachai M., Zhang Y., Halasyamani P. S., Maggard P. A. (2024). Large Mid-Infrared Second-Harmonic Generation in Eu­(II)-Based
Quaternary Chalcogenides. Chem. Mater..

[11] Wu K., Zhang B., Yang Z., Pan S. (2017). New Compressed Chalcopyrite-like
Li2BaMIVQ4 (MIV = Ge, Sn; Q = S, Se): Promising Infrared Nonlinear
Optical Materials. J. Am. Chem. Soc..

[12] Yang Y., Wu K., Wu X., Zhang B., Gao L. (2020). A new family of quaternary
thiosilicates SrA_2_SiS_4_ (A = Li, Na, Cu) as promising
infrared nonlinear optical crystals. J. Mater.
Chem. C.

[13] McWhorter T. M., Zhang W., Shen Y., Blum V., Mitzi D. B. (2025). Out-of-Plane
Rashba Spin-Splitting and Structure-Property Relationships in Layered
Cu_2_AMVSe_4_ Chalcogenides (A = K, Rb, Cs; MV =
V, Nb, Ta). Chem. Mater..

[14] Wu Y., Näther C., Bensch W. (2004). Low Dimensional Materials: Syntheses,
Structures, and Optical Properties of Rb_2_CuTaS_4_, Rb_2_CuTaSe_4_, RbCu_2_TaSe_4_, K_3_Ag_3_Ta_2_Se_8_, and Rb_3_AgTa_2_Se_12_. Z.
Naturforsch., B: J. Chem. Sci..

[16] Xia W., Tang L., Sun H., Zhang C., Ho K.-M., Viswanathan G., Kovnir K., Wang C.-Z. (2023). Accelerating materials
discovery using integrated deep machine learning approaches. J. Mater. Chem. A.

[17] Himanen L., Geurts A., Foster A. S., Rinke P. (2019). Data-Driven Materials
Science: Status, Challenges, and Perspectives. Adv. Sci..

[18] Saal J. E., Kirklin S., Aykol M., Meredig B., Wolverton C. (2013). Materials
Design and Discovery with High-Throughput Density Functional Theory:
The Open Quantum Materials Database (OQMD). JOM.

[19] Pal K., Xia Y., He J., Wolverton C. (2019). Intrinsically
Low Lattice Thermal
Conductivity Derived from Rattler Cations in an AMM′Q_3_ Family of Chalcogenides. Chem. Mater..

[20] Gautier R., Zhang X., Hu L., Yu L., Lin Y., Sunde T. O. L., Chon D., Poeppelmeier K. R., Zunger A. (2015). Prediction and accelerated laboratory discovery of
previously unknown 18-electron ABX compounds. Nat. Chem..

[21] Jain A., Hautier G., Moore C. J., Ping Ong S., Fischer C. C., Mueller T., Persson K. A., Ceder G. (2011). A high-throughput infrastructure
for density functional theory calculations. Comput. Mater. Sci..

[22] Zhu H., Hautier G., Aydemir U., Gibbs Z. M., Li G., Bajaj S., Pöhls J.-H., Broberg D., Chen W., Jain A. (2015). Computational
and experimental investigation of TmAgTe_2_ and XYZ_2_ compounds, a new group of thermoelectric
materials identified by first-principles high-throughput screening. J. Mater. Chem. C.

[23] Pal K., Xia Y., Shen J., He J., Luo Y., Kanatzidis M. G., Wolverton C. (2021). Accelerated
discovery of a large family of quaternary
chalcogenides with very low lattice thermal conductivity. npj Comput. Mater..

[24] Koscielski L. A., Ibers J. A. (2012). The Structural Chemistry
of Quaternary Chalcogenides
of the Type AMM’Q_3_. Z. Anorg.
Allg. Chem..

[25] Pal K., Hua X., Xia Y., Wolverton C. (2020). Unraveling the Structure-Valence-Property
Relationships in AMM′Q_3_ Chalcogenides with Promising
Thermoelectric Performance. ACS Appl. Energy
Mater..

[26] Singh, N. ; Ubaid, M. ; Nayak, P. K. ; He, J. ; Ghosh, D. ; Wolverton, C. ; Pal, K. Data-driven Discovery of Novel High-performance Quaternary Chalcogenide Photovoltaics. 2025, arXiv:2507.15430. arXiv.org e-Print archive. https://arxiv.org/abs/2507.15430.

[27] Rohj R. K., Bhui A., Sett S., Ghosh A., Biswas K., Sarma D. D. (2025). Ultralow Thermal Conductivity Approaching the Disordered
Limit in Crystalline TlCuZrSe_3_. Chem.
Mater..

[28] Laing C. C., Weiss B. E., Pal K., Quintero M. A., Xie H., Zhou X., Shen J., Chung D. Y., Wolverton C., Kanatzidis M. G. (2022). ACuZrQ_3_ (A = Rb, Cs; Q = S, Se, Te): Direct
Bandgap Semiconductors and Metals with Ultralow Thermal Conductivity. Chem. Mater..

[29] Cao B., Adutwum L. A., Oliynyk A. O., Luber E. J., Olsen B. C., Mar A., Buriak J. M. (2018). How To
Optimize Materials and Devices via Design of
Experiments and Machine Learning: Demonstration Using Organic Photovoltaics. ACS Nano.

[30] Gzyl A. S., Oliynyk A. O., Mar A. (2020). Half-Heusler
Structures with Full-Heusler
Counterparts: Machine-Learning Predictions and Experimental Validation. Cryst. Growth Des..

[31] Oliynyk A. O., Adutwum L. A., Rudyk B. W., Pisavadia H., Lotfi S., Hlukhyy V., Harynuk J. J., Mar A., Brgoch J. (2017). Disentangling Structural Confusion through Machine
Learning: Structure Prediction and Polymorphism of Equiatomic Ternary
Phases ABC. J. Am. Chem. Soc..

[32] Salzbrenner P. T., Joo S. H., Conway L. J., Cooke P. I. C., Zhu B., Matraszek M. P., Witt W. C., Pickard C. J. (2023). Developments and
further applications of ephemeral data derived potentials. J. Chem. Phys..

[33] Schrier J., Norquist A. J., Buonassisi T., Brgoch J. (2023). In Pursuit of the Exceptional:
Research Directions for Machine Learning in Chemical and Materials
Science. J. Am. Chem. Soc..

[34] Mansuetto M. F., Keane P. M., Ibers J. A. (1992). Synthesis,
structure, and conductivity
of the new group IV chalcogenides, KCuZrQ_3_ (Q = S, Se,
Te). J. Solid State Chem..

[35] Lemoine P., Carre D., Guittard M. (1986). Structure
du sulfure d’europium
et de cuivre Eu_2_CuS_3_. Acta Crystallogr., Sect. C: Struct. Chem..

[36] Mansuetto M. F., Keane P. M., Ibers J. A. (1993). Synthesis
and Structures of the New
Group IV Chalcogenides NaCuTiS_3_ and NaCuZrQ_3_ (Q = S, Se, Te). J. Solid State Chem..

[37] Pell M. A., Ibers J. A. (1996). Synthesis and structure of TlCuTiTe_3_. J. Alloys Compd..

[38] Berseneva A. A., Klepov V. V., Pal K., Seeley K., Koury D., Schaeperkoetter J., Wright J. T., Misture S. T., Kanatzidis M. G., Wolverton C. (2022). Transuranium Sulfide via the Boron Chalcogen
Mixture Method and Reversible Water Uptake in the NaCuTS_3_ Family. J. Am. Chem. Soc..

[39] Park S. C., Kuratieva N. V., Pomelova T. A., Naumov N. G. (2022). SYNTHESIS AND CRYSTAL
STRUCTURE OF CsLnZnS_3_ (Ln = Gd, Dy). J. Struct. Chem..

[40] Klein J., Kampermann L., Mockenhaupt B., Behrens M., Strunk J., Bacher G. (2023). Limitations of the
Tauc Plot Method. Adv. Funct. Mater..

[41] Hohenberg P., Kohn W. (1964). Inhomogeneous Electron
Gas. Phys. Rev..

[42] Kohn W., Sham L. J. (1965). Self-Consistent
Equations Including Exchange and Correlation
Effects. Phys. Rev..

[43] Vajenine G. V., Hoffmann R. (1996). Compounds Containing
Copper–Sulfur Layers: Electronic
Structure, Conductivity, and Stability. Inorg.
Chem..

[44] Hodges J. M., Xia Y., Malliakas C. D., Slade T. J., Wolverton C., Kanatzidis M. G. (2020). Mixed-Valent Copper Chalcogenides: Tuning Structures
and Electronic Properties Using Multiple Anions. Chem. Mater..

[45] Tassanov A., Lee H., Xia Y., Hodges J. M. (2024). Layered
NaBa_2_M_3_Q_3_(Q_2_) (M = Cu
or Ag; Q = S or Se) Chalcogenides
and Local Ordering in Their Mixed-Anion Compositions. Inorg. Chem..

[46] Perdew J. P. (2009). Density
functional theory and the band gap problem. Int. J. Quantum Chem..

[47] Zhao W., Ribeiro R. M., Toh M., Carvalho A., Kloc C., Castro Neto A. H., Eda G. (2013). Origin of Indirect Optical Transitions
in Few-Layer MoS_2_, WS_2_, and WSe_2_. Nano Lett..

[48] Keane P. M., Ibers J. A. (1991). Synthesis of K_4_M_3_Te_17_ (M = zirconium, hafnium) and
the structure of potassium hafnium
telluride, K_4_Hf_3_Te_17_, a new one-dimensional
solid-state ternary polytelluride. Inorg. Chem..

[49] Klepp K. O., Gurtner D. (1996). Quaternary chalcogenides of the IVa metals with layered
structures: preparation and crystal structures of TlCuTIVQ_3_ (T = Zr, Hf; Q = S, Se) and their relation to the Re_3_B structure type. J. Alloys Compd..

[50] Sankar C. R., Bangarigadu-Sanasy S., Assoud A., Kleinke H. (2011). Crystal Structures,
Electronic Structures, and Physical Properties of Tl_4_MQ_4_ (M = Zr or Hf; Q = S or Se). Inorg.
Chem..

[51] Ouyang R., Curtarolo S., Ahmetcik E., Scheffler M., Ghiringhelli L. M. (2018). SISSO: A compressed-sensing method for identifying
the best low-dimensional descriptor in an immensity of offered candidates. Phys. Rev. Mater..

[52] Mentel, L. Mendeleev - A Python Package with Properties of Chemical Elements, Ions, Isotopes and Methods to Manipulate and Visualize Periodic Table, 2021.

[53] McCarthy T. J., Kanatzidis M. G. (1995). Synthesis
in Molten Alkali Metal Polyselenophosphate
Fluxes: A New Family of Transition Metal Selenophosphate Compounds,
A_2_MP_2_Se_6_ (A = K, Rb, Cs; M = Mn,
Fe) and A_2_M’_2_P_2_Se_6_ (A = K, Cs; M’ = Cu, Ag). Inorg. Chem..

[54] Kresse G., Hafner J. (1993). Ab initio molecular dynamics for liquid metals. Phys. Rev. B.

[55] Kresse G., Hafner J. (1994). Ab initio molecular-dynamics simulation of the liquid-metal--amorphous-semiconductor
transition in germanium. Phys. Rev. B.

[56] Kresse G., Furthmüller J. (1996). Efficient iterative schemes for ab initio total-energy
calculations using a plane-wave basis set. Phys.
Rev. B.

[57] Blöchl P. E. (1994). Projector
augmented-wave method. Phys. Rev. B.

[58] Perdew J. P., Burke K., Wang Y. (1996). Generalized gradient approximation
for the exchange-correlation hole of a many-electron system. Phys. Rev. B.

[59] Perdew J. P., Burke K., Ernzerhof M. (1996). Generalized
Gradient Approximation
Made Simple. Phys. Rev. Lett..

