# Supplementary material for: Structure–Optical Property Relationships in AMM′Q3 Chalcogenides
Source: Chem Mater. 2026 Jun 10;38(12):5938–46. doi: 10.1021/acs.chemmater.6c00212 (PMC13296268; doi:10.1021/acs.chemmater.6c00212)
Supplement: Supplementary file 1 [file cm6c00212_si_001.pdf]

Supplemental Information for:

Structure-Optical Property Relationships in AMM'Q_3 Chalcogenides

Ayat Tassanov[†], Huiju Lee[§], Daniel Spainhour[†], Yi Xia[§], and James M. Hodges[†*]

[†] Department of Chemistry, The Pennsylvania State University, University Park, Pennsylvania 16802, United States.

[§] Department of Mechanical and Materials Engineering, Portland State University, Portland, Oregon 97201, United States.

Corresponding author e-mail address: hodges@psu.edu

Experimental Methods

Materials. The following materials were used as received: copper chunks (Cu, 99.999%), sulfur chunks (S, 99.999%), selenium shot (Se, 99.999%), and tellurium chunks (Te, 99.999%) were purchased from American Elements. Sodium chunks (Na, 98%), potassium chunks (K, 98%), and cesium metal (Cs, 99.8 %) were purchased from Fisher Scientific. Rubidium metal (Rb, 99.9%) was purchased from Strem Chemicals. The following materials were dried in a vacuum oven set to 200 °C for 24 h and then stored in a nitrogen-filled glovebox: sodium chloride (NaCl, 99%), potassium chloride (KCl, 99%), rubidium chloride (RbCl, 99%), and cesium chloride (CsCl, 99%) were purchased from Sigma Aldrich.

Synthesis of Binary Alkali Chalcogenides. A_2Q (A = Rb, Cs; Q = S, Se, Te) binary precursors were pre-synthesized by reacting the appropriate alkali metal with and chalcogen in liquid ammonia, as described previously. In a typical 20 g reaction, Cs or Rb metal was gently heated in a nitrogen-filled glove box and then pipetted into a 250 mL three-neck round-bottom flask equipped with a glass stir bar. The flask was then capped with a glass condenser with a Teflon valve and the airtight vessel was transferred to a Schlenk line that was then purged with nitrogen. Approximately 150 mL of ammonia gas was condensed into the flask using a dry ice/acetone bath (-77 °C) dissolving the alkali metal to form a dark blue solution. Finely ground chalcogen was carefully added under increased flow of nitrogen and the mixture was stirred for several hours. 5% excess of the stoichiometric amount of chalcogen was used to minimize presence of unreacted alkali metal in final product. After the product is formed, excess ammonia is evaporated, and the product is dried under a vacuum overnight before being stored in a nitrogen-filled glovebox for future use.

Synthesis of $ACuHfQ_3$ Crystals. The synthesis of novel compounds was performed using the flux method. Carbon-coated tubes were filled with 1000 mg of stoichiometric amounts of elemental precursors and topped with 2000 mg of the appropriate flux salt, then under dynamic vacuum (10^{-3} Torr). Tubes were heated in a programmable furnace to 450°C at 100°C per hour and held there for 5 hours. The temperature then increased to 1000°C and maintained for 24 hours for complete melting of the flux. The mixture cooled down to 600°C at 10°C per hour to allow crystal growth before natural cooling. A similar approach was used for sodium (Na), potassium (K), rubidium (Rb), and cesium (Cs) analogs using NaCl, KCl, RbCl, and CsCl salt fluxes, respectively. Images of the ingots and representative crystals are included in the supplementary files. Most crystals, reaching up to 5 mm in length, were separated from the flux by hand, and single-crystal X-ray diffraction was conducted to identify their structures.

Direct synthesis of new compounds for bulk characterization. All presented compounds were synthesized directly using stoichiometric amounts of precursors to match a ratio of A: M: Hf: Q=1:1:1:3. (where A=alkali, M= Cu and Q=S, Se, Te) All precursors were loaded into the carbon-coated tubes inside the nitrogen-filled glovebox and sealed under under dynamic vacuum (10^{-3} Torr). To synthesize, Cs and Rb analogs, alkali binaries were used and the ratios for all reactions are A_2Q: M: Hf: Q =1:2:2:5. Sealed tubes were then placed into the programmable oven and samples were annealed at 450 °C for 5 h. The heating rate was 100 C/hour. Then the temperature increased to 1000 °C at the same rate and stayed there for another 72 hours. Finally, the samples were cooled to 500C at a rate of 10 °C/hour with natural cooling afterward. All powders were stored in a nitrogen-filled glovebox and only taken out for characterization.

Powder X-ray diffraction. Powder X-ray diffraction patterns were collected on a Bruker D2 PHASER equipped with a Cu Kα X-ray source (λ = 1.5406 Å) with a voltage of 30 kV and a current of 15 mA. Simulated PXRD patterns and visualization of the crystal structures were done with the Vesta software.

Single-crystal X-ray diffraction. Single crystals of all new compounds were coated with Paratone oil, mounted on a Nylon loop, and transferred to a Rigaku, Synergy Custom system with HyPix-Arc 150 diffractometer at 40 kV and 30 mA. Frames were collected at 173K using Oxford Cryo-stream for cooling crystals during data collection. The radiation source was Cu Kα radiation (λ =1.5406 Å) for all samples. Absorption corrections were applied using both multi-scan and numerical methods based on crystal shape; for a subset of crystals, numerical absorption correction yielded improved refinement statistics and was adopted as the final correction, while multi-scan correction was retained for the remaining crystals. Space-group assignments were based on systematic absences, normalized structure factor statistics (*E* statistics), agreement factors for equivalent reflections, and successful refinement of the structure. The structures were solved by direct methods, expanded through successive difference Fourier maps using SHELXT, and refined against all data using the SHELXL-2014 software package as implemented in Olex2. Weighted *R* factors, R_w, and all goodness-of-fit indicators are based on F^2. Summary diffraction and refinement statistics can be found in the supplementary files.

Photoluminescence Spectroscopy. The photoluminescence measurements were obtained using the LabRAM Soleil Raman microscope (Horiba) equipped with a 532 nm laser. The spectrometer was calibrated using a polystyrene reference. The laser power was tested on all the samples to ensure no modification of the material by the laser. The grating used for the measurement was 600 gr/mm and the confocal hole was set to 500 micrometers. Data was collected with the 50x LWD

objective lens (NA 0.5), 30s acquisition time, 2 accumulations between 540 nm and 1100 nm with a laser power between 0.6 mW and 0.96 mW.

Band Structure Calculations. We carried out density functional theory (DFT) [1-2] calculations using the Vienna Ab initio Simulation Package (VASP) [3-6] to investigate the electronic band structures and density of states for the ten new ACuHfQ3 compounds. Structural optimizations and electronic structure calculations were performed using the projector-augmented wave (PAW) [7] method with plane-wave basis sets. The generalized gradient approximation (GGA) [8] with the Perdew–Burke–Ernzerhof (PBE) exchange-correlation functional [9] was employed throughout. A kinetic energy cutoff of 520 eV was used for all calculations. Brillouin-zone sampling was carried out using k-point meshes corresponding to a minimum reciprocal-space spacing of 0.15 $Å^{-1}$. Convergence thresholds were set to 10^{-5} eV for energy and 10^{-2} eV/Å for forces.

Machine Learning Model. For the classification of polyhedral building-block sequences (OOTT versus OTOT), we employed the SISSO (Sure Independence Screening and Sparsifying Operator) framework [10] in its classification mode with a single task. The training set contained 24 labeled compounds, split into two classes with 15 and 9 samples with OTOT and OOTT arrangement, respectively. We used the ionic radii of the four constituent elements, sourced from the Mendeleev database [11], and constructed an expanded feature space using only basic arithmetic operators (+) , (−) , (∗) , (/) with a maximum feature complexity of two operations. Candidate features were filtered by magnitude to remove numerically ill-conditioned terms, and 100 features were retained after sure-independence screening. We then identified an interpretable two-dimensional descriptor using $L0$ sparsifying optimization, outputting the top-ranked model as demonstrated in the main text.

[1] Hohenberg, P.; Kohn, W. Inhomogeneous Electron Gas. Physical Review 1964, 136 (3B), B864-B871. DOI: 10.1103/PhysRev.136.B864.

[2] Kohn, W.; Sham, L. J. Self-Consistent Equations Including Exchange and Correlation Effects. Physical Review 1965, 140 (4A), A1133-A1138. DOI: 10.1103/PhysRev.140.A1133.

[3] Kresse, G.; Hafner, J. Ab initio molecular dynamics for liquid metals. Physical Review B 1993, 47 (1), 558-561. DOI: 10.1103/PhysRevB.47.558.

[4] Kresse, G.; Hafner, J. Ab initio molecular-dynamics simulation of the liquid-metal--amorphous-semiconductor transition in germanium. Physical Review B 1994, 49 (20), 14251-14269. DOI: 10.1103/PhysRevB.49.14251.

[5] Kresse, G.; Furthmüller, J. Efficiency of ab-initio total energy calculations for metals and semiconductors using a plane-wave basis set. Computational Materials Science 1996, 6 (1), 15-50. DOI: https://doi.org/10.1016/0927-0256(96)00008-0.

[6] Kresse, G.; Furthmüller, J. Efficient iterative schemes for ab initio total-energy calculations using a plane-wave basis set. Physical Review B 1996, 54 (16), 11169. DOI: https://doi.org/10.1103/PhysRevB.54.11169

[7] Blöchl, P. E. Projector augmented-wave method. Physical Review B 1994, 50 (24), 17953-17979. DOI: 10.1103/PhysRevB.50.17953.

[8] Perdew, J. P.; Burke, K.; Wang, Y. Generalized gradient approximation for the exchange-correlation hole of a many-electron system. Physical Review B 1996, 54 (23), 16533-16539. DOI: 10.1103/PhysRevB.54.16533.

[9] Perdew, J. P.; Burke, K.; Ernzerhof, M. Generalized Gradient Approximation Made Simple. Physical Review Letters 1996, 77 (18), 3865-3868. DOI: 10.1103/PhysRevLett.77.3865.

[10] Ouyang, R.; Curtarolo, S.; Ahmetcik, E.; Scheffler, M.; Ghiringhelli, L. M. SISSO: A compressed-sensing method for identifying the best low-dimensional descriptor in an immensity of offered candidates. *Physical Review Materials* 2018, *2* (8), 083802. DOI: 10.1103/PhysRevMaterials.2.083802.

[11] Mentel, L. *Mendeleev - A Python Package with Properties of Chemical Elements, Ions, Isotopes and Methods to Manipulate and Visualize Periodic Table*. 2021. https://github.com/lmmentel/mendeleev.

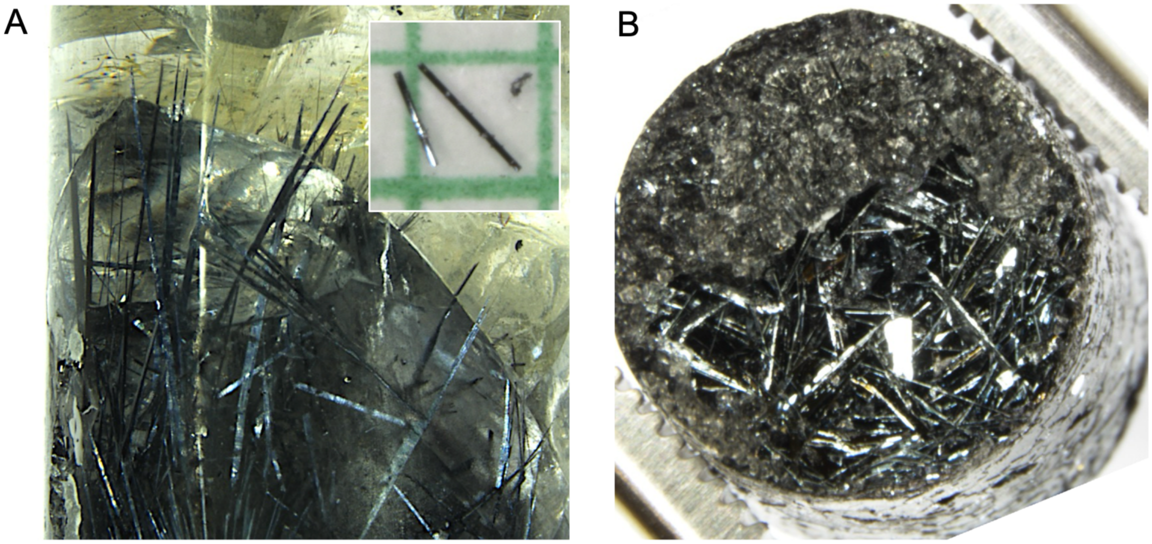

Figure S1. Representative optical images of $ACuHfQ_3$ crystals grown in alkali chloride (ACl) flux and bulk ingot prepared using self-flux approach. (A) $CsCuHfSe_3$ crystals suspended in CsCl with isolated crystals shown in inset, and (B) ingot of $RbCuHfSe_3$.

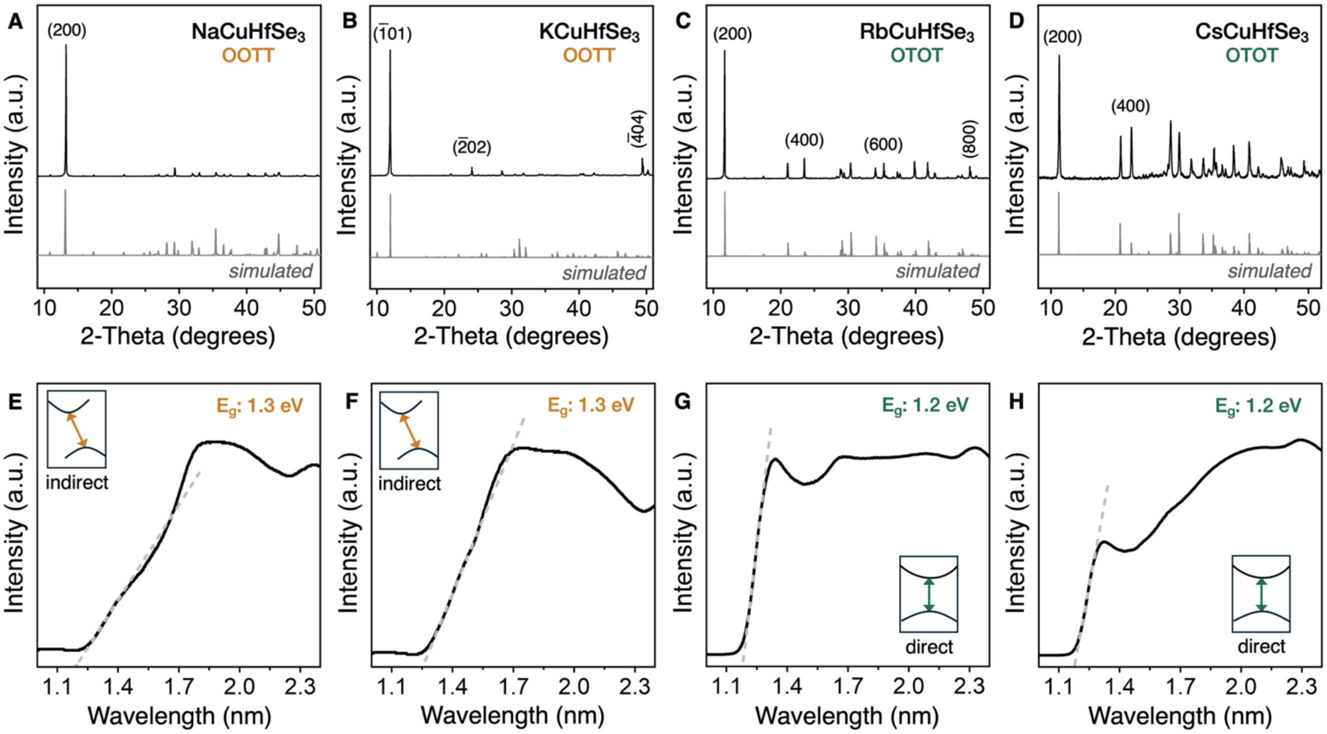

Figure S2. Powder X-ray diffractograms for the $ACuHfSe_3$ (A = Na, K, Rb, Cs) compounds including (A) $NaCuHfSe_3$, (B) $KCuHfSe_3$, (C) $RbCuHfSe_3$, and (D) $CsCuHfSe_3$. In each case, the diffractograms of the sulfides are consistent with the simulated patterns calculated using the refined structures obtained from SCXRD analyses. The discrepancy in peak intensities is attributed to preferred orientation and reflections that run parallel with [$CuHfSe_3$] layers are indexed for clarity. Absorption spectra for (E) $NaCuHfSe_3$, (F) $KCuHfSe_3$, (G) $RbCuHfSe_3$, and (H) $CsCuHfSe_3$, with (G, H) showing noticeably sharper onset energies consistent with direct-gap transitions.

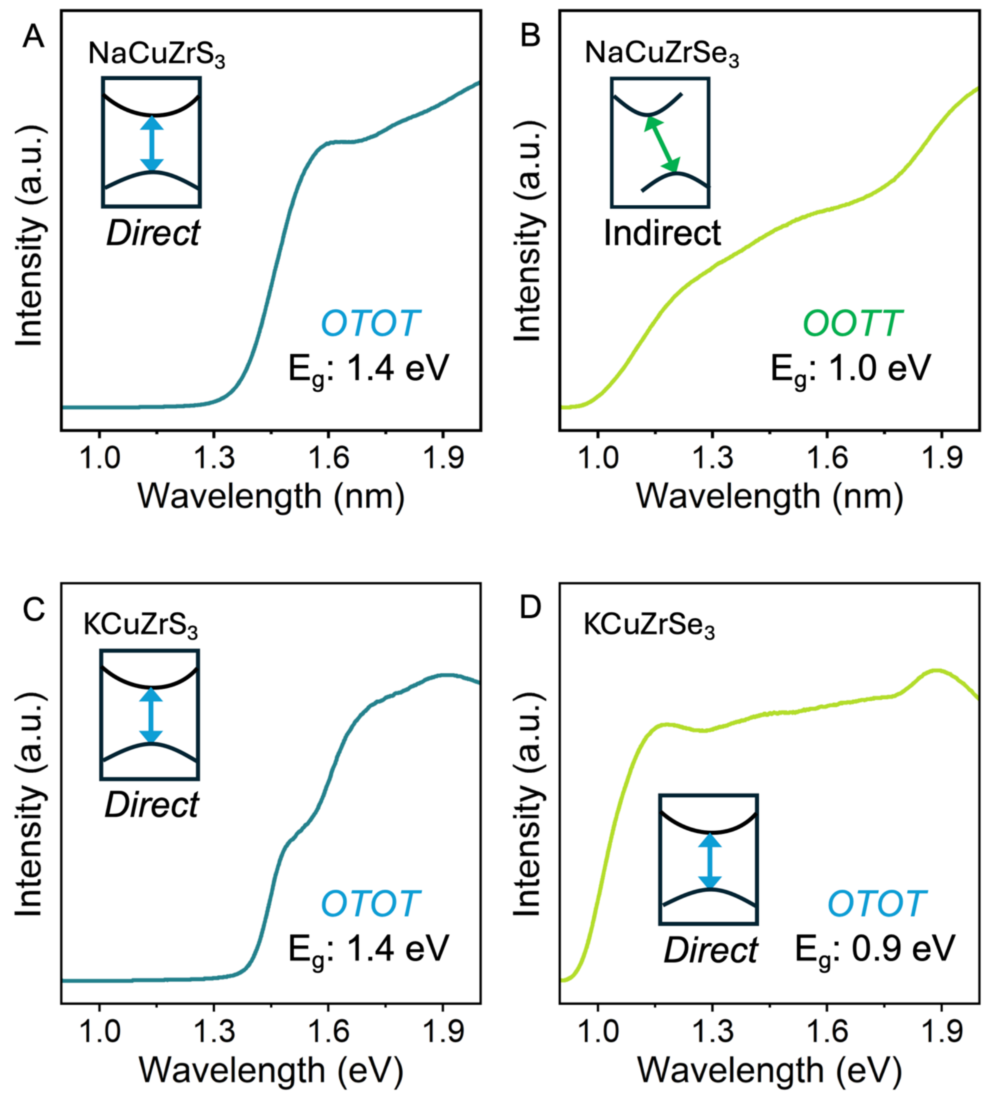

Figure S3. Absorption spectra for $ACuZrQ_3$ (A = Na, K; Q = S, Se) compounds including (A) $NaCuZrS_3$, (B) $NaCuZrSe_3$, (C) $KCuZrS_3$, and (D) $KCuZrSe_3$.

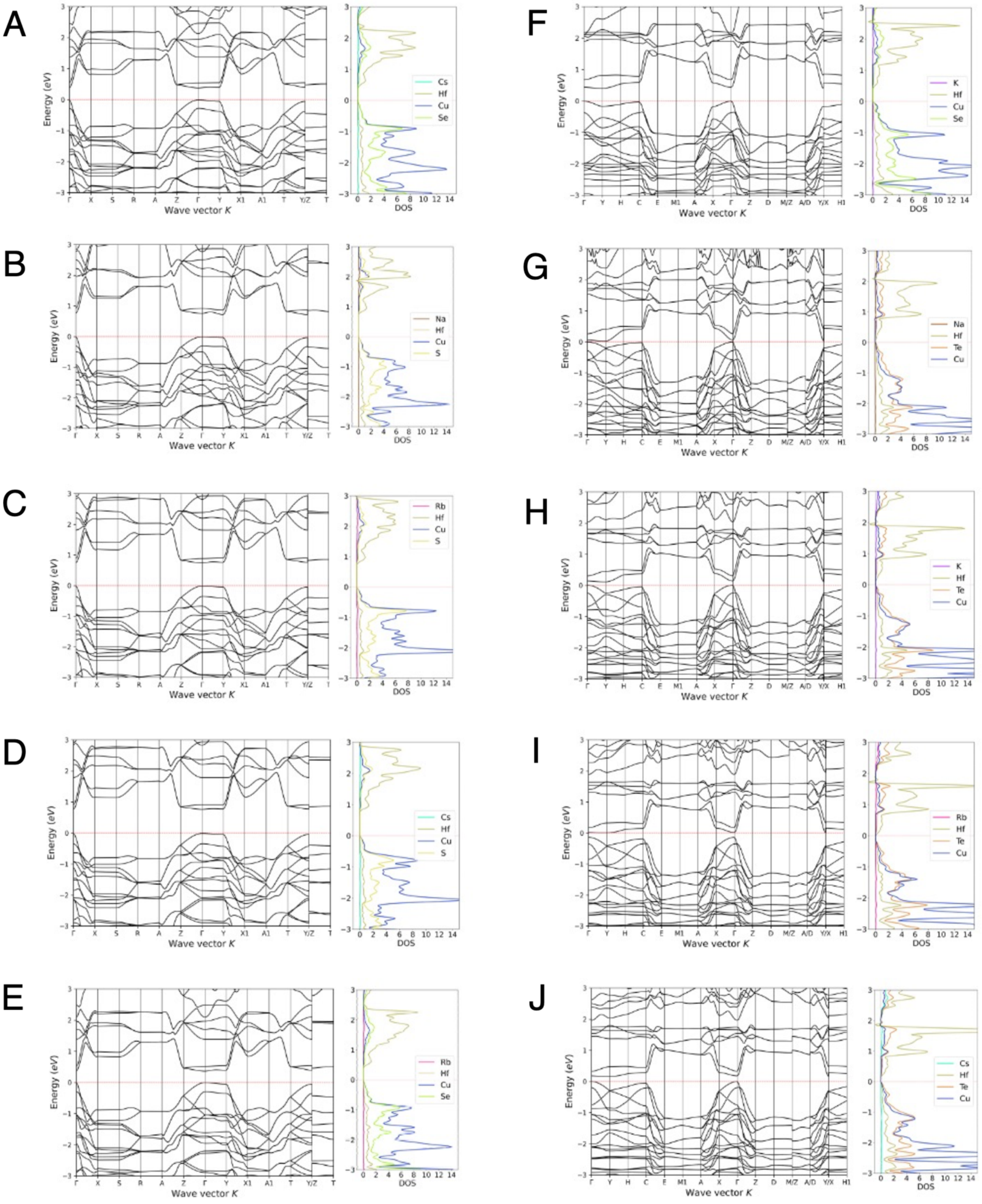

Figure S4. Band structure diagrams for $ACuHfQ_3$ along with density of states (DOS) including (A) $CsCuHfSe_3$, (B) $NaCuHfS_3$, (C) $RbCuHfS_3$, (D) $CsCuHfS_3$, (E) $RbCuHfSe_3$, (F) $KCuHfSe_3$, (G) $NaCuHfTe_3$, (H) $KCuHfTe_3$, (I) $RbCuHfTe_3$, and (J) $CsCuHfTe_3$.

Table S1. Cu-Cu distances in $ACuHfQ_3$ compounds.

Compound	Space Group	Cu-Cu (Å)
$NaCuHfS_3$	*Pnma* (OOTT)	2.5922(2)
$RbCuHfS_3$	*Cmcm* (OTOT)	3.7261(2)
$CsCuHfS_3$	*Cmcm* (OTOT)	3.7443(2)
$KCuHfSe_3$	$P2_1/m$ (OOTT)	2.7124(5)
$RbCuHfSe_3$	*Cmcm* (OTOT)	3.8665(3)
$CsCuHfSe_3$	*Cmcm* (OTOT)	3.8818(1)
$NaCuHfTe_3$	$P2_1/m$ (OOTT)	2.8443(1)
$KCuHfTe_3$	$P2_1/m$ (OOTT)	2.9611(5)
$RbCuHfTe_3$	$P2_1/m$ (OOTT)	2.9333(9)
$CsCuHfTe_3$	$P2_1/m$ (OOTT)	2.8224(1)

Tables S2-S6. Full Crystallographic Data Tables for $NaCuHfS_3$ (ID 2465832).

Tables S7-S11. Full Crystallographic Data Tables for $RbCuHfS_3$ (ID 2465828).

Tables S12-S16. Full Crystallographic Data Tables for $CsCuHfS_3$ (ID 2465833).

Tables S17-S21. Full Crystallographic Data Tables for $KCuHfSe_3$ (ID 2465836).

Tables S22-S26. Full Crystallographic Data Tables for $RbCuHfSe_3$ (ID 2465827).

Tables S27-S31. Full Crystallographic Data Tables for $CsCuHfSe_3$ (ID 2465829).

Tables S32-S36. Full Crystallographic Data Tables for $NaCuHfTe_3$ (ID 2465834).

Tables S37-S41. Full Crystallographic Data Tables for $KCuHfTe_3$ (ID 2465830).

Tables S42-S46. Full Crystallographic Data Tables for $RbCuHfTe_3$ (ID 2465831).

Tables S47-S51. Full Crystallographic Data Tables for $CsCuHfTe_3$ (ID 2465835).

$NaCuHfS_3$ (S2-S6)

Table S2. Crystal data and structure refinement for $NaCuHfS_3$.

Identification code	2465832
Empirical formula	$NaCuHfS_3$
Formula weight	361.20
Temperature/K	173.00(10)
Crystal system	orthorhombic
Space group	Pnma
a/Å	12.8840(2)
b/Å	3.69080(10)
c/Å	9.8722(2)
α/°	90
β/°	90
γ/°	90
Volume/Å^3	469.445(17)
Z	4
ρ_{calc}g/cm^3	5.111
μ/mm^{-1}	57.532
F(000)	640.0
Crystal size/mm^3	0.216 × 0.06 × 0.033
Radiation	Cu Kα (λ = 1.54184)
2Θ range for data collection/°	11.292 to 149.776
Index ranges	-14 ≤ h ≤ 15, -3 ≤ k ≤ 4, -10 ≤ l ≤ 11
Reflections collected	1224
Independent reflections	500 [R_{int} = 0.0497, R_{sigma} = 0.0426]
Data/restraints/parameters	500/0/38
Goodness-of-fit on F^2	1.196
Final R indexes [I>=2σ (I)]	R_1 = 0.0538, wR_2 = 0.1544
Final R indexes [all data]	R_1 = 0.0550, wR_2 = 0.1561
Largest diff. peak/hole / e Å^{-3}	3.13/-2.17

Table S3. Fractional Atomic Coordinates (×10^4) and Equivalent Isotropic Displacement Parameters (Å^2×10^3) for $NaCuHfS_3$. U_{eq} is defined as 1/3 of the trace of the orthogonalised U_{IJ} tensor.

Atom	x	y	z	U(eq)	Wyckoff Position	Symmetry
Hf1	5122.6(6)	12500	3257.4(6)	12.3(5)	4c	.m.
Cu1	5098.5(16)	7500	913(2)	16.7(7)	4c	.m.
S1	3983(2)	7500	4344(3)	13.7(8)	4c	.m.
S2	6368(2)	7500	2568(3)	14.5(8)	4c	.m.
S3	3988(2)	12500	1232(3)	14.7(9)	4c	.m.
Na1	2307(4)	2500	4694(6)	25.3(14)	4c	.m.

Table S4. Anisotropic Displacement Parameters (Å^2×10^3) for $NaCuHfS_3$. The Anisotropic displacement factor exponent takes the form: $-2\pi^2[h^2a^{*2}U_{11}+2hka^*b^*U_{12}+\ldots]$.

Atom	U_{11}	U_{22}	U_{33}	U_{23}	U_{13}	U_{12}
Hf1	11.8(7)	9.6(8)	15.6(7)	0	-0.02(18)	0
Cu1	16.0(12)	16.6(18)	17.4(11)	0	-0.9(7)	0
S1	10.7(14)	15(2)	15.6(14)	0	-0.1(10)	0
S2	10.3(13)	16(2)	16.9(12)	0	-0.7(12)	0
S3	13.9(14)	14(2)	16.4(14)	0	-1.3(11)	0
Na1	14(2)	18(4)	43(3)	0	4(3)	0

Table S5. Bond Lengths for $NaCuHfS_3$.

Atom	Atom	Length/Å	Atom	Atom	Length/Å
Hf1	Cu1	2.9602(18)	Cu1	S3^5	2.356(2)
Hf1	Cu1^1	2.9602(18)	Cu1	Na1^6	3.444(5)
Hf1	S1^1	2.591(2)	Cu1	Na1^7	3.325(6)
Hf1	S1^2	2.634(3)	Cu1	Na1^8	3.444(5)
Hf1	S1	2.591(2)	S1	Na1^1	2.861(5)
Hf1	S2^1	2.538(2)	S1	Na1	2.861(5)
Hf1	S2	2.538(2)	S2	Na1^6	3.140(5)
Hf1	S3	2.477(3)	S2	Na1^9	3.197(7)
Hf1	Na1^1	3.895(6)	S2	Na1^8	3.140(5)
Cu1	Cu1^3	2.592(3)	S3	Na1^{10}	2.915(5)

Table S5. Bond Lengths for $NaCuHfS_3$.

Atom	Atom	Length/Å	Atom	Atom	Length/Å
Cu1	Cu1[4]	2.592(3)	S3	Na1[7]	2.915(5)
Cu1	S2	2.312(4)	Na1	Na1[1]	3.69080(10)
Cu1	S3[3]	2.423(4)	Na1	Na1[5]	3.69080(10)
Cu1	S3	2.356(2)			

[1]+X,1+Y,+Z; [2]1-X,2-Y,1-Z; [3]1-X,2-Y,-Z; [4]1-X,1-Y,-Z; [5]+X,-1+Y,+Z; [6]1/2+X,+Y,1/2-Z; [7]1/2-X,1-Y,-1/2+Z; [8]1/2+X,1+Y,1/2-Z; [9]1-X,1-Y,1-Z; [10]1/2-X,2-Y,-1/2+Z

Table S6. Bond Angles for $NaCuHfS_3$.

Atom	Atom	Atom	Angle/°	Atom	Atom	Atom	Angle/°
Cu1	Hf1	Cu1[1]	77.13(6)	Hf1	S1	Na1[1]	91.05(8)
Cu1	Hf1	Na1[1]	105.96(7)	Na1[1]	S1	Na1	80.34(17)
Cu1[1]	Hf1	Na1[1]	105.96(7)	Hf1	S2	Hf1[3]	93.29(10)
S1[2]	Hf1	Cu1	135.04(5)	Hf1	S2	Na1[6]	90.40(8)
S1	Hf1	Cu1[1]	139.61(7)	Hf1[3]	S2	Na1[5]	90.40(8)
S1[2]	Hf1	Cu1[1]	135.04(5)	Hf1[3]	S2	Na1[6]	149.50(14)
S1[1]	Hf1	Cu1[1]	82.74(6)	Hf1[3]	S2	Na1[9]	96.37(11)
S1[1]	Hf1	Cu1	139.61(7)	Hf1	S2	Na1[9]	96.37(11)
S1	Hf1	Cu1	82.74(6)	Hf1	S2	Na1[5]	149.50(14)
S1	Hf1	S1[2]	82.88(8)	Cu1	S2	Hf1[3]	75.08(8)
S1[1]	Hf1	S1	90.84(10)	Cu1	S2	Hf1	75.08(8)
S1[1]	Hf1	S1[2]	82.88(8)	Cu1	S2	Na1[5]	76.69(14)
S1[1]	Hf1	Na1[1]	47.26(6)	Cu1	S2	Na1[6]	76.69(14)
S1	Hf1	Na1[1]	47.26(6)	Cu1	S2	Na1[9]	167.27(19)
S1[2]	Hf1	Na1[1]	94.60(11)	Na1[6]	S2	Na1[9]	113.29(8)
S2	Hf1	Cu1	48.99(7)	Na1[5]	S2	Na1[9]	113.29(8)
S2	Hf1	Cu1[1]	104.50(6)	Na1[5]	S2	Na1[6]	71.98(14)
S2[1]	Hf1	Cu1	104.50(7)	Hf1	S3	Na1[10]	139.32(9)
S2[1]	Hf1	Cu1[1]	48.99(7)	Hf1	S3	Na1[4]	139.32(9)
S2	Hf1	S1	87.21(7)	Cu1[1]	S3	Hf1	75.50(10)
S2[1]	Hf1	S1[1]	87.21(7)	Cu1[8]	S3	Hf1	114.77(13)
S2[1]	Hf1	S1[2]	87.96(8)	Cu1	S3	Hf1	75.50(10)
S2[1]	Hf1	S1	170.80(9)	Cu1	S3	Cu1[8]	65.68(10)
S2	Hf1	S1[1]	170.80(9)	Cu1[1]	S3	Cu1	103.11(13)
S2	Hf1	S1[2]	87.96(8)	Cu1[1]	S3	Cu1[8]	65.68(10)
S2[1]	Hf1	S2	93.29(10)	Cu1[8]	S3	Na1[10]	79.79(14)
S2	Hf1	Na1[1]	133.33(5)	Cu1[1]	S3	Na1[4]	140.70(17)

Table S6. Bond Angles for $NaCuHfS_3$.

Atom	Atom	Atom	Angle/°	Atom	Atom	Atom	Angle/°
S2^{1}	Hf1	Na1^{1}	133.33(5)	Cu1^{8}	S3	Na1^{4}	79.79(14)
S3	Hf1	Cu1	50.41(5)	Cu1^{1}	S3	Na1^{10}	77.41(11)
S3	Hf1	Cu1^{1}	50.41(5)	Cu1	S3	Na1^{4}	77.42(11)
S3	Hf1	S1	89.97(9)	Cu1	S3	Na1^{10}	140.70(17)
S3	Hf1	S1^{1}	89.97(8)	Na1^{4}	S3	Na1^{10}	78.56(15)
S3	Hf1	S1^{2}	169.79(10)	Cu1^{11}	Na1	Cu1^{12}	44.99(8)
S3	Hf1	S2	99.01(8)	Cu1^{11}	Na1	Cu1^{13}	44.99(8)
S3	Hf1	S2^{1}	99.01(8)	Cu1^{12}	Na1	Cu1^{13}	64.80(11)
S3	Hf1	Na1^{1}	75.19(11)	Cu1^{11}	Na1	Na1^{3}	90.0
Hf1	Cu1	Hf1^{3}	77.13(6)	Cu1^{12}	Na1	Na1^{3}	57.60(5)
Hf1^{3}	Cu1	Na1^{4}	107.02(9)	Cu1^{13}	Na1	Na1^{3}	122.40(5)
Hf1	Cu1	Na1^{5}	117.48(11)	Cu1^{12}	Na1	Na1^{1}	122.40(5)
Hf1^{3}	Cu1	Na1^{6}	117.48(11)	Cu1^{11}	Na1	Na1^{1}	90.0
Hf1	Cu1	Na1^{6}	78.08(9)	Cu1^{13}	Na1	Na1^{1}	57.60(5)
Hf1^{3}	Cu1	Na1^{5}	78.08(9)	S1	Na1	Cu1^{11}	138.35(10)
Hf1	Cu1	Na1^{4}	107.02(9)	S1	Na1	Cu1^{13}	104.87(7)
Cu1^{7}	Cu1	Hf1^{3}	95.80(5)	S1^{3}	Na1	Cu1^{12}	104.87(7)
Cu1^{8}	Cu1	Hf1	95.79(5)	S1^{3}	Na1	Cu1^{11}	138.35(10)
Cu1^{8}	Cu1	Hf1^{3}	171.31(11)	S1	Na1	Cu1^{12}	161.4(2)
Cu1^{7}	Cu1	Hf1	171.31(11)	S1^{3}	Na1	Cu1^{13}	161.4(2)
Cu1^{7}	Cu1	Cu1^{8}	90.78(15)	S1	Na1	S1^{3}	80.34(17)
Cu1^{7}	Cu1	Na1^{5}	65.07(11)	S1^{3}	Na1	S2^{9}	72.50(15)
Cu1^{7}	Cu1	Na1^{6}	109.95(14)	S1	Na1	S2^{9}	72.50(15)
Cu1^{7}	Cu1	Na1^{4}	69.94(11)	S1	Na1	S2^{13}	79.96(11)
Cu1^{8}	Cu1	Na1^{5}	109.95(14)	S1	Na1	S2^{12}	125.7(2)
Cu1^{8}	Cu1	Na1^{4}	69.94(11)	S1^{3}	Na1	S2^{12}	79.96(11)
Cu1^{8}	Cu1	Na1^{6}	65.07(11)	S1^{3}	Na1	S2^{13}	125.7(2)
S2	Cu1	Hf1	55.94(7)	S1^{3}	Na1	S3^{14}	154.6(2)
S2	Cu1	Hf1^{3}	55.94(7)	S1^{3}	Na1	S3^{11}	94.97(8)
S2	Cu1	Cu1^{8}	124.12(10)	S1	Na1	S3^{11}	154.6(2)
S2	Cu1	Cu1^{7}	124.12(10)	S1	Na1	S3^{14}	94.97(8)
S2	Cu1	S3^{3}	109.57(11)	S1	Na1	Na1^{3}	130.17(9)
S2	Cu1	S3^{8}	105.93(13)	S1	Na1	Na1^{1}	49.83(9)
S2	Cu1	S3	109.57(11)	S1^{3}	Na1	Na1^{3}	49.83(9)
S2	Cu1	Na1^{6}	62.53(12)	S1^{3}	Na1	Na1^{1}	130.17(9)
S2	Cu1	Na1^{5}	62.53(12)	S2^{13}	Na1	Cu1^{11}	84.15(13)
S2	Cu1	Na1^{4}	156.23(15)	S2^{9}	Na1	Cu1^{13}	126.05(16)

Table S6. Bond Angles for $NaCuHfS_3$.

Atom	Atom	Atom	Angle/°	Atom	Atom	Atom	Angle/°
S3[3]	Cu1	Hf1	112.96(11)	S2[9]	Na1	Cu1[11]	101.07(17)
S3[3]	Cu1	Hf1[3]	54.09(8)	S2[12]	Na1	Cu1[13]	82.68(14)
S3[8]	Cu1	Hf1	132.71(6)	S2[13]	Na1	Cu1[12]	82.68(14)
S3	Cu1	Hf1[3]	112.96(11)	S2[12]	Na1	Cu1[11]	84.15(13)
S3[8]	Cu1	Hf1[3]	132.71(6)	S2[13]	Na1	Cu1[13]	40.78(8)
S3	Cu1	Hf1	54.10(8)	S2[9]	Na1	Cu1[12]	126.05(16)
S3[3]	Cu1	Cu1[8]	126.24(15)	S2[12]	Na1	Cu1[12]	40.78(8)
S3	Cu1	Cu1[8]	58.40(8)	S2[12]	Na1	S2[9]	143.83(7)
S3[8]	Cu1	Cu1[7]	55.92(9)	S2[13]	Na1	S2[9]	143.83(7)
S3	Cu1	Cu1[7]	126.23(15)	S2[12]	Na1	S2[13]	71.98(14)
S3[3]	Cu1	Cu1[7]	58.40(8)	S2[12]	Na1	Na1[3]	54.01(7)
S3[8]	Cu1	Cu1[8]	55.92(9)	S2[9]	Na1	Na1[3]	90.0
S3[3]	Cu1	S3[8]	114.32(10)	S2[13]	Na1	Na1[3]	125.99(7)
S3[3]	Cu1	S3	103.11(13)	S2[9]	Na1	Na1[1]	90.0
S3	Cu1	S3[8]	114.32(10)	S2[12]	Na1	Na1[1]	125.99(7)
S3[8]	Cu1	Na1[4]	97.84(14)	S2[13]	Na1	Na1[1]	54.01(7)
S3[3]	Cu1	Na1[6]	160.84(9)	S3[11]	Na1	Cu1[11]	43.76(9)
S3	Cu1	Na1[4]	58.83(9)	S3[14]	Na1	Cu1[11]	43.76(9)
S3[3]	Cu1	Na1[5]	96.04(8)	S3[14]	Na1	Cu1[12]	87.53(15)
S3[8]	Cu1	Na1[6]	56.40(11)	S3[11]	Na1	Cu1[12]	43.81(9)
S3[3]	Cu1	Na1[4]	58.83(9)	S3[11]	Na1	Cu1[13]	87.53(15)
S3[8]	Cu1	Na1[5]	56.40(11)	S3[14]	Na1	Cu1[13]	43.81(9)
S3	Cu1	Na1[6]	96.04(8)	S3[14]	Na1	S2[12]	121.46(19)
S3	Cu1	Na1[5]	160.84(9)	S3[11]	Na1	S2[13]	121.46(19)
Na1[4]	Cu1	Na1[6]	135.01(8)	S3[14]	Na1	S2[9]	82.27(15)
Na1[4]	Cu1	Na1[5]	135.01(8)	S3[11]	Na1	S2[12]	77.16(11)
Na1[6]	Cu1	Na1[5]	64.80(11)	S3[11]	Na1	S2[9]	82.27(15)
Hf1[3]	S1	Hf1	90.84(9)	S3[14]	Na1	S2[13]	77.16(11)
Hf1[3]	S1	Hf1[2]	97.12(8)	S3[11]	Na1	S3[14]	78.56(15)
Hf1	S1	Hf1[2]	97.12(8)	S3[11]	Na1	Na1[1]	129.28(8)
Hf1[2]	S1	Na1[1]	102.79(14)	S3[14]	Na1	Na1[1]	50.72(8)
Hf1[3]	S1	Na1	91.05(8)	S3[11]	Na1	Na1[3]	50.72(8)
Hf1[2]	S1	Na1	102.79(14)	S3[14]	Na1	Na1[3]	129.28(8)
Hf1[3]	S1	Na1[1]	159.60(17)	Na1[1]	Na1	Na1[3]	180.0
Hf1	S1	Na1	159.60(17)				

[1]+X,1+Y,+Z; [2]1-X,2-Y,1-Z; [3]+X,-1+Y,+Z; [4]1/2-X,1-Y,-1/2+Z; [5]1/2+X,+Y,1/2-Z; [6]1/2+X,1+Y,1/2-Z; [7]1-X,1-Y,-Z; [8]1-X,2-Y,-Z; [9]1-X,1-Y,1-Z; [10]1/2-X,2-Y,-1/2+Z; [11]1/2-X,1-Y,1/2+Z; [12]-1/2+X,-1+Y,1/2-Z; [13]-1/2+X,+Y,1/2-Z; [14]1/2-X,2-Y,1/2+Z

$RbCuHfS_3$ tables (S7-S11)

Table S7. Crystal data and structure refinement for $RbCuHfS_3$.

Identification code	2465828
Empirical formula	$RbCuHfS_3$
Formula weight	423.68
Temperature/K	173.00(10)
Crystal system	orthorhombic
Space group	Cmcm
a/Å	3.7261(2)
b/Å	14.5594(8)
c/Å	9.7569(5)
α/°	90
β/°	90
γ/°	90
Volume/Å^3	529.31(5)
Z	4
ρ_{calc}g/cm^3	5.317
μ/mm^{-1}	61.348
F(000)	744.0
Crystal size/mm^3	0.073 × 0.057 × 0.022
Radiation	Cu Kα (λ = 1.54184)
2Θ range for data collection/°	15.186 to 147.606
Index ranges	-4 ≤ h ≤ 4, -16 ≤ k ≤ 12, -11 ≤ l ≤ 4
Reflections collected	652
Independent reflections	302 [R_{int} = 0.0340, R_{sigma} = 0.0386]
Data/restraints/parameters	302/0/23
Goodness-of-fit on F^2	1.016
Final R indexes [I>=2σ (I)]	R_1 = 0.0472, wR_2 = 0.1200
Final R indexes [all data]	R_1 = 0.0494, wR_2 = 0.1228
Largest diff. peak/hole / e Å^{-3}	2.74/-1.88

Table S8. Fractional Atomic Coordinates (×10^4) and Equivalent Isotropic Displacement Parameters (Å^2×10^3) for $RbCuHfS_3$. U_{eq} is defined as 1/3 of the trace of the orthogonalised U_{IJ} tensor.

Atom	x	y	z	U(eq)	Wyckoff site	Symmetry
Hf1	10000	5000	5000	14.0(5)	4b	2/m..

Table S8. Fractional Atomic Coordinates (×10^4) and Equivalent Isotropic Displacement Parameters (Å^2×10^3) for $RbCuHfS_3$. U_{eq} is defined as 1/3 of the trace of the orthogonalised U_{IJ} tensor.

Atom	x	y	z	U(eq)	Wyckoff site	Symmetry
Rb1	-5000	7430.3(12)	2500	19.6(5)	4c	m2m
Cu1	5000	4620(2)	2500	17.4(7)	4c	m2m
S1	0	5585(3)	2500	14.2(9)	4c	m2m
S2	5000	3830(2)	4523(3)	13.8(7)	8f	m..

Table S9. Anisotropic Displacement Parameters (Å^2×10^3) for $RbCuHfS_3$. The Anisotropic displacement factor exponent takes the form: $-2\pi^2[h^2a^{*2}U_{11}+2hka^*b^*U_{12}+\ldots]$.

Atom	U_{11}	U_{22}	U_{33}	U_{23}	U_{13}	U_{12}
Hf1	13.2(7)	18.2(7)	10.5(7)	0.2(2)	0	0
Rb1	20.2(11)	19.9(10)	18.5(9)	0	0	0
Cu1	18.7(16)	22.9(18)	10.7(14)	0	0	0
S1	15(2)	18(2)	9(2)	0	0	0
S2	11.8(15)	17.9(15)	11.5(15)	-0.7(11)	0	0

Table S10. Bond Lengths for $RbCuHfS_3$.

Atom	Atom	Length/Å	Atom	Atom	Length/Å
Hf1	Rb1[1]	4.4663(15)	Rb1	Cu1[7]	3.693(3)
Hf1	Rb1[2]	4.4663(15)	Rb1	Cu1[8]	3.693(3)
Hf1	Cu1	3.1188(5)	Rb1	S1[6]	3.269(4)
Hf1	Cu1[3]	3.1187(5)	Rb1	S1	3.269(4)
Hf1	Cu1[4]	3.1187(5)	Rb1	S2[8]	3.394(3)
Hf1	Cu1[5]	3.1187(5)	Rb1	S2[9]	3.435(3)
Hf1	S1[3]	2.5837(15)	Rb1	S2[10]	3.394(3)
Hf1	S1[4]	2.5837(15)	Rb1	S2[11]	3.394(3)
Hf1	S2[3]	2.567(2)	Rb1	S2[7]	3.394(3)
Hf1	S2[5]	2.567(2)	Rb1	S2[12]	3.435(3)
Hf1	S2[4]	2.567(2)	Cu1	S1[4]	2.333(3)
Hf1	S2	2.567(2)	Cu1	S1	2.333(3)
Rb1	Rb1[4]	3.7261(2)	Cu1	S2	2.285(3)
Rb1	Rb1[6]	3.7261(2)	Cu1	S2[13]	2.285(3)

[1]3/2+X,-1/2+Y,+Z; [2]1/2-X,3/2-Y,1-Z; [3]1-X,1-Y,1-Z; [4]1+X,+Y,+Z; [5]2-X,1-Y,1-Z; [6]-1+X,+Y,+Z; [7]-3/2+X,1/2+Y,+Z; [8]-1/2+X,1/2+Y,+Z; [9]-X,1-Y,1-Z; [10]-1/2+X,1/2+Y,1/2-Z; [11]-3/2+X,1/2+Y,1/2-Z; [12]-X,1-Y,-1/2+Z; [13]+X,+Y,1/2-Z

Table S11 Bond Angles for $RbCuHfS_3$.

Atom	Atom	Atom	Angle/°	Atom	Atom	Atom	Angle/°
Rb1[1]	Hf1	Rb1[2]	180.0	S2[10]	Rb1	Cu1[7]	117.45(4)
Cu1[3]	Hf1	Rb1[2]	54.85(5)	S2[9]	Rb1	Cu1[8]	76.03(6)
Cu1[4]	Hf1	Rb1[1]	54.85(5)	S2[7]	Rb1	Cu1[7]	37.32(5)
Cu1[3]	Hf1	Rb1[1]	125.15(5)	S2[10]	Rb1	Cu1[8]	117.45(4)
Cu1[5]	Hf1	Rb1[2]	125.15(5)	S2[7]	Rb1	Cu1[8]	76.03(6)
Cu1[5]	Hf1	Rb1[1]	54.85(5)	S2[11]	Rb1	Cu1[8]	37.32(5)
Cu1	Hf1	Rb1[2]	54.85(5)	S2[11]	Rb1	Cu1[7]	76.03(6)
Cu1[4]	Hf1	Rb1[2]	125.15(5)	S2[8]	Rb1	Cu1[7]	76.03(6)
Cu1	Hf1	Rb1[1]	125.15(5)	S2[7]	Rb1	S2[12]	144.32(5)
Cu1[3]	Hf1	Cu1[5]	180.0	S2[11]	Rb1	S2[12]	80.14(7)
Cu1[5]	Hf1	Cu1	106.636(15)	S2[8]	Rb1	S2[10]	80.14(7)
Cu1[4]	Hf1	Cu1[5]	73.364(15)	S2[8]	Rb1	S2[11]	71.13(10)
Cu1[3]	Hf1	Cu1	73.364(14)	S2[8]	Rb1	S2[12]	144.32(5)
Cu1[4]	Hf1	Cu1[3]	106.636(15)	S2[12]	Rb1	S2[10]	115.45(10)
Cu1[4]	Hf1	Cu1	180.0	S2[11]	Rb1	S2[7]	106.21(11)
S1[5]	Hf1	Rb1[1]	76.15(10)	S2[9]	Rb1	S2[12]	80.14(7)
S1[3]	Hf1	Rb1[2]	76.15(10)	S2[9]	Rb1	S2[7]	71.13(10)
S1[3]	Hf1	Rb1[1]	103.85(10)	S2[8]	Rb1	S2[7]	66.59(6)
S1[5]	Hf1	Rb1[2]	103.85(10)	S2[11]	Rb1	S2[10]	144.32(5)
S1[3]	Hf1	Cu1	47.16(6)	S2[7]	Rb1	S2[10]	80.14(7)
S1[3]	Hf1	Cu1[5]	132.84(6)	S2[11]	Rb1	S2[9]	66.59(6)
S1[5]	Hf1	Cu1	132.84(6)	S2[8]	Rb1	S2[9]	106.21(11)
S1[5]	Hf1	Cu1[3]	132.84(6)	S2[9]	Rb1	S2[10]	144.32(5)
S1[3]	Hf1	Cu1[3]	47.16(6)	Hf1[13]	Cu1	Hf1[6]	102.91(2)
S1[3]	Hf1	Cu1[4]	132.84(6)	Hf1	Cu1	Hf1[13]	159.57(10)
S1[5]	Hf1	Cu1[5]	47.16(6)	Hf1[6]	Cu1	Hf1[14]	159.57(10)
S1[5]	Hf1	Cu1[4]	47.16(6)	Hf1	Cu1	Hf1[14]	102.91(2)
S1[5]	Hf1	S1[3]	180.0	Hf1[13]	Cu1	Hf1[14]	73.364(15)
S2[4]	Hf1	Rb1[2]	130.92(5)	Hf1	Cu1	Hf1[6]	73.364(14)
S2	Hf1	Rb1[1]	130.92(5)	Hf1[13]	Cu1	Rb1[15]	81.47(3)
S2[4]	Hf1	Rb1[1]	49.08(5)	Hf1[14]	Cu1	Rb1[2]	81.47(3)
S2[3]	Hf1	Rb1[2]	49.08(5)	Hf1[6]	Cu1	Rb1[15]	81.47(3)
S2[3]	Hf1	Rb1[1]	130.92(5)	Hf1[14]	Cu1	Rb1[15]	117.03(6)
S2[5]	Hf1	Rb1[1]	49.08(5)	Hf1[13]	Cu1	Rb1[3]	79.79(5)
S2[5]	Hf1	Rb1[2]	130.92(5)	Hf1	Cu1	Rb1[15]	117.03(6)

Table S11 Bond Angles for $RbCuHfS_3$.

Atom	Atom	Atom	Angle/°	Atom	Atom	Atom	Angle/°
S2	Hf1	Rb1^{2}	49.08(5)	Hf1	Cu1	Rb1^{3}	79.79(5)
S2	Hf1	Cu1	46.13(8)	Hf1^{14}	Cu1	Rb1^{3}	79.79(5)
S2^{5}	Hf1	Cu1^{3}	133.87(8)	Hf1^{6}	Cu1	Rb1^{3}	79.79(5)
S2^{3}	Hf1	Cu1^{4}	79.98(7)	Hf1	Cu1	Rb1^{2}	81.47(3)
S2	Hf1	Cu1^{4}	133.87(8)	Hf1^{6}	Cu1	Rb1^{2}	117.03(6)
S2^{3}	Hf1	Cu1^{5}	133.87(8)	Hf1^{13}	Cu1	Rb1^{2}	117.03(6)
S2^{5}	Hf1	Cu1^{5}	46.13(8)	Rb1^{15}	Cu1	Rb1^{3}	149.70(3)
S2^{5}	Hf1	Cu1	79.98(7)	Rb1^{15}	Cu1	Rb1^{2}	60.60(5)
S2^{4}	Hf1	Cu1^{3}	79.98(7)	Rb1^{2}	Cu1	Rb1^{3}	149.70(3)
S2^{3}	Hf1	Cu1^{3}	46.13(8)	S1^{3}	Cu1	Hf1^{13}	111.73(8)
S2^{4}	Hf1	Cu1^{5}	100.02(7)	S1^{3}	Cu1	Hf1^{14}	54.29(3)
S2^{3}	Hf1	Cu1	100.02(7)	S1^{3}	Cu1	Hf1^{6}	111.73(8)
S2^{5}	Hf1	Cu1^{4}	100.02(7)	S1	Cu1	Hf1^{13}	54.29(3)
S2^{4}	Hf1	Cu1	133.87(8)	S1	Cu1	Hf1	111.73(8)
S2^{4}	Hf1	Cu1^{4}	46.13(8)	S1^{3}	Cu1	Hf1	54.29(3)
S2	Hf1	Cu1^{3}	100.02(7)	S1	Cu1	Hf1^{14}	111.73(8)
S2	Hf1	Cu1^{5}	79.98(7)	S1	Cu1	Hf1^{6}	54.29(3)
S2^{5}	Hf1	S1^{3}	87.27(10)	S1^{3}	Cu1	Rb1^{3}	52.98(10)
S2^{3}	Hf1	S1^{3}	92.73(10)	S1	Cu1	Rb1^{3}	52.98(10)
S2^{4}	Hf1	S1^{3}	87.27(10)	S1^{3}	Cu1	Rb1^{15}	157.32(12)
S2	Hf1	S1^{5}	87.27(10)	S1^{3}	Cu1	Rb1^{2}	96.72(10)
S2^{5}	Hf1	S1^{5}	92.73(10)	S1	Cu1	Rb1^{15}	96.72(10)
S2^{3}	Hf1	S1^{5}	87.27(10)	S1	Cu1	Rb1^{2}	157.32(12)
S2	Hf1	S1^{3}	92.73(10)	S1^{3}	Cu1	S1	106.0(2)
S2^{4}	Hf1	S1^{5}	92.73(10)	S2^{16}	Cu1	Hf1^{13}	54.10(5)
S2^{5}	Hf1	S2^{3}	180.0	S2	Cu1	Hf1^{14}	139.90(6)
S2	Hf1	S2^{3}	93.05(9)	S2^{16}	Cu1	Hf1^{14}	54.10(5)
S2^{4}	Hf1	S2^{3}	86.95(9)	S2	Cu1	Hf1	54.10(5)
S2^{4}	Hf1	S2^{5}	93.05(9)	S2	Cu1	Hf1^{13}	139.90(6)
S2	Hf1	S2^{4}	180.0	S2	Cu1	Hf1^{6}	54.10(5)
S2	Hf1	S2^{5}	86.95(9)	S2^{16}	Cu1	Hf1^{6}	139.90(6)
Rb1^{6}	Rb1	Rb1^{3}	180.0	S2^{16}	Cu1	Hf1	139.90(6)
Cu1^{7}	Rb1	Rb1^{3}	120.30(3)	S2	Cu1	Rb1^{15}	64.22(8)
Cu1^{8}	Rb1	Rb1^{3}	59.70(3)	S2^{16}	Cu1	Rb1^{3}	120.25(10)
Cu1^{7}	Rb1	Rb1^{6}	59.70(3)	S2^{16}	Cu1	Rb1^{15}	64.22(8)
Cu1^{8}	Rb1	Rb1^{6}	120.30(3)	S2	Cu1	Rb1^{2}	64.22(8)
Cu1^{8}	Rb1	Cu1^{7}	60.60(5)	S2^{16}	Cu1	Rb1^{2}	64.22(8)

Table S11 Bond Angles for RbCuHfS_3.

Atom	Atom	Atom	Angle/°	Atom	Atom	Atom	Angle/°
S1	Rb1	Rb1^{6}	124.74(5)	S2	Cu1	Rb1^{3}	120.25(10)
S1^{6}	Rb1	Rb1^{6}	55.26(5)	S2^{16}	Cu1	S1^{3}	107.66(5)
S1^{6}	Rb1	Rb1^{3}	124.74(5)	S2^{16}	Cu1	S1	107.66(5)
S1	Rb1	Rb1^{3}	55.26(5)	S2	Cu1	S1	107.66(5)
S1^{6}	Rb1	Cu1^{8}	175.56(6)	S2	Cu1	S1^{3}	107.66(5)
S1	Rb1	Cu1^{8}	114.96(5)	S2	Cu1	S2^{16}	119.51(19)
S1	Rb1	Cu1^{7}	175.56(6)	Hf1^{13}	S1	Hf1^{6}	141.50(19)
S1^{6}	Rb1	Cu1^{7}	114.96(5)	Hf1^{13}	S1	Rb1	105.72(7)
S1^{6}	Rb1	S1	69.48(10)	Hf1^{13}	S1	Rb1^{3}	105.72(7)
S1	Rb1	S2^{9}	143.72(6)	Hf1^{6}	S1	Rb1	105.72(7)
S1	Rb1	S2^{7}	143.72(6)	Hf1^{6}	S1	Rb1^{3}	105.72(7)
S1^{6}	Rb1	S2^{7}	100.40(5)	Rb1^{3}	S1	Rb1	69.48(10)
S1^{6}	Rb1	S2^{10}	63.97(5)	Cu1	S1	Hf1^{13}	78.55(8)
S1	Rb1	S2^{10}	63.97(5)	Cu1^{6}	S1	Hf1^{6}	78.55(8)
S1^{6}	Rb1	S2^{9}	100.40(5)	Cu1	S1	Hf1^{6}	78.55(8)
S1	Rb1	S2^{11}	100.40(5)	Cu1^{6}	S1	Hf1^{13}	78.55(8)
S1^{6}	Rb1	S2^{12}	63.97(5)	Cu1^{6}	S1	Rb1^{3}	161.76(15)
S1	Rb1	S2^{12}	63.97(5)	Cu1	S1	Rb1	161.76(15)
S1^{6}	Rb1	S2^{11}	143.72(6)	Cu1	S1	Rb1^{3}	92.28(7)
S1^{6}	Rb1	S2^{8}	143.72(6)	Cu1^{6}	S1	Rb1	92.28(7)
S1	Rb1	S2^{8}	100.40(5)	Cu1^{6}	S1	Cu1	106.0(2)
S2^{11}	Rb1	Rb1^{6}	123.30(3)	Hf1	S2	Hf1^{6}	93.05(9)
S2^{11}	Rb1	Rb1^{3}	56.70(3)	Hf1^{6}	S2	Rb1^{15}	96.06(4)
S2^{9}	Rb1	Rb1^{6}	56.70(3)	Hf1	S2	Rb1^{2}	96.06(4)
S2^{8}	Rb1	Rb1^{3}	56.70(3)	Hf1^{6}	S2	Rb1^{2}	154.46(11)
S2^{9}	Rb1	Rb1^{3}	123.30(3)	Hf1	S2	Rb1^{15}	154.46(11)
S2^{12}	Rb1	Rb1^{6}	90.0	Hf1	S2	Rb1^{10}	101.60(8)
S2^{12}	Rb1	Rb1^{3}	90.0	Hf1^{6}	S2	Rb1^{10}	101.60(8)
S2^{10}	Rb1	Rb1^{3}	90.0	Rb1^{15}	S2	Rb1^{10}	99.86(7)
S2^{8}	Rb1	Rb1^{6}	123.30(3)	Rb1^{15}	S2	Rb1^{2}	66.59(6)
S2^{7}	Rb1	Rb1^{6}	56.70(3)	Rb1^{2}	S2	Rb1^{10}	99.86(7)
S2^{7}	Rb1	Rb1^{3}	123.30(3)	Cu1	S2	Hf1^{6}	79.76(9)
S2^{10}	Rb1	Rb1^{6}	90.0	Cu1	S2	Hf1	79.76(9)
S2^{12}	Rb1	Cu1^{8}	117.45(4)	Cu1	S2	Rb1^{15}	78.46(9)
S2^{9}	Rb1	Cu1^{7}	37.32(5)	Cu1	S2	Rb1^{10}	177.97(14)
S2^{12}	Rb1	Cu1^{7}	117.45(4)	Cu1	S2	Rb1^{2}	78.46(9)
S2^{8}	Rb1	Cu1^{8}	37.32(5)				

[1]1/2-X,3/2-Y,1-Z; [2]3/2+X,-1/2+Y,+Z; [3]1+X,+Y,+Z; [4]2-X,1-Y,1-Z; [5]1-X,1-Y,1-Z; [6]-1+X,+Y,+Z; [7]-3/2+X,1/2+Y,+Z; [8]-1/2+X,1/2+Y,+Z; [9]-3/2+X,1/2+Y,1/2-Z; [10]-X,1-Y,1-Z; [11]-1/2+X,1/2+Y,1/2-Z; [12]-X,1-Y,-1/2+Z; [13]1-X,1-Y,-1/2+Z; [14]2-X,1-Y,-1/2+Z; [15]1/2+X,-1/2+Y,+Z; [16]+X,+Y,1/2-Z

$CsCuHfS_3$ (S12-S16)

Table 12. Crystal data and structure refinement for $CsCuHfS_3$.

Identification code	2465833
Empirical formula	$CsCuHfS_3$
Formula weight	471.12
Temperature/K	173.00(10)
Crystal system	orthorhombic
Space group	Cmcm
a/Å	3.7443(2)
b/Å	15.2863(9)
c/Å	9.7795(5)
α/°	90
β/°	90
γ/°	90
Volume/Å^3	559.74(5)
Z	4
ρ_{calc}g/cm^3	5.591
μ/mm^{-1}	97.459
F(000)	816.0
Crystal size/mm^3	0.08 × 0.01 × 0.005
Radiation	Cu Kα (λ = 1.54184)
2Θ range for data collection/°	11.578 to 148.25
Index ranges	-3 ≤ h ≤ 4, -17 ≤ k ≤ 18, -11 ≤ l ≤ 11
Reflections collected	796
Independent reflections	337 [R_{int} = 0.0382, R_{sigma} = 0.0356]
Data/restraints/parameters	337/0/24
Goodness-of-fit on F^2	1.156
Final R indexes [I>=2σ (I)]	R_1 = 0.0264, wR_2 = 0.0750
Final R indexes [all data]	R_1 = 0.0268, wR_2 = 0.0752
Largest diff. peak/hole / e Å^{-3}	1.25/-2.01

Table S13. Fractional Atomic Coordinates (×10^4) and Equivalent Isotropic Displacement Parameters (Å^2×10^3) for $CsCuHfS_3$. U_{eq} is defined as 1/3 of the trace of the orthogonalised U_{IJ} tensor.

Atom	x	y	z	U(eq)	Wyckoff site	Symmetry
Hf1	10000	5000	5000	6.2(3)	4b	2/m..
Cs1	-5000	7424.0(5)	2500	10.4(3)	4c	m2m

Table S13. Fractional Atomic Coordinates (×10^4) and Equivalent Isotropic Displacement Parameters (Å^2×10^3) for $CsCuHfS_3$. U_{eq} is defined as 1/3 of the trace of the orthogonalised U_{IJ} tensor.

Atom	x	y	z	U(eq)	Wyckoff site	Symmetry
Cu1	5000	4630.5(14)	2500	9.3(5)	4c	m2m
S2	5000	3886.3(14)	4531(2)	6.9(5)	8f	m..
S1	0	5542(2)	2500	6.5(6)	4c	m2m

Table S14. Anisotropic Displacement Parameters (Å^2×10^3) for $CsCuHfS_3$. The Anisotropic displacement factor exponent takes the form: $-2\pi^2[h^2a^{*2}U_{11}+2hka^*b^*U_{12}+\ldots]$.

Atom	U_{11}	U_{22}	U_{33}	U_{23}	U_{13}	U_{12}
Hf1	7.3(5)	6.5(4)	4.6(5)	0.0(2)	0	0
Cs1	11.3(5)	8.8(5)	11.3(5)	0	0	0
Cu1	11.3(10)	10.0(10)	6.7(10)	0	0	0
S2	10.7(10)	5.0(10)	5.2(10)	0.1(8)	0	0
S1	10.0(15)	5.7(14)	3.8(14)	0	0	0

Table S15. Bond Lengths for $CsCuHfS_3$.

Atom	Atom	Length/Å	Atom	Atom	Length/Å
Hf1	Cs1[1]	4.6350(7)	Cs1	Cu1[7]	3.858(2)
Hf1	Cs1[2]	4.6350(7)	Cs1	Cu1[8]	3.858(2)
Hf1	Cu1[3]	3.1307(4)	Cs1	S2[9]	3.527(2)
Hf1	Cu1[4]	3.1307(4)	Cs1	S2[10]	3.527(2)
Hf1	Cu1	3.1307(4)	Cs1	S2[8]	3.528(2)
Hf1	Cu1[5]	3.1307(4)	Cs1	S2[11]	3.528(2)
Hf1	S2	2.5717(15)	Cs1	S2[12]	3.528(2)
Hf1	S2[5]	2.5717(15)	Cs1	S2[7]	3.528(2)
Hf1	S2[3]	2.5717(15)	Cs1	S1[6]	3.432(3)
Hf1	S2[4]	2.5717(15)	Cs1	S1	3.432(3)
Hf1	S1[4]	2.5815(10)	Cu1	S2	2.289(2)
Hf1	S1[5]	2.5815(10)	Cu1	S2[13]	2.289(2)
Cs1	Cs1[6]	3.7443(2)	Cu1	S1[5]	2.334(2)
Cs1	Cs1[5]	3.7443(2)	Cu1	S1	2.334(2)

[1]3/2+X,-1/2+Y,+Z; [2]1/2-X,3/2-Y,1-Z; [3]2-X,1-Y,1-Z; [4]1-X,1-Y,1-Z; [5]1+X,+Y,+Z; [6]-1+X,+Y,+Z; [7]-1/2+X,1/2+Y,+Z; [8]-3/2+X,1/2+Y,+Z; [9]-X,1-Y,-1/2+Z; [10]-X,1-Y,1-Z; [11]-1/2+X,1/2+Y,1/2-Z; [12]-3/2+X,1/2+Y,1/2-Z; [13]+X,+Y,1/2-Z

Table S16. Bond Angles for $CsCuHfS_3$.

Atom	Atom	Atom	Angle/˚	Atom	Atom	Atom	Angle/˚
Cs1^{1}	Hf1	Cs1^{2}	180.0	S2^{7}	Cs1	S2^{11}	68.53(7)
Cu1^{3}	Hf1	Cs1^{2}	55.58(4)	S2^{12}	Cs1	S2^{11}	145.41(3)
Cu1	Hf1	Cs1^{1}	124.42(4)	S1^{6}	Cs1	Cs1^{3}	123.06(3)
Cu1^{3}	Hf1	Cs1^{1}	124.42(4)	S1^{6}	Cs1	Cs1^{6}	56.95(3)
Cu1^{4}	Hf1	Cs1^{1}	55.58(4)	S1	Cs1	Cs1^{3}	56.94(3)
Cu1	Hf1	Cs1^{2}	55.58(4)	S1	Cs1	Cs1^{6}	123.06(3)
Cu1^{5}	Hf1	Cs1^{1}	55.58(4)	S1^{6}	Cs1	Cu1^{8}	117.91(3)
Cu1^{5}	Hf1	Cs1^{2}	124.42(4)	S1	Cs1	Cu1^{8}	175.98(4)
Cu1^{4}	Hf1	Cs1^{2}	124.42(4)	S1^{6}	Cs1	Cu1^{7}	175.98(4)
Cu1^{5}	Hf1	Cu1^{3}	106.547(12)	S1	Cs1	Cu1^{7}	117.91(3)
Cu1^{3}	Hf1	Cu1	73.453(11)	S1^{6}	Cs1	S2^{10}	61.58(3)
Cu1^{5}	Hf1	Cu1	180.0	S1	Cs1	S2^{9}	145.13(4)
Cu1^{5}	Hf1	Cu1^{4}	73.453(12)	S1	Cs1	S2^{8}	145.13(4)
Cu1^{3}	Hf1	Cu1^{4}	180.0	S1	Cs1	S2^{7}	103.98(4)
Cu1^{4}	Hf1	Cu1	106.547(12)	S1	Cs1	S2^{10}	61.58(3)
S2^{4}	Hf1	Cs1^{1}	48.97(4)	S1	Cs1	S2^{12}	61.58(3)
S2^{3}	Hf1	Cs1^{1}	131.03(4)	S1^{6}	Cs1	S2^{9}	103.98(4)
S2^{3}	Hf1	Cs1^{2}	48.97(4)	S1^{6}	Cs1	S2^{7}	145.13(4)
S2	Hf1	Cs1^{1}	131.03(4)	S1	Cs1	S2^{11}	103.98(4)
S2^{5}	Hf1	Cs1^{2}	131.03(4)	S1^{6}	Cs1	S2^{12}	61.58(3)
S2^{4}	Hf1	Cs1^{2}	131.03(4)	S1^{6}	Cs1	S2^{8}	103.98(4)
S2^{5}	Hf1	Cs1^{1}	48.97(4)	S1^{6}	Cs1	S2^{11}	145.13(4)
S2	Hf1	Cs1^{2}	48.97(4)	S1	Cs1	S1^{6}	66.11(6)
S2^{3}	Hf1	Cu1^{3}	46.05(6)	Hf1	Cu1	Hf1^{13}	159.21(8)
S2^{3}	Hf1	Cu1^{5}	79.82(5)	Hf1	Cu1	Hf1^{6}	73.453(11)
S2	Hf1	Cu1^{4}	79.82(5)	Hf1^{13}	Cu1	Hf1^{6}	102.692(18)
S2^{5}	Hf1	Cu1^{4}	100.18(5)	Hf1	Cu1	Hf1^{14}	102.692(18)
S2	Hf1	Cu1^{5}	133.95(6)	Hf1^{6}	Cu1	Hf1^{14}	159.21(8)
S2^{4}	Hf1	Cu1^{4}	46.05(6)	Hf1^{13}	Cu1	Hf1^{14}	73.453(11)
S2	Hf1	Cu1	46.05(6)	Hf1^{6}	Cu1	Cs1^{15}	82.39(3)
S2^{3}	Hf1	Cu1^{4}	133.95(6)	Hf1^{6}	Cu1	Cs1^{2}	116.61(4)
S2^{4}	Hf1	Cu1	79.82(5)	Hf1^{14}	Cu1	Cs1^{15}	116.61(4)
S2^{5}	Hf1	Cu1	133.95(6)	Hf1	Cu1	Cs1^{2}	82.39(3)
S2^{4}	Hf1	Cu1^{3}	133.95(6)	Hf1	Cu1	Cs1^{3}	79.61(4)
S2^{5}	Hf1	Cu1^{5}	46.05(6)	Hf1^{14}	Cu1	Cs1^{2}	82.39(3)
S2^{4}	Hf1	Cu1^{5}	100.18(5)	Hf1^{14}	Cu1	Cs1^{3}	79.61(4)
S2^{3}	Hf1	Cu1	100.18(5)	Hf1	Cu1	Cs1^{15}	116.61(4)

Table S16. Bond Angles for $CsCuHfS_3$.

Atom	Atom	Atom	Angle/°	Atom	Atom	Atom	Angle/°
S2	Hf1	Cu1^{3}	100.18(5)	Hf1^{13}	Cu1	Cs1^{15}	82.39(3)
S2^{5}	Hf1	Cu1^{3}	79.82(5)	Hf1^{6}	Cu1	Cs1^{3}	79.61(4)
S2	Hf1	S2^{3}	93.43(7)	Hf1^{13}	Cu1	Cs1^{2}	116.61(4)
S2^{5}	Hf1	S2^{3}	86.57(7)	Hf1^{13}	Cu1	Cs1^{3}	79.61(4)
S2^{4}	Hf1	S2^{3}	180.0	Cs1^{15}	Cu1	Cs1^{3}	150.968(17)
S2	Hf1	S2^{4}	86.57(7)	Cs1^{15}	Cu1	Cs1^{2}	58.06(3)
S2^{5}	Hf1	S2^{4}	93.43(7)	Cs1^{2}	Cu1	Cs1^{3}	150.968(17)
S2	Hf1	S2^{5}	180.0	S2^{16}	Cu1	Hf1^{13}	53.99(4)
S2	Hf1	S1^{4}	87.50(7)	S2	Cu1	Hf1	53.99(4)
S2^{5}	Hf1	S1^{3}	87.50(7)	S2^{16}	Cu1	Hf1^{6}	140.11(4)
S2^{3}	Hf1	S1^{3}	92.50(7)	S2^{16}	Cu1	Hf1^{14}	53.99(4)
S2	Hf1	S1^{3}	92.50(7)	S2	Cu1	Hf1^{6}	53.99(4)
S2^{4}	Hf1	S1^{3}	87.50(7)	S2	Cu1	Hf1^{13}	140.11(4)
S2^{4}	Hf1	S1^{4}	92.50(7)	S2	Cu1	Hf1^{14}	140.11(4)
S2^{5}	Hf1	S1^{4}	92.50(7)	S2^{16}	Cu1	Hf1	140.11(4)
S2^{3}	Hf1	S1^{4}	87.50(7)	S2^{16}	Cu1	Cs1^{3}	119.80(7)
S1^{4}	Hf1	Cs1^{1}	76.89(7)	S2	Cu1	Cs1^{3}	119.80(7)
S1^{3}	Hf1	Cs1^{2}	76.89(7)	S2^{16}	Cu1	Cs1^{15}	64.24(6)
S1^{4}	Hf1	Cs1^{2}	103.11(7)	S2	Cu1	Cs1^{15}	64.24(6)
S1^{3}	Hf1	Cs1^{1}	103.11(7)	S2	Cu1	Cs1^{2}	64.24(6)
S1^{4}	Hf1	Cu1	132.98(5)	S2^{16}	Cu1	Cs1^{2}	64.24(6)
S1^{3}	Hf1	Cu1^{3}	47.02(4)	S2	Cu1	S2^{16}	120.40(14)
S1^{3}	Hf1	Cu1^{4}	132.98(4)	S2	Cu1	S1^{3}	107.26(4)
S1^{4}	Hf1	Cu1^{5}	47.02(4)	S2^{16}	Cu1	S1^{3}	107.26(4)
S1^{3}	Hf1	Cu1	47.02(4)	S2	Cu1	S1	107.26(4)
S1^{3}	Hf1	Cu1^{5}	132.98(5)	S2^{16}	Cu1	S1	107.26(4)
S1^{4}	Hf1	Cu1^{3}	132.98(4)	S1	Cu1	Hf1^{14}	111.84(6)
S1^{4}	Hf1	Cu1^{4}	47.02(4)	S1^{3}	Cu1	Hf1	54.03(2)
S1^{4}	Hf1	S1^{3}	180.0	S1	Cu1	Hf1^{13}	54.03(2)
Cs1^{6}	Cs1	Cs1^{3}	180.0	S1^{3}	Cu1	Hf1^{14}	54.03(2)
Cs1^{3}	Cs1	Cu1^{7}	60.968(17)	S1^{3}	Cu1	Hf1^{6}	111.84(6)
Cs1^{3}	Cs1	Cu1^{8}	119.032(17)	S1	Cu1	Hf1	111.84(6)
Cs1^{6}	Cs1	Cu1^{7}	119.032(17)	S1^{3}	Cu1	Hf1^{13}	111.84(6)
Cs1^{6}	Cs1	Cu1^{8}	60.968(17)	S1	Cu1	Hf1^{6}	54.03(2)
Cu1^{7}	Cs1	Cu1^{8}	58.06(3)	S1	Cu1	Cs1^{3}	53.34(7)
S2^{9}	Cs1	Cs1^{3}	122.05(2)	S1	Cu1	Cs1^{2}	155.69(8)
S2^{10}	Cs1	Cs1^{6}	90.0	S1^{3}	Cu1	Cs1^{3}	53.34(7)

Table S16. Bond Angles for $CsCuHfS_3$.

Atom	Atom	Atom	Angle/°	Atom	Atom	Atom	Angle/°
S2^{10}	Cs1	Cs1^{3}	90.0	S1^{3}	Cu1	Cs1^{2}	97.63(7)
S2^{7}	Cs1	Cs1^{6}	122.05(2)	S1	Cu1	Cs1^{15}	97.63(7)
S2^{7}	Cs1	Cs1^{3}	57.95(2)	S1^{3}	Cu1	Cs1^{15}	155.69(8)
S2^{11}	Cs1	Cs1^{6}	122.05(2)	S1^{3}	Cu1	S1	106.68(15)
S2^{8}	Cs1	Cs1^{3}	122.05(2)	Hf1	S2	Hf1^{6}	93.43(7)
S2^{8}	Cs1	Cs1^{6}	57.95(2)	Hf1^{6}	S2	Cs1^{2}	154.97(8)
S2^{12}	Cs1	Cs1^{3}	90.0	Hf1^{6}	S2	Cs1^{15}	97.67(3)
S2^{9}	Cs1	Cs1^{6}	57.95(2)	Hf1	S2	Cs1^{12}	103.25(6)
S2^{12}	Cs1	Cs1^{6}	90.0	Hf1	S2	Cs1^{2}	97.67(3)
S2^{11}	Cs1	Cs1^{3}	57.95(2)	Hf1^{6}	S2	Cs1^{12}	103.25(6)
S2^{9}	Cs1	Cu1^{8}	35.76(4)	Hf1	S2	Cs1^{15}	154.97(8)
S2^{8}	Cs1	Cu1^{8}	35.76(4)	Cs1^{12}	S2	Cs1^{15}	95.95(5)
S2^{11}	Cs1	Cu1^{8}	72.76(3)	Cs1^{15}	S2	Cs1^{2}	64.10(4)
S2^{8}	Cs1	Cu1^{7}	72.76(3)	Cs1^{12}	S2	Cs1^{2}	95.95(5)
S2^{9}	Cs1	Cu1^{7}	72.76(3)	Cu1	S2	Hf1	79.96(6)
S2^{7}	Cs1	Cu1^{7}	35.76(4)	Cu1	S2	Hf1^{6}	79.96(6)
S2^{12}	Cs1	Cu1^{8}	119.77(3)	Cu1	S2	Cs1^{12}	175.20(10)
S2^{7}	Cs1	Cu1^{8}	72.76(3)	Cu1	S2	Cs1^{2}	80.00(7)
S2^{11}	Cs1	Cu1^{7}	35.76(4)	Cu1	S2	Cs1^{15}	80.00(7)
S2^{12}	Cs1	Cu1^{7}	119.77(3)	Hf1^{13}	S1	Hf1^{6}	142.56(13)
S2^{10}	Cs1	Cu1^{8}	119.77(3)	Hf1^{6}	S1	Cs1^{3}	105.61(5)
S2^{10}	Cs1	Cu1^{7}	119.77(3)	Hf1^{13}	S1	Cs1^{3}	105.61(5)
S2^{10}	Cs1	S2^{8}	145.41(3)	Hf1^{6}	S1	Cs1	105.61(5)
S2^{7}	Cs1	S2^{8}	64.10(4)	Hf1^{13}	S1	Cs1	105.61(5)
S2^{8}	Cs1	S2^{11}	101.37(7)	Cs1	S1	Cs1^{3}	66.11(6)
S2^{7}	Cs1	S2^{9}	101.37(7)	Cu1	S1	Hf1^{13}	78.95(6)
S2^{10}	Cs1	S2^{9}	84.05(5)	Cu1	S1	Hf1^{6}	78.95(6)
S2^{12}	Cs1	S2^{9}	145.41(3)	Cu1^{6}	S1	Hf1^{13}	78.95(6)
S2^{10}	Cs1	S2^{11}	84.05(5)	Cu1^{6}	S1	Hf1^{6}	78.95(6)
S2^{11}	Cs1	S2^{9}	64.10(4)	Cu1^{6}	S1	Cs1^{3}	159.71(10)
S2^{10}	Cs1	S2^{12}	110.80(7)	Cu1^{6}	S1	Cs1	93.60(5)
S2^{8}	Cs1	S2^{9}	68.53(7)	Cu1	S1	Cs1^{3}	93.60(5)
S2^{10}	Cs1	S2^{7}	145.41(3)	Cu1	S1	Cs1	159.72(10)
S2^{12}	Cs1	S2^{8}	84.05(5)	Cu1^{6}	S1	Cu1	106.68(15)
S2^{12}	Cs1	S2^{7}	84.05(5)				

^{1}1/2-X,3/2-Y,1-Z; ^{2}3/2+X,-1/2+Y,+Z; ^{3}1+X,+Y,+Z; ^{4}1-X,1-Y,1-Z; ^{5}2-X,1-Y,1-Z; ^{6}-1+X,+Y,+Z; ^{7}-1/2+X,1/2+Y,+Z; ^{8}-3/2+X,1/2+Y,+Z; ^{9}-3/2+X,1/2+Y,1/2-Z; ^{10}-X,1-Y,-1/2+Z; ^{11}-1/2+X,1/2+Y,1/2-Z; ^{12}-X,1-Y,1-Z; ^{13}1-X,1-Y,-1/2+Z; ^{14}2-X,1-Y,-1/2+Z; ^{15}1/2+X,-1/2+Y,+Z; ^{16}+X,+Y,1/2-Z

$KCuHfSe_3$ (S17-S21)

Table S17. Crystal data and structure refinement for $KCuHfSe_3$.

Identification code	2465836
Empirical formula	$KCuHfSe_3$
Formula weight	518.01
Temperature/K	173.15
Crystal system	monoclinic
Space group	$P2_1/m$
a/Å	8.5861(4)
b/Å	3.87550(10)
c/Å	9.4339(4)
α/°	90
β/°	110.706(5)
γ/°	90
Volume/Å^3	293.64(2)
Z	2
ρ_{calc}g/cm^3	5.859
μ/mm^{-1}	62.914
F(000)	444.0
Crystal size/mm^3	0.5 × 0.04 × 0.04
Radiation	CuKα (λ = 1.54184)
2Θ range for data collection/°	10.024 to 148.596
Index ranges	-9 ≤ h ≤ 10, -3 ≤ k ≤ 4, -10 ≤ l ≤ 11
Reflections collected	1227
Independent reflections	654 [R_{int} = 0.0242, R_{sigma} = 0.0212]
Data/restraints/parameters	654/0/38
Goodness-of-fit on F^2	1.116
Final R indexes [I>=2σ (I)]	R_1 = 0.0446, wR_2 = 0.1256
Final R indexes [all data]	R_1 = 0.0448, wR_2 = 0.1258
Largest diff. peak/hole / e Å^{-3}	2.63/-2.59

Table S18. Fractional Atomic Coordinates (×10^4) and Equivalent Isotropic Displacement Parameters (Å^2×10^3) for $KCuHfSe_3$. U_{eq} is defined as 1/3 of the trace of the orthogonalised U_{IJ} tensor.

Atom	x	y	z	U(eq)	Wyckoff site	Symmetry
Hf1	3107.8(6)	12500	8358.7(6)	13.3(4)	2e	m
Se2	1111.4(16)	7500	8661.3(15)	14.3(4)	2e	m

Table S18. Fractional Atomic Coordinates (×10^4) and Equivalent Isotropic Displacement Parameters (Å^2×10^3) for $KCuHfSe_3$. U_{eq} is defined as 1/3 of the trace of the orthogonalised U_{IJ} tensor.

Atom	x	y	z	U(eq)	Wyckoff site	Symmetry
Se3	2293.8(16)	12500	5444.2(14)	14.5(4)	2e	m
Se1	5375.8(16)	7500	8536.2(14)	12.8(4)	2e	m
Cu1	812(2)	7500	5992(2)	17.7(5)	2e	m
K1	7725(4)	2500	7380(3)	20.1(6)	2e	m

Table S19. Anisotropic Displacement Parameters (Å^2×10^3) for $KCuHfSe_3$. The Anisotropic displacement factor exponent takes the form: $-2\pi^2[h^2a^{*2}U_{11}+2hka^*b^*U_{12}+...]$.

Atom	U_{11}	U_{22}	U_{33}	U_{23}	U_{13}	U_{12}
Hf1	12.1(5)	10.8(6)	14.2(5)	0	1.4(3)	0
Se2	13.7(8)	11.3(8)	16.3(7)	0	3.5(6)	0
Se3	13.0(7)	13.2(8)	14.5(7)	0	1.4(6)	0
Se1	11.7(7)	10.8(8)	13.9(7)	0	1.9(5)	0
Cu1	17.3(10)	15.5(10)	17.6(9)	0	3.0(8)	0
K1	22.1(14)	14.8(13)	25.3(14)	0	10.6(11)	0

Table S20. Bond Lengths for $KCuHfSe_3$.

Atom	Atom	Length/Å	Atom	Atom	Length/Å
Hf1	Se2	2.6685(9)	Se3	Cu1^1	2.4710(14)
Hf1	Se2^1	2.6685(9)	Se3	Cu1^6	2.528(2)
Hf1	Se3	2.5864(14)	Se3	K1^7	3.290(2)
Hf1	Se1	2.7101(9)	Se3	K1^8	3.290(2)
Hf1	Se1^2	2.7530(13)	Se1	K1^1	3.251(2)
Hf1	Se1^1	2.7101(9)	Se1	K1	3.251(2)
Hf1	Cu1	3.0843(16)	Cu1	Cu1^9	2.712(3)
Hf1	Cu1^1	3.0843(15)	Cu1	Cu1^6	2.712(3)
Se2	Cu1	2.439(2)	Cu1	K1^7	3.811(3)
Se2	K1^3	3.345(3)	Cu1	K1^3	3.866(3)
Se2	K1^4	3.345(3)	Cu1	K1^4	3.866(3)
Se2	K1^5	3.508(3)	K1	K1^1	3.87550(10)
Se3	Cu1	2.4710(14)	K1	K1^{10}	3.87550(10)

^{1}+X,1+Y,+Z; ^{2}1-X,2-Y,2-Z; ^{3}-1+X,+Y,+Z; ^{4}-1+X,1+Y,+Z; ^{5}1-X,1-Y,2-Z; ^{6}-X,2-Y,1-Z; ^{7}1-X,1-Y,1-Z; ^{8}1-X,2-Y,1-Z; ^{9}-X,1-Y,1-Z; ^{10}+X,-1+Y,+Z

Table S21. Bond Angles for $KCuHfSe_3$.

Atom	Atom	Atom	Angle/°	Atom	Atom	Atom	Angle/°
Se2	Hf1	Se2^{1}	93.13(4)	Se3	Cu1	Cu1^{10}	126.37(10)
Se2^{1}	Hf1	Se1^{1}	87.07(3)	Se3^{3}	Cu1	Cu1^{9}	126.36(10)
Se2	Hf1	Se1^{2}	88.06(4)	Se3^{9}	Cu1	Cu1^{10}	56.13(7)
Se2^{1}	Hf1	Se1^{2}	88.06(4)	Se3^{3}	Cu1	Cu1^{10}	58.16(5)
Se2^{1}	Hf1	Se1	170.87(5)	Se3^{9}	Cu1	Cu1^{9}	56.13(7)
Se2	Hf1	Se1	87.07(3)	Se3^{3}	Cu1	K1^{8}	58.62(4)
Se2	Hf1	Se1^{1}	170.87(5)	Se3^{9}	Cu1	K1^{8}	98.58(8)
Se2	Hf1	Cu1	49.53(4)	Se3	Cu1	K1^{6}	158.31(5)
Se2	Hf1	Cu1^{1}	105.27(4)	Se3	Cu1	K1^{5}	98.23(4)
Se2^{1}	Hf1	Cu1	105.27(4)	Se3^{3}	Cu1	K1^{6}	98.23(4)
Se2^{1}	Hf1	Cu1^{1}	49.53(4)	Se3	Cu1	K1^{8}	58.62(4)
Se3	Hf1	Se2^{1}	99.87(4)	Se3^{9}	Cu1	K1^{6}	57.45(6)
Se3	Hf1	Se2	99.87(4)	Se3^{3}	Cu1	K1^{5}	158.31(5)
Se3	Hf1	Se1^{1}	89.08(4)	Se3^{9}	Cu1	K1^{5}	57.45(6)
Se3	Hf1	Se1	89.08(4)	Cu1^{10}	Cu1	Hf1^{3}	95.26(4)
Se3	Hf1	Se1^{2}	168.39(5)	Cu1^{9}	Cu1	Hf1	95.26(4)
Se3	Hf1	Cu1	50.74(3)	Cu1^{10}	Cu1	Hf1	171.65(9)
Se3	Hf1	Cu1^{1}	50.74(3)	Cu1^{9}	Cu1	Hf1^{3}	171.65(9)
Se1^{1}	Hf1	Se1^{2}	82.82(4)	Cu1^{10}	Cu1	Cu1^{9}	91.19(11)
Se1	Hf1	Se1^{1}	91.29(4)	Cu1^{10}	Cu1	K1^{5}	110.18(10)
Se1	Hf1	Se1^{2}	82.82(4)	Cu1^{9}	Cu1	K1^{5}	68.22(7)
Se1^{2}	Hf1	Cu1	135.31(3)	Cu1^{9}	Cu1	K1^{6}	110.18(10)
Se1	Hf1	Cu1^{1}	139.05(5)	Cu1^{9}	Cu1	K1^{8}	70.41(8)
Se1	Hf1	Cu1	81.77(4)	Cu1^{10}	Cu1	K1^{6}	68.22(7)
Se1^{1}	Hf1	Cu1^{1}	81.77(4)	Cu1^{10}	Cu1	K1^{8}	70.41(8)
Se1^{2}	Hf1	Cu1^{1}	135.31(3)	K1^{8}	Cu1	K1^{5}	138.63(4)
Se1^{1}	Hf1	Cu1	139.05(5)	K1^{8}	Cu1	K1^{6}	138.63(4)
Cu1^{1}	Hf1	Cu1	77.84(5)	K1^{6}	Cu1	K1^{5}	60.16(5)
Hf1	Se2	Hf1^{3}	93.13(4)	Se2^{11}	K1	Se2^{4}	74.41(6)
Hf1^{3}	Se2	K1^{4}	99.32(5)	Se2^{12}	K1	Se2^{11}	70.81(6)
Hf1	Se2	K1^{4}	99.32(5)	Se2^{12}	K1	Se2^{4}	74.41(6)
Hf1	Se2	K1^{5}	92.86(3)	Se2^{12}	K1	Cu1^{11}	78.48(6)
Hf1	Se2	K1^{6}	153.04(6)	Se2^{11}	K1	Cu1^{11}	38.69(4)
Hf1^{3}	Se2	K1^{6}	92.86(3)	Se2^{12}	K1	Cu1^{8}	78.50(6)
Hf1^{3}	Se2	K1^{5}	153.04(6)	Se2^{11}	K1	Cu1^{8}	78.50(6)
Cu1	Se2	Hf1^{3}	74.14(4)	Se2^{4}	K1	Cu1^{11}	112.90(7)
Cu1	Se2	Hf1	74.14(4)	Se2^{4}	K1	Cu1^{12}	112.90(7)

Table S21. Bond Angles for $KCuHfSe_3$.

Atom	Atom	Atom	Angle/°	Atom	Atom	Atom	Angle/°
Cu1	Se2	K1^{6}	82.30(7)	Se2^{11}	K1	Cu1^{12}	78.48(6)
Cu1	Se2	K1^{4}	170.20(8)	Se2^{12}	K1	Cu1^{12}	38.69(4)
Cu1	Se2	K1^{5}	82.30(7)	Se2^{4}	K1	Cu1^{8}	146.58(9)
K1^{5}	Se2	K1^{4}	105.60(6)	Se2^{4}	K1	K1^{3}	90.0
K1^{6}	Se2	K1^{5}	70.81(6)	Se2^{11}	K1	K1^{1}	125.41(3)
K1^{6}	Se2	K1^{4}	105.60(6)	Se2^{4}	K1	K1^{1}	90.0
Hf1	Se3	K1^{7}	141.54(4)	Se2^{12}	K1	K1^{1}	54.59(3)
Hf1	Se3	K1^{8}	141.54(4)	Se2^{12}	K1	K1^{3}	125.41(3)
Cu1	Se3	Hf1	75.12(5)	Se2^{11}	K1	K1^{3}	54.59(3)
Cu1^{1}	Se3	Hf1	75.12(5)	Se3^{7}	K1	Se2^{12}	72.91(5)
Cu1^{9}	Se3	Hf1	114.02(7)	Se3^{8}	K1	Se2^{4}	141.27(5)
Cu1^{1}	Se3	Cu1^{9}	65.71(6)	Se3^{8}	K1	Se2^{11}	72.91(5)
Cu1	Se3	Cu1^{1}	103.29(8)	Se3^{8}	K1	Se2^{12}	112.87(8)
Cu1	Se3	Cu1^{9}	65.71(6)	Se3^{7}	K1	Se2^{4}	141.27(5)
Cu1	Se3	K1^{7}	140.89(8)	Se3^{7}	K1	Se2^{11}	112.87(8)
Cu1	Se3	K1^{8}	81.49(5)	Se3^{8}	K1	Se3^{7}	72.18(6)
Cu1^{1}	Se3	K1^{8}	140.89(8)	Se3^{7}	K1	Cu1^{12}	40.38(4)
Cu1^{9}	Se3	K1^{7}	82.17(7)	Se3^{8}	K1	Cu1^{8}	39.89(3)
Cu1^{9}	Se3	K1^{8}	82.17(7)	Se3^{7}	K1	Cu1^{8}	39.89(3)
Cu1^{1}	Se3	K1^{7}	81.49(5)	Se3^{7}	K1	Cu1^{11}	80.13(6)
K1^{8}	Se3	K1^{7}	72.18(6)	Se3^{8}	K1	Cu1^{12}	80.13(6)
Hf1^{3}	Se1	Hf1^{2}	97.17(4)	Se3^{8}	K1	Cu1^{11}	40.38(4)
Hf1^{3}	Se1	Hf1	91.29(4)	Se3^{7}	K1	K1^{3}	126.09(3)
Hf1	Se1	Hf1^{2}	97.17(4)	Se3^{7}	K1	K1^{1}	53.91(3)
Hf1	Se1	K1^{1}	94.04(4)	Se3^{8}	K1	K1^{1}	126.09(3)
Hf1^{2}	Se1	K1	103.95(6)	Se3^{8}	K1	K1^{3}	53.91(3)
Hf1	Se1	K1	157.36(7)	Se1^{3}	K1	Se2^{12}	141.93(10)
Hf1^{3}	Se1	K1	94.04(4)	Se1^{3}	K1	Se2^{4}	67.65(5)
Hf1^{3}	Se1	K1^{1}	157.36(7)	Se1^{3}	K1	Se2^{11}	95.54(4)
Hf1^{2}	Se1	K1^{1}	103.95(6)	Se1	K1	Se2^{11}	141.93(10)
K1	Se1	K1^{1}	73.18(6)	Se1	K1	Se2^{12}	95.54(4)
Hf1	Cu1	Hf1^{3}	77.84(5)	Se1	K1	Se2^{4}	67.65(5)
Hf1^{3}	Cu1	K1^{6}	77.25(5)	Se1^{3}	K1	Se3^{8}	95.44(4)
Hf1	Cu1	K1^{6}	114.14(6)	Se1	K1	Se3^{7}	95.44(4)
Hf1^{3}	Cu1	K1^{5}	114.14(6)	Se1	K1	Se3^{8}	142.84(11)
Hf1^{3}	Cu1	K1^{8}	106.81(6)	Se1^{3}	K1	Se3^{7}	142.84(11)
Hf1	Cu1	K1^{5}	77.25(5)	Se1^{3}	K1	Se1	73.18(6)

Table S21. Bond Angles for $KCuHfSe_3$.

Atom	Atom	Atom	Angle/°	Atom	Atom	Atom	Angle/°
Hf1	Cu1	K1^{8}	106.81(6)	Se1	K1	Cu1^{11}	173.36(5)
Se2	Cu1	Hf1^{3}	56.33(4)	Se1	K1	Cu1^{12}	113.32(2)
Se2	Cu1	Hf1	56.33(4)	Se1^{3}	K1	Cu1^{11}	113.32(2)
Se2	Cu1	Se3^{9}	105.04(8)	Se1	K1	Cu1^{8}	134.98(6)
Se2	Cu1	Se3^{3}	109.99(6)	Se1^{3}	K1	Cu1^{8}	134.98(6)
Se2	Cu1	Se3	109.99(6)	Se1^{3}	K1	Cu1^{12}	173.36(5)
Se2	Cu1	Cu1^{10}	123.59(8)	Se1	K1	K1^{1}	53.41(3)
Se2	Cu1	Cu1^{9}	123.59(8)	Se1^{3}	K1	K1^{3}	53.41(3)
Se2	Cu1	K1^{6}	59.01(6)	Se1^{3}	K1	K1^{1}	126.59(3)
Se2	Cu1	K1^{8}	156.38(9)	Se1	K1	K1^{3}	126.59(3)
Se2	Cu1	K1^{5}	59.01(6)	Cu1^{8}	K1	Cu1^{11}	41.37(4)
Se3^{3}	Cu1	Hf1^{3}	54.14(4)	Cu1^{12}	K1	Cu1^{11}	60.16(5)
Se3	Cu1	Hf1^{3}	113.55(7)	Cu1^{8}	K1	Cu1^{12}	41.37(4)
Se3^{3}	Cu1	Hf1	113.55(7)	Cu1^{11}	K1	K1^{3}	59.92(2)
Se3^{9}	Cu1	Hf1^{3}	132.14(5)	Cu1^{12}	K1	K1^{1}	59.92(2)
Se3^{9}	Cu1	Hf1	132.14(5)	Cu1^{12}	K1	K1^{3}	120.08(2)
Se3	Cu1	Hf1	54.14(4)	Cu1^{8}	K1	K1^{1}	90.0
Se3	Cu1	Se3^{9}	114.30(6)	Cu1^{8}	K1	K1^{3}	90.0
Se3^{3}	Cu1	Se3^{9}	114.29(6)	Cu1^{11}	K1	K1^{1}	120.08(2)
Se3^{3}	Cu1	Se3	103.29(8)	K1^{1}	K1	K1^{3}	180.0
Se3	Cu1	Cu1^{9}	58.16(5)				

^{1}+X,1+Y,+Z; ^{2}1-X,2-Y,2-Z; ^{3}+X,-1+Y,+Z; ^{4}1-X,1-Y,2-Z; ^{5}-1+X,1+Y,+Z; ^{6}-1+X,+Y,+Z; ^{7}1-X,2-Y,1-Z; ^{8}1-X,1-Y,1-Z; ^{9}-X,2-Y,1-Z; ^{10}-X,1-Y,1-Z; ^{11}1+X,-1+Y,+Z; ^{12}1+X,+Y,+Z

$RbCuHfSe_3$ (S22-S26)

Table S22. Crystal data and structure refinement for $RbCuHfSe_3$.

Identification code	$RbCuHfSe_3$
Empirical formula	$RbCuHfSe_3$
Formula weight	564.38
Temperature/K	173.00(10)
Crystal system	orthorhombic
Space group	Cmcm
a/Å	3.8665(3)
b/Å	15.0787(11)
c/Å	10.1499(7)
α/°	90

β/°	90
γ/°	90
Volume/Å^3	591.76(8)
Z	4
ρ_{calc}g/cm^3	6.335
μ/mm^{-1}	66.088
F(000)	960.0
Crystal size/mm^3	0.234 × 0.033 × 0.018
Radiation	Cu Kα (λ = 1.54184)
2Θ range for data collection/°	14.636 to 147.252
Index ranges	-3 ≤ h ≤ 4, -18 ≤ k ≤ 14, -11 ≤ l ≤ 12
Reflections collected	789
Independent reflections	341 [R_{int} = 0.0252, R_{sigma} = 0.0231]
Data/restraints/parameters	341/0/24
Goodness-of-fit on F^2	1.096
Final R indexes [I>=2σ (I)]	R_1 = 0.0379, wR_2 = 0.1062
Final R indexes [all data]	R_1 = 0.0381, wR_2 = 0.1068
Largest diff. peak/hole / e Å^{-3}	1.91/-1.68

Table S23. Fractional Atomic Coordinates (×10^4) and Equivalent Isotropic Displacement Parameters (Å^2×10^3) for $RbCuHfSe_3$. U_{eq} is defined as 1/3 of the trace of the orthogonalised U_{IJ} tensor.

Atom	x	y	z	U(eq)	Wyckoff site	Symmetry
Hf1	10000	5000	5000	12.6(5)	4b	2/m..
Rb1	-5000	7463.2(9)	7500	17.3(5)	4c	m2m
Se2	5000	3800.7(6)	5473.6(9)	12.7(5)	8f	m..
Se1	0	5620.4(9)	7500	11.7(5)	4c	m2m
Cu1	5000	4634.1(14)	7500	15.7(6)	4c	m2m

Table S24. Anisotropic Displacement Parameters (Å^2×10^3) for $RbCuHfSe_3$. The Anisotropic displacement factor exponent takes the form: $-2\pi^2[h^2a^{*2}U_{11}+2hka^*b^*U_{12}+\ldots]$.

Atom	U_{11}	U_{22}	U_{33}	U_{23}	U_{13}	U_{12}
Hf1	5.2(6)	19.0(6)	13.7(6)	0.1(2)	0	0
Rb1	8.2(8)	22.5(8)	21.2(8)	0	0	0
Se2	6.0(7)	18.0(6)	14.2(8)	0.2(4)	0	0
Se1	4.9(8)	20.3(8)	9.9(7)	0	0	0
Cu1	10.6(12)	21.6(11)	14.9(11)	0	0	0

Table S25. Bond Lengths for $RbCuHfSe_3$.

Atom	Atom	Length/Å	Atom	Atom	Length/Å
Hf1	$Se2^{1}$	2.6904(7)	Rb1	$Se2^{6}$	3.4692(11)
Hf1	$Se2^{2}$	2.6904(7)	Rb1	$Se2^{7}$	3.4692(11)
Hf1	Se2	2.6905(7)	Rb1	$Se2^{8}$	3.4692(11)
Hf1	$Se2^{3}$	2.6904(7)	Rb1	$Se2^{9}$	3.5696(12)
Hf1	$Se1^{2}$	2.7044(5)	Rb1	$Se2^{10}$	3.4692(11)
Hf1	$Se1^{3}$	2.7044(5)	Rb1	Se1	3.3850(16)
Hf1	$Cu1^{3}$	3.2374(4)	Rb1	$Se1^{4}$	3.3850(16)
Hf1	Cu1	3.2374(4)	Rb1	$Cu1^{6}$	3.802(2)
Hf1	$Cu1^{1}$	3.2374(4)	Rb1	$Cu1^{8}$	3.802(2)
Hf1	$Cu1^{2}$	3.2374(4)	Se2	Cu1	2.4103(14)
Rb1	$Rb1^{2}$	3.8665(3)	Se1	Cu1	2.4392(15)
Rb1	$Rb1^{4}$	3.8665(3)	Se1	$Cu1^{4}$	2.4392(15)
Rb1	$Se2^{5}$	3.5696(12)			

[1]2-X,1-Y,1-Z; [2]1+X,+Y,+Z; [3]1-X,1-Y,1-Z; [4]-1+X,+Y,+Z; [5]-X,1-Y,1/2+Z; [6]-1/2+X,1/2+Y,+Z; [7]-3/2+X,1/2+Y,3/2-Z; [8]-3/2+X,1/2+Y,+Z; [9]-X,1-Y,1-Z; [10]-1/2+X,1/2+Y,3/2-Z

Table S26. Bond Angles for $RbCuHfSe_3$.

Atom	Atom	Atom	Angle/°	Atom	Atom	Atom	Angle/°
$Se2^{1}$	Hf1	$Se2^{2}$	91.87(3)	$Se1^{4}$	Rb1	$Se2^{9}$	64.01(2)
$Se2^{2}$	Hf1	Se2	88.13(3)	$Se1^{4}$	Rb1	$Se2^{6}$	99.147(18)
$Se2^{1}$	Hf1	Se2	180.0	Se1	Rb1	$Se2^{8}$	99.147(18)
$Se2^{1}$	Hf1	$Se2^{3}$	88.13(3)	$Se1^{4}$	Rb1	$Se2^{5}$	99.147(18)
$Se2^{2}$	Hf1	$Se2^{3}$	180.0	$Se1^{4}$	Rb1	$Se2^{10}$	64.01(2)
$Se2^{3}$	Hf1	Se2	91.87(3)	Se1	Rb1	$Se2^{5}$	142.70(2)
$Se2^{3}$	Hf1	$Se1^{3}$	93.72(3)	Se1	Rb1	$Se2^{6}$	142.70(2)
$Se2^{2}$	Hf1	$Se1^{2}$	93.72(3)	Se1	Rb1	$Se2^{9}$	64.01(2)
$Se2^{3}$	Hf1	$Se1^{2}$	86.28(3)	$Se1^{4}$	Rb1	Se1	69.66(4)
Se2	Hf1	$Se1^{2}$	86.28(3)	Se1	Rb1	$Cu1^{8}$	114.61(2)
$Se2^{1}$	Hf1	$Se1^{3}$	86.28(3)	$Se1^{4}$	Rb1	$Cu1^{5}$	114.61(2)
$Se2^{2}$	Hf1	$Se1^{3}$	86.28(3)	$Se1^{4}$	Rb1	$Cu1^{8}$	175.74(3)
$Se2^{1}$	Hf1	$Se1^{2}$	93.72(3)	Se1	Rb1	$Cu1^{5}$	175.74(3)
Se2	Hf1	$Se1^{3}$	93.72(3)	$Cu1^{8}$	Rb1	$Rb1^{3}$	59.43(2)
Se2	Hf1	$Cu1^{2}$	79.95(3)	$Cu1^{5}$	Rb1	$Rb1^{3}$	120.57(2)
$Se2^{1}$	Hf1	$Cu1^{1}$	46.87(3)	$Cu1^{8}$	Rb1	$Rb1^{4}$	120.57(2)
$Se2^{1}$	Hf1	$Cu1^{3}$	79.95(3)	$Cu1^{5}$	Rb1	$Rb1^{4}$	59.43(2)
Se2	Hf1	Cu1	46.87(3)	$Cu1^{8}$	Rb1	$Cu1^{5}$	61.13(4)
$Se2^{2}$	Hf1	$Cu1^{1}$	100.05(3)	Hf1	Se2	$Hf1^{4}$	91.87(3)
$Se2^{1}$	Hf1	Cu1	133.13(3)	$Hf1^{4}$	Se2	$Rb1^{11}$	95.522(17)

Table S26. Bond Angles for $RbCuHfSe_3$.

Atom	Atom	Atom	Angle/°	Atom	Atom	Atom	Angle/°
$Se2^{3}$	Hf1	Cu1	100.05(3)	$Hf1^{4}$	Se2	$Rb1^{10}$	101.99(3)
$Se2^{2}$	Hf1	$Cu1^{3}$	133.13(3)	Hf1	Se2	$Rb1^{10}$	101.99(3)
$Se2^{3}$	Hf1	$Cu1^{3}$	46.87(3)	$Hf1^{4}$	Se2	$Rb1^{12}$	153.78(3)
$Se2^{3}$	Hf1	$Cu1^{2}$	133.13(3)	Hf1	Se2	$Rb1^{11}$	153.78(3)
$Se2^{3}$	Hf1	$Cu1^{1}$	79.95(3)	Hf1	Se2	$Rb1^{12}$	95.522(17)
Se2	Hf1	$Cu1^{3}$	100.05(3)	$Rb1^{12}$	Se2	$Rb1^{11}$	67.73(3)
$Se2^{2}$	Hf1	$Cu1^{2}$	46.87(3)	$Rb1^{12}$	Se2	$Rb1^{10}$	101.00(2)
$Se2^{2}$	Hf1	Cu1	79.95(3)	$Rb1^{11}$	Se2	$Rb1^{10}$	101.00(2)
Se2	Hf1	$Cu1^{1}$	133.13(3)	Cu1	Se2	Hf1	78.58(3)
$Se2^{1}$	Hf1	$Cu1^{2}$	100.05(3)	Cu1	Se2	$Hf1^{4}$	78.58(3)
$Se1^{2}$	Hf1	$Se1^{3}$	180.0	Cu1	Se2	$Rb1^{12}$	78.30(5)
$Se1^{3}$	Hf1	$Cu1^{3}$	47.43(3)	Cu1	Se2	$Rb1^{10}$	179.15(6)
$Se1^{3}$	Hf1	$Cu1^{1}$	132.57(3)	Cu1	Se2	$Rb1^{11}$	78.30(5)
$Se1^{2}$	Hf1	$Cu1^{1}$	47.43(3)	$Hf1^{4}$	Se1	$Hf1^{13}$	139.52(6)
$Se1^{3}$	Hf1	Cu1	47.43(3)	$Hf1^{13}$	Se1	$Rb1^{3}$	106.50(2)
$Se1^{3}$	Hf1	$Cu1^{2}$	132.57(3)	$Hf1^{13}$	Se1	Rb1	106.50(2)
$Se1^{2}$	Hf1	$Cu1^{2}$	47.43(3)	$Hf1^{4}$	Se1	Rb1	106.50(2)
$Se1^{2}$	Hf1	$Cu1^{3}$	132.57(3)	$Hf1^{4}$	Se1	$Rb1^{3}$	106.50(2)
$Se1^{2}$	Hf1	Cu1	132.57(3)	Rb1	Se1	$Rb1^{3}$	69.66(4)
$Cu1^{3}$	Hf1	Cu1	73.334(11)	Cu1	Se1	$Hf1^{4}$	77.82(3)
$Cu1^{2}$	Hf1	$Cu1^{3}$	180.0	$Cu1^{4}$	Se1	$Hf1^{4}$	77.82(3)
$Cu1^{2}$	Hf1	Cu1	106.666(11)	Cu1	Se1	$Hf1^{13}$	77.82(3)
$Cu1^{3}$	Hf1	$Cu1^{1}$	106.666(11)	$Cu1^{4}$	Se1	$Hf1^{13}$	77.82(3)
$Cu1^{2}$	Hf1	$Cu1^{1}$	73.334(11)	Cu1	Se1	$Rb1^{3}$	92.74(4)
Cu1	Hf1	$Cu1^{1}$	180.0	$Cu1^{4}$	Se1	$Rb1^{3}$	162.40(5)
$Rb1^{4}$	Rb1	$Rb1^{3}$	180.0	Cu1	Se1	Rb1	162.40(5)
$Se2^{5}$	Rb1	$Rb1^{3}$	123.866(13)	$Cu1^{4}$	Se1	Rb1	92.74(4)
$Se2^{6}$	Rb1	$Rb1^{4}$	56.133(13)	Cu1	Se1	$Cu1^{4}$	104.86(9)
$Se2^{7}$	Rb1	$Rb1^{4}$	123.866(13)	$Hf1^{13}$	Cu1	Hf1	160.37(7)
$Se2^{7}$	Rb1	$Rb1^{3}$	56.133(13)	$Hf1^{4}$	Cu1	Hf1	73.334(11)
$Se2^{8}$	Rb1	$Rb1^{4}$	123.866(13)	$Hf1^{4}$	Cu1	$Hf1^{13}$	103.220(17)
$Se2^{9}$	Rb1	$Rb1^{3}$	90.0	$Hf1^{14}$	Cu1	$Hf1^{4}$	160.37(7)
$Se2^{6}$	Rb1	$Rb1^{3}$	123.866(13)	$Hf1^{14}$	Cu1	$Hf1^{13}$	73.334(11)
$Se2^{5}$	Rb1	$Rb1^{4}$	56.133(13)	$Hf1^{14}$	Cu1	Hf1	103.220(17)
$Se2^{8}$	Rb1	$Rb1^{3}$	56.133(13)	$Hf1^{13}$	Cu1	$Rb1^{12}$	116.77(4)
$Se2^{10}$	Rb1	$Rb1^{4}$	90.0	$Hf1^{13}$	Cu1	$Rb1^{11}$	80.97(2)
$Se2^{10}$	Rb1	$Rb1^{3}$	90.0	$Hf1^{14}$	Cu1	$Rb1^{12}$	80.97(2)

Table S26. Bond Angles for $RbCuHfSe_3$.

Atom	Atom	Atom	Angle/°	Atom	Atom	Atom	Angle/°
$Se2^{9}$	Rb1	$Rb1^{4}$	90.0	$Hf1^{4}$	Cu1	$Rb1^{12}$	116.77(4)
$Se2^{6}$	Rb1	$Se2^{5}$	72.72(4)	$Hf1^{14}$	Cu1	$Rb1^{11}$	116.77(4)
$Se2^{6}$	Rb1	$Se2^{8}$	108.91(5)	Hf1	Cu1	$Rb1^{11}$	116.77(4)
$Se2^{8}$	Rb1	$Se2^{5}$	67.73(3)	$Hf1^{4}$	Cu1	$Rb1^{11}$	80.97(2)
$Se2^{10}$	Rb1	$Se2^{9}$	115.46(4)	Hf1	Cu1	$Rb1^{12}$	80.97(2)
$Se2^{7}$	Rb1	$Se2^{6}$	67.73(3)	$Rb1^{11}$	Cu1	$Rb1^{12}$	61.13(4)
$Se2^{6}$	Rb1	$Se2^{10}$	144.261(14)	Se2	Cu1	$Hf1^{4}$	54.549(16)
$Se2^{5}$	Rb1	$Se2^{9}$	144.261(14)	$Se2^{15}$	Cu1	$Hf1^{13}$	54.549(16)
$Se2^{8}$	Rb1	$Se2^{10}$	79.00(2)	$Se2^{15}$	Cu1	$Hf1^{14}$	54.549(16)
$Se2^{5}$	Rb1	$Se2^{10}$	79.00(2)	$Se2^{15}$	Cu1	$Hf1^{4}$	139.26(4)
$Se2^{7}$	Rb1	$Se2^{10}$	144.261(14)	Se2	Cu1	Hf1	54.549(16)
$Se2^{7}$	Rb1	$Se2^{9}$	79.00(2)	Se2	Cu1	$Hf1^{14}$	139.26(4)
$Se2^{6}$	Rb1	$Se2^{9}$	79.00(2)	$Se2^{15}$	Cu1	Hf1	139.26(4)
$Se2^{7}$	Rb1	$Se2^{8}$	72.72(4)	Se2	Cu1	$Hf1^{13}$	139.26(4)
$Se2^{8}$	Rb1	$Se2^{9}$	144.261(14)	$Se2^{15}$	Cu1	$Rb1^{11}$	63.33(4)
$Se2^{7}$	Rb1	$Se2^{5}$	108.91(5)	$Se2^{15}$	Cu1	$Rb1^{12}$	63.33(4)
$Se2^{10}$	Rb1	$Cu1^{8}$	117.370(17)	Se2	Cu1	$Rb1^{12}$	63.33(4)
$Se2^{7}$	Rb1	$Cu1^{5}$	77.46(3)	Se2	Cu1	$Rb1^{11}$	63.33(4)
$Se2^{8}$	Rb1	$Cu1^{5}$	77.46(3)	$Se2^{15}$	Cu1	Se2	117.16(9)
$Se2^{6}$	Rb1	$Cu1^{8}$	77.46(3)	Se2	Cu1	$Se1^{3}$	108.535(18)
$Se2^{6}$	Rb1	$Cu1^{5}$	38.38(2)	Se2	Cu1	Se1	108.535(18)
$Se2^{7}$	Rb1	$Cu1^{8}$	38.38(2)	$Se2^{15}$	Cu1	Se1	108.535(18)
$Se2^{8}$	Rb1	$Cu1^{8}$	38.38(2)	$Se2^{15}$	Cu1	$Se1^{3}$	108.535(18)
$Se2^{5}$	Rb1	$Cu1^{8}$	77.46(3)	$Se1^{3}$	Cu1	Hf1	54.744(15)
$Se2^{5}$	Rb1	$Cu1^{5}$	38.38(2)	Se1	Cu1	Hf1	111.68(5)
$Se2^{10}$	Rb1	$Cu1^{5}$	117.370(17)	$Se1^{3}$	Cu1	$Hf1^{4}$	111.68(5)
$Se2^{9}$	Rb1	$Cu1^{5}$	117.370(17)	Se1	Cu1	$Hf1^{4}$	54.744(15)
$Se2^{9}$	Rb1	$Cu1^{8}$	117.370(17)	$Se1^{3}$	Cu1	$Hf1^{13}$	111.68(5)
$Se1^{4}$	Rb1	$Rb1^{4}$	55.172(19)	$Se1^{3}$	Cu1	$Hf1^{14}$	54.744(15)
$Se1^{4}$	Rb1	$Rb1^{3}$	124.829(19)	Se1	Cu1	$Hf1^{14}$	111.68(5)
Se1	Rb1	$Rb1^{4}$	124.829(19)	Se1	Cu1	$Hf1^{13}$	54.744(15)
Se1	Rb1	$Rb1^{3}$	55.173(19)	Se1	Cu1	$Rb1^{11}$	97.01(4)
$Se1^{4}$	Rb1	$Se2^{7}$	142.70(2)	$Se1^{3}$	Cu1	$Rb1^{11}$	158.14(6)
Se1	Rb1	$Se2^{7}$	99.147(18)	Se1	Cu1	$Rb1^{12}$	158.14(6)
Se1	Rb1	$Se2^{10}$	64.01(2)	$Se1^{3}$	Cu1	$Rb1^{12}$	97.01(4)
$Se1^{4}$	Rb1	$Se2^{8}$	142.70(2)	Se1	Cu1	$Se1^{3}$	104.86(9)

[1]2-X,1-Y,1-Z; [2]1-X,1-Y,1-Z; [3]1+X,+Y,+Z; [4]-1+X,+Y,+Z; [5]-3/2+X,1/2+Y,+Z; [6]-3/2+X,1/2+Y,3/2-Z; [7]-1/2+X,1/2+Y,3/2-Z; [8]-1/2+X,1/2+Y,+Z; [9]-X,1-Y,1/2+Z; [10]-X,1-Y,1-Z; [11]1/2+X,-1/2+Y,+Z; [12]3/2+X,-1/2+Y,+Z; [13]1-X,1-Y,1/2+Z; [14]2-X,1-Y,1/2+Z; [15]+X,+Y,3/2-Z

$CsCuHfSe_3$ tables (S27-S31)

Table S27. Crystal data and structure refinement for $CsCuHfSe_3$.

Identification code	$CsCuHfSe_3$
Empirical formula	$CsCuHfSe_3$
Formula weight	611.82
Temperature/K	173.02(10)
Crystal system	orthorhombic
Space group	Cmcm
a/Å	3.88180(10)
b/Å	15.8371(4)
c/Å	10.1703(3)
α/°	90
β/°	90
γ/°	90
Volume/Å^3	625.23(3)
Z	4
ρ_{calc}g/cm^3	6.500
μ/mm^{-1}	97.864
F(000)	1032.0
Crystal size/mm^3	0.15 × 0.02 × 0.02
Radiation	Cu Kα (λ = 1.54184)
2Θ range for data collection/°	14.176 to 151.238
Index ranges	-4 ≤ h ≤ 3, -16 ≤ k ≤ 19, -12 ≤ l ≤ 9
Reflections collected	836
Independent reflections	364 [R_{int} = 0.0263, R_{sigma} = 0.0280]
Data/restraints/parameters	364/0/24
Goodness-of-fit on F^2	1.141
Final R indexes [I>=2σ (I)]	R_1 = 0.0404, wR_2 = 0.1082
Final R indexes [all data]	R_1 = 0.0410, wR_2 = 0.1089
Largest diff. peak/hole / e Å^{-3}	2.39/-1.89

Table S28. Fractional Atomic Coordinates (×10^4) and Equivalent Isotropic Displacement Parameters (Å^2×10^3) for $CsCuHfSe_3$. U_{eq} is defined as 1/3 of the trace of the orthogonalised U_{IJ} tensor.

Atom	x	y	z	U(eq)	Wyckoff site	Symmetry
Hf1	10000	5000	5000	15.1(4)	4b	2/m..

Table S28. Fractional Atomic Coordinates (×10^4) and Equivalent Isotropic Displacement Parameters (Å^2×10^3) for $CsCuHfSe_3$. U_{eq} is defined as 1/3 of the trace of the orthogonalised U_{IJ} tensor.

Atom	x	y	z	U(eq)	Wyckoff site	Symmetry
Cs1	-5000	7450.0(6)	2500	19.6(5)	4c	m2m
Se2	5000	3859.8(7)	4529.7(11)	14.8(5)	8f	m..
Se1	0	5578.3(10)	2500	15.0(5)	4c	m2m
Cu1	5000	4641.4(16)	2500	19.1(7)	4c	m2m

Table S29. Anisotropic Displacement Parameters (Å^2×10^3) for $CsCuHfSe_3$. The Anisotropic displacement factor exponent takes the form: $-2\pi^2[h^2a^{*2}U_{11}+2hka^*b^*U_{12}+\ldots]$.

Atom	U_{11}	U_{22}	U_{33}	U_{23}	U_{13}	U_{12}
Hf1	11.5(7)	16.5(6)	17.3(6)	-0.2(3)	0	0
Cs1	16.3(8)	18.5(7)	24.0(7)	0	0	0
Se2	11.4(8)	16.1(7)	17.0(8)	-0.5(4)	0	0
Se1	12.7(11)	18.5(9)	13.8(8)	0	0	0
Cu1	18.2(16)	20.1(12)	19.1(12)	0	0	0

Table S30. Bond Lengths for $CsCuHfSe_3$.

Atom	Atom	Length/Å	Atom	Atom	Length/Å
Hf1	Se2	2.6938(8)	Cs1	Se2^6	3.6645(12)
Hf1	Se2^1	2.6938(8)	Cs1	Se2^7	3.6074(11)
Hf1	Se2^2	2.6938(8)	Cs1	Se2^8	3.6645(12)
Hf1	Se2^3	2.6938(8)	Cs1	Se2^9	3.6074(11)
Hf1	Se1^3	2.7025(6)	Cs1	Se2^{10}	3.6074(11)
Hf1	Se1^2	2.7025(6)	Cs1	Se1	3.5432(16)
Hf1	Cu1^2	3.2487(5)	Cs1	Se1^4	3.5431(16)
Hf1	Cu1^3	3.2487(5)	Cs1	Cu1^7	3.977(2)
Hf1	Cu1^1	3.2487(5)	Cs1	Cu1^5	3.977(2)
Hf1	Cu1	3.2487(5)	Se2	Cu1	2.4070(16)
Cs1	Cs1^3	3.88180(10)	Se1	Cu1	2.4430(18)
Cs1	Cs1^4	3.88180(10)	Se1	Cu1^4	2.4430(18)
Cs1	Se2^5	3.6074(11)			

^{1}2-X,1-Y,1-Z; ^{2}1-X,1-Y,1-Z; ^{3}1+X,+Y,+Z; ^{4}-1+X,+Y,+Z; ^{5}-3/2+X,1/2+Y,+Z; ^{6}-X,1-Y,-1/2+Z; ^{7}-1/2+X,1/2+Y,+Z; ^{8}-X,1-Y,1-Z; ^{9}-3/2+X,1/2+Y,1/2-Z; ^{10}-1/2+X,1/2+Y,1/2-Z

Table S31. Bond Angles for $CsCuHfSe_3$.

Atom	Atom	Atom	Angle/°	Atom	Atom	Atom	Angle/°
Se2	Hf1	Se2^{1}	180.0	Se1^{4}	Cs1	Se2^{6}	102.890(17)
Se2	Hf1	Se2^{2}	92.19(3)	Se1^{4}	Cs1	Se2^{5}	144.34(2)
Se2^{1}	Hf1	Se2^{2}	87.81(3)	Se1	Cs1	Se2^{7}	144.34(2)
Se2^{1}	Hf1	Se2^{3}	92.19(3)	Se1^{4}	Cs1	Se2^{7}	102.890(18)
Se2	Hf1	Se2^{3}	87.81(3)	Se1	Cs1	Se2^{6}	144.34(2)
Se2^{3}	Hf1	Se2^{2}	180.0	Se1	Cs1	Se2^{10}	61.733(17)
Se2^{3}	Hf1	Se1^{2}	86.55(3)	Se1	Cs1	Se2^{8}	61.733(17)
Se2^{3}	Hf1	Se1^{3}	93.45(3)	Se1	Cs1	Se2^{5}	102.890(18)
Se2	Hf1	Se1^{2}	93.45(3)	Se1^{4}	Cs1	Se2^{9}	144.34(2)
Se2^{1}	Hf1	Se1^{2}	86.55(3)	Se1	Cs1	Se2^{9}	102.890(18)
Se2^{2}	Hf1	Se1^{3}	86.55(3)	Se1^{4}	Cs1	Se2^{10}	61.733(17)
Se2^{1}	Hf1	Se1^{3}	93.45(3)	Se1^{4}	Cs1	Se2^{8}	61.733(17)
Se2	Hf1	Se1^{3}	86.55(3)	Se1^{4}	Cs1	Se1	66.43(3)
Se2^{2}	Hf1	Se1^{2}	93.45(3)	Se1	Cs1	Cu1^{5}	117.57(2)
Se2^{1}	Hf1	Cu1^{2}	79.96(4)	Se1	Cs1	Cu1^{6}	176.00(3)
Se2^{3}	Hf1	Cu1^{2}	133.36(4)	Se1^{4}	Cs1	Cu1^{6}	117.57(2)
Se2^{2}	Hf1	Cu1^{3}	133.36(4)	Se1^{4}	Cs1	Cu1^{5}	176.00(3)
Se2	Hf1	Cu1^{3}	79.96(4)	Cu1^{5}	Cs1	Cu1^{6}	58.43(4)
Se2^{3}	Hf1	Cu1^{1}	100.04(4)	Hf1	Se2	Hf1^{4}	92.19(3)
Se2^{3}	Hf1	Cu1^{3}	46.64(4)	Hf1^{4}	Se2	Cs1^{11}	154.71(4)
Se2^{1}	Hf1	Cu1^{1}	46.64(4)	Hf1	Se2	Cs1^{11}	97.402(14)
Se2	Hf1	Cu1^{2}	100.04(4)	Hf1	Se2	Cs1^{12}	154.71(4)
Se2^{3}	Hf1	Cu1	79.96(4)	Hf1	Se2	Cs1^{10}	103.48(3)
Se2^{1}	Hf1	Cu1^{3}	100.04(4)	Hf1^{4}	Se2	Cs1^{10}	103.48(3)
Se2	Hf1	Cu1^{1}	133.36(4)	Hf1^{4}	Se2	Cs1^{12}	97.402(14)
Se2	Hf1	Cu1	46.64(4)	Cs1^{12}	Se2	Cs1^{11}	65.10(2)
Se2^{2}	Hf1	Cu1	100.04(4)	Cs1^{12}	Se2	Cs1^{10}	96.97(2)
Se2^{2}	Hf1	Cu1^{1}	79.96(4)	Cs1^{11}	Se2	Cs1^{10}	96.97(2)
Se2^{1}	Hf1	Cu1	133.36(4)	Cu1	Se2	Hf1^{4}	78.90(4)
Se2^{2}	Hf1	Cu1^{2}	46.64(4)	Cu1	Se2	Hf1	78.90(4)
Se1^{3}	Hf1	Se1^{2}	180.0	Cu1	Se2	Cs1^{11}	80.07(5)
Se1^{3}	Hf1	Cu1^{2}	132.62(3)	Cu1	Se2	Cs1^{12}	80.07(5)
Se1^{2}	Hf1	Cu1^{1}	132.62(3)	Cu1	Se2	Cs1^{10}	176.47(7)
Se1^{3}	Hf1	Cu1^{1}	47.38(3)	Hf1^{13}	Se1	Hf1^{4}	140.38(7)
Se1^{3}	Hf1	Cu1	132.62(3)	Hf1^{4}	Se1	Cs1	106.47(2)
Se1^{2}	Hf1	Cu1	47.38(3)	Hf1^{4}	Se1	Cs1^{2}	106.47(2)
Se1^{2}	Hf1	Cu1^{2}	47.38(3)	Hf1^{13}	Se1	Cs1	106.47(2)

Table S31. Bond Angles for $CsCuHfSe_3$.

Atom	Atom	Atom	Angle/°	Atom	Atom	Atom	Angle/°
Se1^{2}	Hf1	Cu1^{3}	132.62(3)	Hf1^{13}	Se1	Cs1^{2}	106.47(2)
Se1^{3}	Hf1	Cu1^{3}	47.38(3)	Cs1	Se1	Cs1^{2}	66.43(3)
Cu1^{3}	Hf1	Cu1^{2}	180.0	Cu1	Se1	Hf1^{4}	78.12(3)
Cu1^{1}	Hf1	Cu1	180.0	Cu1	Se1	Hf1^{13}	78.12(3)
Cu1^{2}	Hf1	Cu1	73.373(12)	Cu1^{4}	Se1	Hf1^{13}	78.12(3)
Cu1^{1}	Hf1	Cu1^{3}	73.373(12)	Cu1^{4}	Se1	Hf1^{4}	78.12(3)
Cu1^{3}	Hf1	Cu1	106.627(12)	Cu1^{4}	Se1	Cs1^{2}	160.61(6)
Cu1^{1}	Hf1	Cu1^{2}	106.627(12)	Cu1	Se1	Cs1	160.61(6)
Cs1^{4}	Cs1	Cs1^{2}	180.0	Cu1	Se1	Cs1^{2}	94.18(5)
Cs1^{2}	Cs1	Cu1^{5}	60.784(19)	Cu1^{4}	Se1	Cs1	94.18(5)
Cs1^{2}	Cs1	Cu1^{6}	119.215(19)	Cu1^{4}	Se1	Cu1	105.21(11)
Cs1^{4}	Cs1	Cu1^{5}	119.215(19)	Hf1	Cu1	Hf1^{13}	159.87(9)
Cs1^{4}	Cs1	Cu1^{6}	60.784(19)	Hf1^{4}	Cu1	Hf1^{14}	159.87(9)
Se2^{6}	Cs1	Cs1^{4}	57.450(12)	Hf1^{13}	Cu1	Hf1^{14}	73.373(12)
Se2^{7}	Cs1	Cs1^{4}	57.450(12)	Hf1	Cu1	Hf1^{14}	103.01(2)
Se2^{8}	Cs1	Cs1^{4}	90.0	Hf1	Cu1	Hf1^{4}	73.373(12)
Se2^{9}	Cs1	Cs1^{4}	122.549(12)	Hf1^{13}	Cu1	Hf1^{4}	103.01(2)
Se2^{8}	Cs1	Cs1^{2}	90.0	Hf1^{4}	Cu1	Cs1^{11}	116.37(5)
Se2^{7}	Cs1	Cs1^{2}	122.549(12)	Hf1^{13}	Cu1	Cs1^{11}	116.37(5)
Se2^{6}	Cs1	Cs1^{2}	122.549(12)	Hf1	Cu1	Cs1^{11}	82.01(3)
Se2^{9}	Cs1	Cs1^{2}	57.450(12)	Hf1^{13}	Cu1	Cs1^{12}	82.01(3)
Se2^{10}	Cs1	Cs1^{4}	90.0	Hf1	Cu1	Cs1^{12}	116.37(5)
Se2^{5}	Cs1	Cs1^{4}	122.549(12)	Hf1^{14}	Cu1	Cs1^{11}	82.01(3)
Se2^{5}	Cs1	Cs1^{2}	57.450(12)	Hf1^{14}	Cu1	Cs1^{12}	116.37(5)
Se2^{10}	Cs1	Cs1^{2}	90.0	Hf1^{4}	Cu1	Cs1^{12}	82.01(3)
Se2^{5}	Cs1	Se2^{10}	83.03(2)	Cs1^{12}	Cu1	Cs1^{11}	58.43(4)
Se2^{6}	Cs1	Se2^{7}	69.81(4)	Se2	Cu1	Hf1^{13}	139.56(5)
Se2^{7}	Cs1	Se2^{10}	145.294(15)	Se2	Cu1	Hf1^{4}	54.457(17)
Se2^{6}	Cs1	Se2^{9}	103.52(4)	Se2^{15}	Cu1	Hf1^{14}	54.457(17)
Se2^{5}	Cs1	Se2^{9}	69.81(4)	Se2^{15}	Cu1	Hf1^{4}	139.56(5)
Se2^{6}	Cs1	Se2^{8}	145.294(15)	Se2	Cu1	Hf1	54.457(17)
Se2^{6}	Cs1	Se2^{10}	83.03(2)	Se2^{15}	Cu1	Hf1^{13}	54.457(17)
Se2^{5}	Cs1	Se2^{6}	65.10(2)	Se2	Cu1	Hf1^{14}	139.56(5)
Se2^{9}	Cs1	Se2^{10}	145.294(15)	Se2^{15}	Cu1	Hf1	139.56(5)
Se2^{5}	Cs1	Se2^{7}	103.52(4)	Se2	Cu1	Cs1^{12}	63.33(5)
Se2^{9}	Cs1	Se2^{8}	83.03(2)	Se2^{15}	Cu1	Cs1^{11}	63.33(5)
Se2^{9}	Cs1	Se2^{7}	65.10(2)	Se2^{15}	Cu1	Cs1^{12}	63.33(5)

Table S31. Bond Angles for $CsCuHfSe_3$.

Atom	Atom	Atom	Angle/°	Atom	Atom	Atom	Angle/°
$Se2^{7}$	Cs1	$Se2^{8}$	83.03(2)	Se2	Cu1	$Cs1^{11}$	63.33(5)
$Se2^{5}$	Cs1	$Se2^{8}$	145.294(15)	Se2	Cu1	$Se2^{15}$	118.10(12)
$Se2^{8}$	Cs1	$Se2^{10}$	111.05(4)	Se2	Cu1	$Se1^{2}$	108.20(2)
$Se2^{5}$	Cs1	$Cu1^{5}$	36.60(2)	$Se2^{15}$	Cu1	Se1	108.20(2)
$Se2^{10}$	Cs1	$Cu1^{6}$	119.608(16)	$Se2^{15}$	Cu1	$Se1^{2}$	108.20(2)
$Se2^{8}$	Cs1	$Cu1^{6}$	119.608(16)	Se2	Cu1	Se1	108.20(2)
$Se2^{8}$	Cs1	$Cu1^{5}$	119.608(16)	$Se1^{2}$	Cu1	$Hf1^{14}$	54.494(18)
$Se2^{7}$	Cs1	$Cu1^{5}$	73.88(3)	Se1	Cu1	$Hf1^{13}$	54.494(18)
$Se2^{9}$	Cs1	$Cu1^{6}$	73.88(3)	Se1	Cu1	$Hf1^{14}$	111.62(6)
$Se2^{10}$	Cs1	$Cu1^{5}$	119.608(16)	Se1	Cu1	$Hf1^{4}$	54.494(18)
$Se2^{7}$	Cs1	$Cu1^{6}$	36.60(2)	$Se1^{2}$	Cu1	$Hf1^{4}$	111.62(6)
$Se2^{9}$	Cs1	$Cu1^{5}$	36.60(2)	$Se1^{2}$	Cu1	$Hf1^{13}$	111.62(6)
$Se2^{6}$	Cs1	$Cu1^{6}$	36.60(2)	Se1	Cu1	Hf1	111.62(6)
$Se2^{5}$	Cs1	$Cu1^{6}$	73.88(3)	$Se1^{2}$	Cu1	Hf1	54.494(18)
$Se2^{6}$	Cs1	$Cu1^{5}$	73.88(3)	Se1	Cu1	$Cs1^{12}$	98.18(4)
$Se1^{4}$	Cs1	$Cs1^{2}$	123.216(17)	$Se1^{2}$	Cu1	$Cs1^{11}$	98.18(4)
Se1	Cs1	$Cs1^{4}$	123.216(17)	$Se1^{2}$	Cu1	$Cs1^{12}$	156.61(7)
Se1	Cs1	$Cs1^{2}$	56.785(17)	Se1	Cu1	$Cs1^{11}$	156.61(7)
$Se1^{4}$	Cs1	$Cs1^{4}$	56.785(17)	Se1	Cu1	$Se1^{2}$	105.21(11)

[1]2-X,1-Y,1-Z; [2]1+X,+Y,+Z; [3]1-X,1-Y,1-Z; [4]-1+X,+Y,+Z; [5]-1/2+X,1/2+Y,+Z; [6]-3/2+X,1/2+Y,+Z; [7]-3/2+X,1/2+Y,1/2-Z; [8]-X,1-Y,-1/2+Z; [9]-1/2+X,1/2+Y,1/2-Z; [10]-X,1-Y,1-Z; [11]3/2+X,-1/2+Y,+Z; [12]1/2+X,-1/2+Y,+Z; [13]1-X,1-Y,-1/2+Z; [14]2-X,1-Y,-1/2+Z; [15]+X,+Y,1/2-Z

$NaCuHfTe_3$ tables (S32-S36)

Table S32. Crystal data and structure refinement for $NaCuHfTe_3$.

Identification code	2465834
Empirical formula	$NaCuHfTe_3$
Formula weight	647.82
Temperature/K	173.00(10)
Crystal system	monoclinic
Space group	$P2_1/m$
a/Å	8.2105(10)
b/Å	4.0344(5)
c/Å	10.2118(11)
α/°	90
β/°	108.317(12)
γ/°	90
Volume/Å^3	321.12(7)

Z	2
ρ_{calc}g/cm^3	6.700
μ/mm^{-1}	138.334
F(000)	536.0
Crystal size/mm^3	0.065 × 0.022 × 0.021
Radiation	Cu Kα (λ = 1.54184)
2Θ range for data collection/°	9.122 to 151.806
Index ranges	-9 ≤ h ≤ 10, -4 ≤ k ≤ 5, -12 ≤ l ≤ 12
Reflections collected	2627
Independent reflections	705 [R_{int} = 0.0898, R_{sigma} = 0.0704]
Data/restraints/parameters	705/0/37
Goodness-of-fit on F^2	1.017
Final R indexes [I>=2σ (I)]	R_1 = 0.0693, wR_2 = 0.1804
Final R indexes [all data]	R_1 = 0.0780, wR_2 = 0.1899
Largest diff. peak/hole / e Å^{-3}	3.03/-2.83

Table S33. Fractional Atomic Coordinates (×10^4) and Equivalent Isotropic Displacement Parameters (Å^2×10^3) for $NaCuHfTe_3$. U_{eq} is defined as 1/3 of the trace of the orthogonalised U_{IJ} tensor.

Atom	x	y	z	U(eq)	Wyckoff site	Symmetry
Hf1	3021.1(17)	12500	8282.0(12)	29.9(5)	2e	m
Te1	5696(2)	7500	8658.4(16)	29.4(5)	2e	m
Te3	2580(2)	12500	5441.9(16)	30.8(6)	2e	m
Te2	664(2)	7500	8491.4(16)	30.8(6)	2e	m
Cu1	811(6)	7500	5967(4)	35.9(10)	2e	m
Na1	7350(20)	2500	7002(15)	56(4)	2e	m

Table S34. Anisotropic Displacement Parameters (Å^2×10^3) for $NaCuHfTe_3$. The Anisotropic displacement factor exponent takes the form: $-2\pi^2[h^2a^{*2}U_{11}+2hka^*b^*U_{12}+\ldots]$.

Atom	U_{11}	U_{22}	U_{33}	U_{23}	U_{13}	U_{12}
Hf1	36.1(8)	21.6(9)	28.4(7)	0	4.9(5)	0
Te1	34.6(10)	20.4(11)	29.4(9)	0	4.9(7)	0
Te3	37.1(10)	22.6(11)	28.0(9)	0	3.4(7)	0
Te2	34.9(9)	21.4(11)	33.1(9)	0	6.0(7)	0
Cu1	42(2)	25(2)	36(2)	0	5.0(18)	0
Na1	67(9)	43(9)	58(8)	0	18(7)	0

Table S35. Bond Lengths for $NaCuHfTe_3$.

Atom	Atom	Length/Å	Atom	Atom	Length/Å
Hf1	Te1[1]	2.9173(16)	Te3	Cu1	2.638(3)

Table S35. Bond Lengths for $NaCuHfTe_3$.

Atom	Atom	Length/Å	Atom	Atom	Length/Å
Hf1	Te1	2.9173(16)	Te3	Cu1^{3}	2.702(5)
Hf1	Te1^{2}	2.9670(19)	Te3	Na1^{4}	3.224(11)
Hf1	Te3	2.808(2)	Te3	Na1^{5}	3.224(11)
Hf1	Te2^{1}	2.8495(15)	Te2	Cu1	2.619(4)
Hf1	Te2	2.8495(15)	Te2	Na1^{6}	3.346(13)
Hf1	Cu1	3.203(3)	Te2	Na1^{7}	3.346(13)
Hf1	Cu1^{1}	3.203(3)	Cu1	Cu1^{8}	2.844(6)
Te1	Na1	3.196(12)	Cu1	Cu1^{3}	2.844(6)
Te1	Na1^{1}	3.196(12)	Na1	Na1^{1}	4.0344(5)
Te3	Cu1^{1}	2.637(3)	Na1	Na1^{9}	4.0344(5)

^{1}+X,1+Y,+Z; ^{2}1-X,2-Y,2-Z; ^{3}-X,2-Y,1-Z; ^{4}1-X,1-Y,1-Z; ^{5}1-X,2-Y,1-Z; ^{6}-1+X,+Y,+Z; ^{7}-1+X,1+Y,+Z; ^{8}-X,1-Y,1-Z; ^{9}+X,-1+Y,+Z

Table S36. Bond Angles for $NaCuHfTe_3$.

Atom	Atom	Atom	Angle/˚	Atom	Atom	Atom	Angle/˚
Te1^{1}	Hf1	Te1	87.49(6)	Cu1	Te2	Na1^{7}	80.5(3)
Te1^{1}	Hf1	Te1^{2}	81.83(5)	Cu1	Te2	Na1^{8}	80.5(3)
Te1	Hf1	Te1^{2}	81.83(5)	Na1^{7}	Te2	Na1^{8}	74.2(3)
Te1^{2}	Hf1	Cu1^{1}	135.13(6)	Hf1	Cu1	Hf1^{3}	78.08(10)
Te1^{1}	Hf1	Cu1^{1}	83.70(7)	Te3	Cu1	Hf1	56.48(7)
Te1^{2}	Hf1	Cu1	135.13(6)	Te3^{3}	Cu1	Hf1	114.28(15)
Te1	Hf1	Cu1	83.70(7)	Te3^{3}	Cu1	Hf1^{3}	56.48(7)
Te1^{1}	Hf1	Cu1	139.58(9)	Te3^{6}	Cu1	Hf1	130.01(11)
Te1	Hf1	Cu1^{1}	139.58(9)	Te3^{6}	Cu1	Hf1^{3}	130.01(11)
Te3	Hf1	Te1	89.05(6)	Te3	Cu1	Hf1^{3}	114.28(15)
Te3	Hf1	Te1^{1}	89.05(6)	Te3^{3}	Cu1	Te3	99.78(16)
Te3	Hf1	Te1^{2}	167.34(8)	Te3^{3}	Cu1	Te3^{6}	115.66(11)
Te3	Hf1	Te2	101.97(6)	Te3	Cu1	Te3^{6}	115.66(11)
Te3	Hf1	Te2^{1}	101.97(5)	Te3^{6}	Cu1	Cu1^{9}	56.72(15)
Te3	Hf1	Cu1^{1}	51.55(7)	Te3^{6}	Cu1	Cu1^{6}	56.72(15)
Te3	Hf1	Cu1	51.55(7)	Te3	Cu1	Cu1^{6}	58.94(10)
Te2^{1}	Hf1	Te1^{1}	90.09(4)	Te3	Cu1	Cu1^{9}	124.7(2)
Te2^{1}	Hf1	Te1^{2}	86.89(5)	Te3^{3}	Cu1	Cu1^{6}	124.7(2)
Te2^{1}	Hf1	Te1	168.68(7)	Te3^{3}	Cu1	Cu1^{9}	58.94(10)
Te2	Hf1	Te1^{2}	86.89(5)	Te2	Cu1	Hf1	57.58(8)
Te2	Hf1	Te1	90.09(4)	Te2	Cu1	Hf1^{3}	57.57(8)
Te2	Hf1	Te1^{1}	168.68(7)	Te2	Cu1	Te3	113.50(12)

Table S36. Bond Angles for $NaCuHfTe_3$.

Atom	Atom	Atom	Angle/°	Atom	Atom	Atom	Angle/°
Te2	Hf1	Te2^{1}	90.13(6)	Te2	Cu1	Te3^{3}	113.50(12)
Te2^{1}	Hf1	Cu1^{1}	50.87(8)	Te2	Cu1	Te3^{6}	99.53(16)
Te2	Hf1	Cu1^{1}	105.11(8)	Te2	Cu1	Cu1^{9}	121.81(18)
Te2^{1}	Hf1	Cu1	105.11(8)	Te2	Cu1	Cu1^{6}	121.81(18)
Te2	Hf1	Cu1	50.87(8)	Cu1^{9}	Cu1	Hf1^{3}	95.67(8)
Cu1	Hf1	Cu1^{1}	78.08(10)	Cu1^{9}	Cu1	Hf1	172.9(2)
Hf1	Te1	Hf1^{3}	87.49(6)	Cu1^{6}	Cu1	Hf1	95.67(8)
Hf1^{3}	Te1	Hf1^{2}	98.17(5)	Cu1^{6}	Cu1	Hf1^{3}	172.9(2)
Hf1	Te1	Hf1^{2}	98.17(5)	Cu1^{9}	Cu1	Cu1^{6}	90.4(2)
Hf1	Te1	Na1	142.6(3)	Te1^{3}	Na1	Te1	78.3(3)
Hf1^{2}	Te1	Na1	119.2(3)	Te1	Na1	Te3^{5}	156.8(6)
Hf1^{3}	Te1	Na1^{1}	142.6(3)	Te1^{3}	Na1	Te3^{4}	156.8(6)
Hf1^{2}	Te1	Na1^{1}	119.2(3)	Te1	Na1	Te3^{4}	97.40(14)
Hf1	Te1	Na1^{1}	85.5(2)	Te1^{3}	Na1	Te3^{5}	97.40(14)
Hf1^{3}	Te1	Na1	85.5(2)	Te1	Na1	Te2^{10}	124.1(5)
Na1	Te1	Na1^{1}	78.3(3)	Te1^{3}	Na1	Te2^{11}	124.1(5)
Hf1	Te3	Na1^{4}	140.52(18)	Te1	Na1	Te2^{11}	78.4(2)
Hf1	Te3	Na1^{5}	140.52(17)	Te1^{3}	Na1	Te2^{10}	78.4(2)
Cu1	Te3	Hf1	71.97(9)	Te1^{3}	Na1	Na1^{1}	129.14(17)
Cu1^{6}	Te3	Hf1	109.06(11)	Te1	Na1	Na1^{1}	50.86(17)
Cu1^{1}	Te3	Hf1	71.97(9)	Te1	Na1	Na1^{3}	129.14(17)
Cu1^{1}	Te3	Cu1^{6}	64.34(11)	Te1^{3}	Na1	Na1^{3}	50.86(17)
Cu1^{1}	Te3	Cu1	99.78(16)	Te3^{5}	Na1	Te3^{4}	77.5(3)
Cu1	Te3	Cu1^{6}	64.34(11)	Te3^{5}	Na1	Te2^{11}	121.3(5)
Cu1^{1}	Te3	Na1^{5}	141.4(3)	Te3^{4}	Na1	Te2^{10}	121.3(5)
Cu1	Te3	Na1^{5}	79.9(2)	Te3^{4}	Na1	Te2^{11}	76.4(2)
Cu1^{6}	Te3	Na1^{5}	81.6(3)	Te3^{5}	Na1	Te2^{10}	76.4(2)
Cu1	Te3	Na1^{4}	141.4(3)	Te3^{5}	Na1	Na1^{1}	128.74(16)
Cu1^{6}	Te3	Na1^{4}	81.6(3)	Te3^{4}	Na1	Na1^{3}	128.74(16)
Cu1^{1}	Te3	Na1^{4}	79.9(2)	Te3^{4}	Na1	Na1^{1}	51.26(16)
Na1^{5}	Te3	Na1^{4}	77.5(3)	Te3^{5}	Na1	Na1^{3}	51.26(16)
Hf1^{3}	Te2	Hf1	90.13(6)	Te2^{11}	Na1	Te2^{10}	74.2(3)
Hf1^{3}	Te2	Na1^{7}	90.8(2)	Te2^{10}	Na1	Na1^{1}	127.08(17)
Hf1	Te2	Na1^{7}	150.2(2)	Te2^{10}	Na1	Na1^{3}	52.92(17)
Hf1	Te2	Na1^{8}	90.8(2)	Te2^{11}	Na1	Na1^{1}	52.92(17)
Hf1^{3}	Te2	Na1^{8}	150.2(2)	Te2^{11}	Na1	Na1^{3}	127.08(17)
Cu1	Te2	Hf1^{3}	71.56(8)	Na1^{1}	Na1	Na1^{3}	180.0

Table S36. Bond Angles for $NaCuHfTe_3$.

Atom	Atom	Atom	Angle/˚	Atom	Atom	Atom	Angle/˚
Cu1	Te2	Hf1	71.56(8)				

^{1}+X,1+Y,+Z; ^{2}1-X,2-Y,2-Z; ^{3}+X,-1+Y,+Z; ^{4}1-X,2-Y,1-Z; ^{5}1-X,1-Y,1-Z; ^{6}-X,2-Y,1-Z; ^{7}-1+X,+Y,+Z; ^{8}-1+X,1+Y,+Z; ^{9}-X,1-Y,1-Z; ^{10}1+X,-1+Y,+Z; ^{11}1+X,+Y,+Z

$KCuHfTe_3$ (S37-S41)

Table S37. Crystal data and structure refinement for $KCuHfTe_3$.

Identification code	2465830
Empirical formula	$KCuHfTe_3$
Formula weight	663.93
Temperature/K	173.15
Crystal system	monoclinic
Space group	$P2_1/m$
a/Å	8.5914(3)
b/Å	4.1129(2)
c/Å	10.6995(5)
α/°	90
β/°	111.654(5)
γ/°	90
Volume/Å^3	351.39(3)
Z	2
ρ_{calc}g/cm^3	6.275
μ/mm^{-1}	131.119
F(000)	552.0
Crystal size/mm^3	0.25 × 0.04 × 0.03
Radiation	CuKα (λ = 1.54184)
2Θ range for data collection/°	8.892 to 149.606
Index ranges	-10 ≤ h ≤ 10, -4 ≤ k ≤ 4, -12 ≤ l ≤ 13
Reflections collected	1599
Independent reflections	765 [R_{int} = 0.0445, R_{sigma} = 0.0376]
Data/restraints/parameters	765/0/38
Goodness-of-fit on F^2	1.074
Final R indexes [I>=2σ (I)]	R_1 = 0.0474, wR_2 = 0.1259
Final R indexes [all data]	R_1 = 0.0481, wR_2 = 0.1271
Largest diff. peak/hole / e Å^{-3}	2.63/-3.02

Table S38. Fractional Atomic Coordinates (×10^4) and Equivalent Isotropic Displacement Parameters (Å^2×10^3) for $KCuHfTe_3$. U_{eq} is defined as 1/3 of the trace of the orthogonalised U_{IJ} tensor.

Atom	x	y	z	U(eq)	Wyckoff site	Symmetry
Hf1	6990.8(8)	7500	1685.3(6)	19.5(3)	2e	m
Te1	4430.9(10)	2500	1279.4(9)	18.3(4)	2e	m
Te2	9233.9(10)	2500	1540.2(9)	20.1(4)	2e	m
Te3	7543.3(11)	7500	4430.3(9)	20.5(4)	2e	m
Cu1	9179(3)	2500	3972(2)	26.5(6)	2e	m
K1	2730(4)	-2500	3027(4)	25.8(7)	2e	m

Table S39. Anisotropic Displacement Parameters (Å^2×10^3) for $KCuHfTe_3$. The Anisotropic displacement factor exponent takes the form: $-2\pi^2[h^2a^{*2}U_{11}+2hka^*b^*U_{12}+\ldots]$.

Atom	U_{11}	U_{22}	U_{33}	U_{23}	U_{13}	U_{12}
Hf1	24.8(5)	10.3(6)	23.5(5)	0	8.8(3)	0
Te1	22.8(5)	9.2(6)	23.0(5)	0	8.5(4)	0
Te2	23.5(5)	10.3(6)	26.9(5)	0	9.9(4)	0
Te3	26.1(5)	12.1(6)	23.6(5)	0	9.2(4)	0
Cu1	30.5(11)	19.8(14)	29.4(12)	0	11.4(10)	0
K1	32.0(14)	13.6(18)	36.3(16)	0	18.0(13)	0

Table S40. Bond Lengths for $KCuHfTe_3$.

Atom	Atom	Length/Å	Atom	Atom	Length/Å
Hf1	Te1^1	2.9240(7)	Te3	Cu1^1	2.6366(16)
Hf1	Te1	2.9240(7)	Te3	Cu1	2.6366(15)
Hf1	Te1^2	2.9487(10)	Te3	Cu1^5	2.702(2)
Hf1	Te2^1	2.8615(7)	Te3	K1^6	3.487(3)
Hf1	Te2	2.8615(7)	Te3	K1^7	3.487(3)
Hf1	Te3	2.7968(10)	Te3	K1^1	3.843(3)
Hf1	Cu1^1	3.2176(18)	Cu1	Cu1^5	2.962(3)
Hf1	Cu1	3.2176(18)	Cu1	Cu1^8	2.962(3)
Te1	K1^1	3.446(3)	K1	K1^1	4.1129(2)
Te1	K1	3.446(3)	K1	K1^9	4.11290(19)
Te2	Cu1	2.620(2)	K1	K1^7	5.011(7)
Te2	K1^3	3.501(3)	K1	K1^{10}	5.011(7)
Te2	K1^4	3.501(3)			

^{1}+X,1+Y,+Z; ^{2}1-X,1-Y,-Z; ^{3}1+X,1+Y,+Z; ^{4}1+X,+Y,+Z; ^{5}2-X,1-Y,1-Z; ^{6}1-X,1-Y,1-Z; ^{7}1-X,-Y,1-Z; ^{8}2-X,-Y,1-Z; ^{9}+X,-1+Y,+Z; ^{10}1-X,-1-Y,1-Z

Table S41. Bond Angles for $KCuHfTe_3$.

Atom	Atom	Atom	Angle/°	Atom	Atom	Atom	Angle/°
Te1^{1}	Hf1	Te1	89.39(3)	Te2	Cu1	Cu1^{8}	124.02(9)
Te1^{1}	Hf1	Te1^{2}	81.37(3)	Te3^{3}	Cu1	Hf1^{3}	56.02(3)
Te1	Hf1	Te1^{2}	81.37(3)	Te3	Cu1	Hf1^{3}	115.98(8)
Te1	Hf1	Cu1	81.94(3)	Te3	Cu1	Hf1	56.02(3)
Te1^{1}	Hf1	Cu1	139.36(5)	Te3^{3}	Cu1	Hf1	115.98(8)
Te1^{2}	Hf1	Cu1^{1}	135.38(4)	Te3^{8}	Cu1	Hf1^{3}	131.38(5)
Te1^{2}	Hf1	Cu1	135.38(4)	Te3^{8}	Cu1	Hf1	131.38(5)
Te1	Hf1	Cu1^{1}	139.35(5)	Te3^{3}	Cu1	Te3^{8}	112.63(6)
Te1^{1}	Hf1	Cu1^{1}	81.94(3)	Te3	Cu1	Te3^{8}	112.63(6)
Te2	Hf1	Te1	88.351(18)	Te3^{3}	Cu1	Te3	102.51(8)
Te2	Hf1	Te1^{2}	87.80(3)	Te3^{8}	Cu1	Cu1^{9}	55.26(7)
Te2^{1}	Hf1	Te1^{2}	87.80(3)	Te3^{3}	Cu1	Cu1^{8}	122.95(12)
Te2^{1}	Hf1	Te1^{1}	88.351(17)	Te3^{8}	Cu1	Cu1^{8}	55.26(7)
Te2	Hf1	Te1^{1}	169.14(4)	Te3^{3}	Cu1	Cu1^{9}	57.37(5)
Te2^{1}	Hf1	Te1	169.15(4)	Te3	Cu1	Cu1^{8}	57.37(5)
Te2	Hf1	Te2^{1}	91.89(3)	Te3	Cu1	Cu1^{9}	122.96(12)
Te2	Hf1	Cu1	50.64(4)	Cu1^{9}	Cu1	Hf1^{3}	96.06(4)
Te2^{1}	Hf1	Cu1^{1}	50.64(4)	Cu1^{9}	Cu1	Hf1	173.33(11)
Te2^{1}	Hf1	Cu1	106.52(4)	Cu1^{8}	Cu1	Hf1	96.06(4)
Te2	Hf1	Cu1^{1}	106.52(4)	Cu1^{8}	Cu1	Hf1^{3}	173.33(11)
Te3	Hf1	Te1^{1}	89.02(3)	Cu1^{8}	Cu1	Cu1^{9}	87.96(12)
Te3	Hf1	Te1	89.02(3)	Te1	K1	Te1^{3}	73.27(6)
Te3	Hf1	Te1^{2}	166.43(4)	Te1	K1	Te2^{10}	82.38(5)
Te3	Hf1	Te2	101.55(3)	Te1^{3}	K1	Te2^{11}	82.38(5)
Te3	Hf1	Te2^{1}	101.55(3)	Te1	K1	Te2^{11}	124.64(11)
Te3	Hf1	Cu1^{1}	51.42(4)	Te1^{3}	K1	Te2^{10}	124.64(11)
Te3	Hf1	Cu1	51.42(4)	Te1	K1	Te3^{7}	103.62(3)
Cu1^{1}	Hf1	Cu1	79.45(5)	Te1^{3}	K1	Te3^{7}	159.96(12)
Hf1	Te1	Hf1^{3}	89.38(3)	Te1	K1	Te3^{3}	66.52(6)
Hf1^{3}	Te1	Hf1^{2}	98.63(3)	Te1^{3}	K1	Te3^{6}	103.63(3)
Hf1	Te1	Hf1^{2}	98.63(3)	Te1	K1	Te3^{6}	159.96(12)
Hf1	Te1	K1^{1}	86.90(4)	Te1^{3}	K1	Te3^{3}	66.52(6)
Hf1^{2}	Te1	K1^{1}	119.57(6)	Te1^{3}	K1	K1^{12}	81.85(6)
Hf1^{3}	Te1	K1	86.90(4)	Te1^{3}	K1	K1^{3}	53.36(3)
Hf1^{2}	Te1	K1	119.57(6)	Te1^{3}	K1	K1^{1}	126.64(3)
Hf1	Te1	K1	141.75(7)	Te1	K1	K1^{12}	110.37(11)

Table S41. Bond Angles for $KCuHfTe_3$.

Atom	Atom	Atom	Angle/°	Atom	Atom	Atom	Angle/°
Hf1^{3}	Te1	K1^{1}	141.75(7)	Te1	K1	K1^{6}	81.85(6)
K1^{1}	Te1	K1	73.27(6)	Te1	K1	K1^{1}	53.36(3)
Hf1	Te2	Hf1^{3}	91.89(3)	Te1^{3}	K1	K1^{6}	110.37(11)
Hf1	Te2	K1^{4}	151.60(6)	Te1	K1	K1^{3}	126.64(3)
Hf1	Te2	K1^{5}	92.02(4)	Te2^{11}	K1	Te2^{10}	71.95(7)
Hf1^{3}	Te2	K1^{4}	92.02(4)	Te2^{10}	K1	Te3^{3}	142.80(4)
Hf1^{3}	Te2	K1^{5}	151.59(6)	Te2^{11}	K1	Te3^{3}	142.80(4)
Cu1	Te2	Hf1	71.74(4)	Te2^{10}	K1	K1^{1}	54.03(4)
Cu1	Te2	Hf1^{3}	71.74(4)	Te2^{11}	K1	K1^{3}	54.03(4)
Cu1	Te2	K1^{5}	82.86(7)	Te2^{10}	K1	K1^{6}	114.35(4)
Cu1	Te2	K1^{4}	82.86(7)	Te2^{11}	K1	K1^{1}	125.97(4)
K1^{5}	Te2	K1^{4}	71.95(7)	Te2^{11}	K1	K1^{6}	153.43(12)
Hf1	Te3	K1^{6}	141.73(4)	Te2^{11}	K1	K1^{12}	114.35(4)
Hf1	Te3	K1^{7}	141.73(4)	Te2^{10}	K1	K1^{12}	153.43(12)
Hf1	Te3	K1^{1}	81.29(6)	Te2^{10}	K1	K1^{3}	125.97(4)
Cu1^{1}	Te3	Hf1	72.56(5)	Te3^{7}	K1	Te2^{10}	73.41(5)
Cu1	Te3	Hf1	72.56(5)	Te3^{6}	K1	Te2^{10}	114.04(9)
Cu1^{8}	Te3	Hf1	113.44(6)	Te3^{7}	K1	Te2^{11}	114.04(9)
Cu1^{1}	Te3	Cu1	102.51(8)	Te3^{6}	K1	Te2^{11}	73.41(5)
Cu1	Te3	Cu1^{8}	67.37(6)	Te3^{7}	K1	Te3^{6}	72.28(7)
Cu1^{1}	Te3	Cu1^{8}	67.37(6)	Te3^{6}	K1	Te3^{3}	93.89(8)
Cu1^{1}	Te3	K1^{6}	143.05(8)	Te3^{7}	K1	Te3^{3}	93.89(8)
Cu1^{8}	Te3	K1^{1}	165.27(8)	Te3^{7}	K1	K1^{6}	49.92(5)
Cu1	Te3	K1^{6}	83.06(6)	Te3^{3}	K1	K1^{12}	43.96(6)
Cu1	Te3	K1^{1}	119.84(5)	Te3^{6}	K1	K1^{3}	53.86(3)
Cu1^{8}	Te3	K1^{7}	82.01(7)	Te3^{3}	K1	K1^{6}	43.96(6)
Cu1^{8}	Te3	K1^{6}	82.01(7)	Te3^{3}	K1	K1^{3}	90.000(1)
Cu1	Te3	K1^{7}	143.05(8)	Te3^{7}	K1	K1^{12}	80.81(9)
Cu1^{1}	Te3	K1^{1}	119.84(5)	Te3^{6}	K1	K1^{6}	80.81(9)
Cu1^{1}	Te3	K1^{7}	83.06(6)	Te3^{6}	K1	K1^{12}	49.92(5)
K1^{6}	Te3	K1^{1}	86.11(8)	Te3^{7}	K1	K1^{3}	126.14(3)
K1^{7}	Te3	K1^{6}	72.28(7)	Te3^{7}	K1	K1^{1}	53.86(3)
K1^{7}	Te3	K1^{1}	86.11(8)	Te3^{3}	K1	K1^{1}	90.0
Hf1^{3}	Cu1	Hf1	79.45(5)	Te3^{6}	K1	K1^{1}	126.14(3)
Te2	Cu1	Hf1^{3}	57.62(4)	K1^{1}	K1	K1^{6}	65.77(3)
Te2	Cu1	Hf1	57.62(4)	K1^{3}	K1	K1^{6}	114.23(3)
Te2	Cu1	Te3	113.02(6)	K1^{3}	K1	K1^{12}	65.77(3)

Table S41. Bond Angles for $KCuHfTe_3$.

Atom	Atom	Atom	Angle/˚	Atom	Atom	Atom	Angle/˚
Te2	Cu1	$Te3^{8}$	103.40(8)	$K1^{6}$	K1	$K1^{12}$	48.46(7)
Te2	Cu1	$Te3^{3}$	113.02(6)	$K1^{1}$	K1	$K1^{12}$	114.23(3)
Te2	Cu1	$Cu1^{9}$	124.02(9)	$K1^{1}$	K1	$K1^{3}$	180.0

^{1}+X,1+Y,+Z; ^{2}1-X,1-Y,-Z; ^{3}+X,-1+Y,+Z; ^{4}1+X,+Y,+Z; ^{5}1+X,1+Y,+Z; ^{6}1-X,-Y,1-Z; ^{7}1-X,1-Y,1-Z; ^{8}2-X,1-Y,1-Z; ^{9}2-X,-Y,1-Z; ^{10}-1+X,+Y,+Z; ^{11}-1+X,-1+Y,+Z; ^{12}1-X,-1-Y,1-Z

$RbCuHfTe_3$ (S42-S46)

Table S42. Crystal data and structure refinement for $RbCuHfTe_3$.

Identification code	2465831
Empirical formula	$RbCuHfTe_3$
Formula weight	710.30
Temperature/K	173.00(10)
Crystal system	monoclinic
Space group	$P2_1/m$
a/Å	9.4034(7)
b/Å	4.1366(3)
c/Å	10.3921(8)
α/°	90
β/°	113.068(9)
γ/°	90
Volume/Å^3	371.91(5)
Z	2
ρ_{calc}g/cm^3	6.343
μ/mm^{-1}	126.789
F(000)	588.0
Crystal size/mm^3	0.06 × 0.03 × 0.02
Radiation	Cu Kα (λ = 1.54184)
2Θ range for data collection/°	9.25 to 150.894
Index ranges	-11 ≤ h ≤ 10, -4 ≤ k ≤ 4, -12 ≤ l ≤ 12
Reflections collected	1757
Independent reflections	805 [R_{int} = 0.0499, R_{sigma} = 0.0561]
Data/restraints/parameters	805/0/37
Goodness-of-fit on F^2	1.050
Final R indexes [I>=2σ (I)]	R_1 = 0.0586, wR_2 = 0.1518
Final R indexes [all data]	R_1 = 0.0644, wR_2 = 0.1562
Largest diff. peak/hole / e Å^{-3}	2.63/-2.54

Table S43. Fractional Atomic Coordinates (×10^4) and Equivalent Isotropic Displacement Parameters (Å^2×10^3) for $RbCuHfTe_3$. U_{eq} is defined as 1/3 of the trace of the orthogonalised U_{IJ} tensor.

Atom	x	y	z	U(eq)	Wyckoff site	Symmetry
Hf1	6952.6(12)	12500	1662.2(10)	23.3(4)	2e	m
Te1	4668.8(14)	7500	1417.6(13)	22.8(4)	2e	m
Te2	8932.1(15)	7500	1383.1(14)	24.2(4)	2e	m
Te3	7760.4(15)	12500	4539.7(13)	25.4(4)	2e	m
Rb1	2388(3)	2500	2668(2)	31.8(6)	2e	m
Cu1	9165(4)	7500	3984(4)	30.8(8)	2e	m

Table S44. Anisotropic Displacement Parameters (Å^2×10^3) for $RbCuHfTe_3$. The Anisotropic displacement factor exponent takes the form: $-2\pi^2[h^2a^{*2}U_{11}+2hka^*b^*U_{12}+\ldots]$.

Atom	U_{11}	U_{22}	U_{33}	U_{23}	U_{13}	U_{12}
Hf1	26.7(6)	17.4(7)	21.3(6)	0	4.5(4)	0
Te1	25.0(7)	17.0(9)	22.1(7)	0	4.7(5)	0
Te2	26.2(7)	18.3(9)	24.9(7)	0	6.3(5)	0
Te3	31.0(8)	18.8(9)	22.5(7)	0	6.4(5)	0
Rb1	37.6(12)	23.8(14)	34.8(12)	0	15.0(9)	0
Cu1	37.8(18)	22(2)	28.4(16)	0	8.6(13)	0

Table S45. Bond Lengths for $RbCuHfTe_3$.

Atom	Atom	Length/Å	Atom	Atom	Length/Å
Hf1	Te1	2.9201(11)	Te2	Rb1^5	3.636(2)
Hf1	Te1^1	2.9201(11)	Te2	Cu1	2.625(4)
Hf1	Te1^2	2.9570(16)	Te3	Rb1^6	3.611(2)
Hf1	Te2	2.8722(12)	Te3	Rb1^7	3.611(2)
Hf1	Te2^1	2.8722(12)	Te3	Cu1^8	2.690(4)
Hf1	Te3	2.7819(16)	Te3	Cu1^1	2.637(2)
Hf1	Cu1^1	3.239(3)	Te3	Cu1	2.637(2)
Hf1	Cu1	3.239(3)	Rb1	Rb1^9	4.1366(3)
Te1	Rb1^1	3.570(2)	Rb1	Rb1^1	4.1366(3)
Te1	Rb1	3.570(2)	Cu1	Cu1^8	2.933(5)
Te2	Rb1^3	3.636(2)	Cu1	Cu1^{10}	2.933(5)
Te2	Rb1^4	3.894(3)			

^{1}+X,1+Y,+Z; ^{2}1-X,2-Y,-Z; ^{3}1+X,+Y,+Z; ^{4}1-X,1-Y,-Z; ^{5}1+X,1+Y,+Z; ^{6}1-X,1-Y,1-Z; ^{7}1-X,2-Y,1-Z; ^{8}2-X,2-Y,1-Z; ^{9}+X,-1+Y,+Z; ^{10}2-X,1-Y,1-Z

Table S46. Bond Angles for $RbCuHfTe_3$.

Atom	Atom	Atom	Angle/°	Atom	Atom	Atom	Angle/°
Te1	Hf1	Te1^{1}	90.19(4)	Cu1	Te3	Cu1^{1}	103.33(13)
Te1	Hf1	Te1^{2}	81.72(4)	Cu1	Te3	Cu1^{9}	66.82(9)
Te1^{1}	Hf1	Te1^{2}	81.72(4)	Te1^{3}	Rb1	Te1	70.80(5)
Te1	Hf1	Cu1	81.27(5)	Te1	Rb1	Te2^{10}	96.59(4)
Te1^{2}	Hf1	Cu1^{1}	135.60(5)	Te1^{3}	Rb1	Te2^{11}	96.59(4)
Te1^{1}	Hf1	Cu1^{1}	81.27(5)	Te1	Rb1	Te2^{11}	140.70(8)
Te1^{1}	Hf1	Cu1	138.83(8)	Te1^{3}	Rb1	Te2^{10}	140.70(8)
Te1	Hf1	Cu1^{1}	138.83(8)	Te1^{3}	Rb1	Te2^{5}	65.75(4)
Te1^{2}	Hf1	Cu1	135.60(5)	Te1	Rb1	Te2^{5}	65.75(4)
Te2	Hf1	Te1	87.98(3)	Te1^{3}	Rb1	Te3^{8}	100.28(3)
Te2	Hf1	Te1^{1}	170.02(5)	Te1^{3}	Rb1	Te3^{7}	147.37(8)
Te2^{1}	Hf1	Te1^{2}	88.31(4)	Te1	Rb1	Te3^{8}	147.37(8)
Te2	Hf1	Te1^{2}	88.31(4)	Te1	Rb1	Te3^{7}	100.28(3)
Te2^{1}	Hf1	Te1	170.02(5)	Te1^{3}	Rb1	Rb1^{3}	54.60(2)
Te2^{1}	Hf1	Te1^{1}	87.98(3)	Te1	Rb1	Rb1^{1}	54.60(2)
Te2^{1}	Hf1	Te2	92.13(5)	Te1^{3}	Rb1	Rb1^{1}	125.40(2)
Te2^{1}	Hf1	Cu1	106.42(6)	Te1	Rb1	Rb1^{3}	125.40(2)
Te2	Hf1	Cu1	50.44(6)	Te2^{11}	Rb1	Te2^{5}	75.09(5)
Te2^{1}	Hf1	Cu1^{1}	50.44(6)	Te2^{11}	Rb1	Te2^{10}	69.33(5)
Te2	Hf1	Cu1^{1}	106.42(6)	Te2^{10}	Rb1	Te2^{5}	75.09(5)
Te3	Hf1	Te1^{2}	166.23(6)	Te2^{11}	Rb1	Rb1^{3}	55.33(2)
Te3	Hf1	Te1	88.58(4)	Te2^{5}	Rb1	Rb1^{1}	90.0
Te3	Hf1	Te1^{1}	88.58(4)	Te2^{5}	Rb1	Rb1^{3}	90.0
Te3	Hf1	Te2	101.17(4)	Te2^{10}	Rb1	Rb1^{1}	55.33(2)
Te3	Hf1	Te2^{1}	101.17(4)	Te2^{10}	Rb1	Rb1^{3}	124.67(2)
Te3	Hf1	Cu1	51.25(6)	Te2^{11}	Rb1	Rb1^{1}	124.67(2)
Te3	Hf1	Cu1^{1}	51.25(6)	Te3^{7}	Rb1	Te2^{5}	140.58(4)
Cu1^{1}	Hf1	Cu1	79.36(8)	Te3^{8}	Rb1	Te2^{11}	70.05(4)
Hf1^{3}	Te1	Hf1	90.19(4)	Te3^{8}	Rb1	Te2^{5}	140.58(4)
Hf1^{3}	Te1	Hf1^{2}	98.28(4)	Te3^{7}	Rb1	Te2^{10}	70.05(4)
Hf1	Te1	Hf1^{2}	98.28(4)	Te3^{7}	Rb1	Te2^{11}	108.08(6)
Hf1	Te1	Rb1	154.79(6)	Te3^{8}	Rb1	Te2^{10}	108.08(6)
Hf1^{3}	Te1	Rb1^{1}	154.79(6)	Te3^{7}	Rb1	Te3^{8}	69.89(5)
Hf1^{2}	Te1	Rb1	105.39(5)	Te3^{8}	Rb1	Rb1^{3}	55.05(2)
Hf1^{2}	Te1	Rb1^{1}	105.39(5)	Te3^{7}	Rb1	Rb1^{1}	55.05(2)
Hf1	Te1	Rb1^{1}	94.83(3)	Te3^{7}	Rb1	Rb1^{3}	124.95(2)

Table S46. Bond Angles for $RbCuHfTe_3$.

Atom	Atom	Atom	Angle/°	Atom	Atom	Atom	Angle/°
Hf1[3]	Te1	Rb1	94.83(3)	Te3[8]	Rb1	Rb1[1]	124.95(2)
Rb1[1]	Te1	Rb1	70.80(5)	Rb1[1]	Rb1	Rb1[3]	180.0
Hf1	Te2	Hf1[3]	92.13(5)	Hf1[3]	Cu1	Hf1	79.36(8)
Hf1	Te2	Rb1[4]	94.28(3)	Te2	Cu1	Hf1	57.51(6)
Hf1	Te2	Rb1[5]	99.46(4)	Te2	Cu1	Hf1[3]	57.51(7)
Hf1	Te2	Rb1[6]	153.36(6)	Te2	Cu1	Te3	112.25(10)
Hf1[3]	Te2	Rb1[6]	94.28(3)	Te2	Cu1	Te3[3]	112.25(10)
Hf1[3]	Te2	Rb1[4]	153.36(6)	Te2	Cu1	Te3[9]	102.97(13)
Hf1[3]	Te2	Rb1[5]	99.46(4)	Te2	Cu1	Cu1[9]	123.11(15)
Rb1[4]	Te2	Rb1[6]	69.33(5)	Te2	Cu1	Cu1[12]	123.11(15)
Rb1[6]	Te2	Rb1[5]	104.91(5)	Te3	Cu1	Hf1[3]	115.69(12)
Rb1[4]	Te2	Rb1[5]	104.91(5)	Te3	Cu1	Hf1	55.37(5)
Cu1	Te2	Hf1	72.05(6)	Te3[9]	Cu1	Hf1	131.12(8)
Cu1	Te2	Hf1[3]	72.05(6)	Te3[9]	Cu1	Hf1[3]	131.12(8)
Cu1	Te2	Rb1[4]	85.42(8)	Te3[3]	Cu1	Hf1	115.69(12)
Cu1	Te2	Rb1[6]	85.42(8)	Te3[3]	Cu1	Hf1[3]	55.37(5)
Cu1	Te2	Rb1[5]	167.34(10)	Te3	Cu1	Te3[9]	113.19(9)
Hf1	Te3	Rb1[7]	141.60(3)	Te3	Cu1	Te3[3]	103.33(13)
Hf1	Te3	Rb1[8]	141.60(4)	Te3[3]	Cu1	Te3[9]	113.19(9)
Rb1[7]	Te3	Rb1[8]	69.89(5)	Te3[3]	Cu1	Cu1[12]	57.47(8)
Cu1[9]	Te3	Hf1	113.12(9)	Te3	Cu1	Cu1[12]	124.64(19)
Cu1	Te3	Hf1	73.37(8)	Te3[3]	Cu1	Cu1[9]	124.64(19)
Cu1[1]	Te3	Hf1	73.37(8)	Te3[9]	Cu1	Cu1[9]	55.72(11)
Cu1[9]	Te3	Rb1[8]	85.02(8)	Te3	Cu1	Cu1[9]	57.47(8)
Cu1	Te3	Rb1[7]	143.68(10)	Te3[9]	Cu1	Cu1[12]	55.72(11)
Cu1	Te3	Rb1[8]	84.67(7)	Cu1[12]	Cu1	Hf1[3]	95.31(6)
Cu1[1]	Te3	Rb1[8]	143.68(10)	Cu1[9]	Cu1	Hf1[3]	173.14(17)
Cu1[1]	Te3	Rb1[7]	84.67(7)	Cu1[9]	Cu1	Hf1	95.31(6)
Cu1[9]	Te3	Rb1[7]	85.02(8)	Cu1[12]	Cu1	Hf1	173.14(17)
Cu1[1]	Te3	Cu1[9]	66.82(9)	Cu1[9]	Cu1	Cu1[12]	89.67(19)

[1]+X,1+Y,+Z; [2]1-X,2-Y,-Z; [3]+X,-1+Y,+Z; [4]1+X,1+Y,+Z; [5]1-X,1-Y,-Z; [6]1+X,+Y,+Z; [7]1-X,2-Y,1-Z; [8]1-X,1-Y,1-Z; [9]2-X,2-Y,1-Z; [10]-1+X,+Y,+Z; [11]-1+X,-1+Y,+Z; [12]2-X,1-Y,1-Z

$CsCuHfTe_3$ (S47-S52)

Table S47. Crystal data and structure refinement for $CsCuHfTe_3$.

Identification code	2465835
Empirical formula	$CsCuHfTe_3$
Formula weight	757.74
Temperature/K	173.00(10)
Crystal system	monoclinic
Space group	$P2_1/m$
a/Å	10.8326(8)
b/Å	3.9617(3)
c/Å	10.8989(8)
α/°	90
β/°	119.665(10)
γ/°	90
Volume/Å^3	406.43(6)
Z	2
ρ_{calc}g/cm^3	6.192
μ/mm^{-1}	143.184
F(000)	624.0
Crystal size/mm^3	0.03 × 0.02 × 0.01
Radiation	Cu Kα (λ = 1.54184)
2Θ range for data collection/°	9.396 to 150.79
Index ranges	-13 ≤ h ≤ 13, -4 ≤ k ≤ 4, -13 ≤ l ≤ 10
Reflections collected	2305
Independent reflections	892 [R_{int} = 0.0711, R_{sigma} = 0.0814]
Data/restraints/parameters	892/0/43
Goodness-of-fit on F^2	1.006
Final R indexes [I>=2σ (I)]	R_1 = 0.0577, wR_2 = 0.1415
Final R indexes [all data]	R_1 = 0.0757, wR_2 = 0.1508
Largest diff. peak/hole / e Å^{-3}	3.25/-3.65

Table S48. Fractional Atomic Coordinates (×10^4) and Equivalent Isotropic Displacement Parameters (Å^2×10^3) for $CsCuHfTe_3$. U_{eq} is defined as 1/3 of the trace of the orthogonalised U_{IJ} tensor.

Atom	x	y	z	U(eq)	Wyckoff site	Symmetry
Hf1	6869.2(12)	7500	1636.4(13)	19.6(4)	2e	m

Table S48. Fractional Atomic Coordinates (×10^4) and Equivalent Isotropic Displacement Parameters (Å^2×10^3) for $CsCuHfTe_3$. U_{eq} is defined as 1/3 of the trace of the orthogonalised U_{IJ} tensor.

Atom	x	y	z	U(eq)	Wyckoff site	Symmetry
Te1	4837.6(15)	2500	1474.5(16)	18.2(4)	2e	m
Te2	8635.5(16)	2500	1397.4(17)	20.5(4)	2e	m
Te3	8013.7(16)	7500	4596.2(17)	20.6(4)	2e	m
Cu1	9187(4)	2500	4002(4)	22.7(9)	2e	m
Cs1	2166(5)	7500	2404(5)	78.3(16)	2e	m
Cs2	4766(10)	7500	4820(12)	103(4)	2e	m

Table S49. Anisotropic Displacement Parameters (Å^2×10^3) for $CsCuHfTe_3$. The Anisotropic displacement factor exponent takes the form: $-2\pi^2[h^2a^{*2}U_{11}+2hka^*b^*U_{12}+\ldots]$.

Atom	U_{11}	U_{22}	U_{33}	U_{23}	U_{13}	U_{12}
Hf1	17.3(6)	19.1(8)	15.3(7)	0	2.7(5)	0
Te1	15.4(7)	19.2(10)	13.5(9)	0	2.1(6)	0
Te2	18.6(8)	20.4(10)	18.0(9)	0	5.6(7)	0
Te3	17.3(7)	23.4(10)	14.3(9)	0	2.6(6)	0
Cu1	18.9(17)	24(2)	17(2)	0	2.5(15)	0
Cs1	63(2)	125(5)	57(3)	0	38(2)	0
Cs2	59(5)	174(12)	84(7)	0	42(5)	0

Table S50. Bond Lengths for $CsCuHfTe_3$.

Atom	Atom	Length/Å	Atom	Atom	Length/Å
Hf1	Te1	2.9007(14)	Te3	Cu1^1	2.602(3)
Hf1	Te1^1	2.9007(14)	Te3	Cu1	2.602(3)
Hf1	Te1^2	2.951(2)	Te3	Cs1^8	3.909(4)
Hf1	Te2	2.8542(14)	Te3	Cs1^4	3.909(4)
Hf1	Te2^1	2.8542(14)	Te3	Cs2	3.645(9)
Hf1	Te3	2.826(2)	Te3	Cs2^4	3.910(8)
Hf1	Cu1^1	3.235(3)	Te3	Cs2^8	3.910(8)
Hf1	Cu1	3.235(3)	Cu1	Cu1^9	2.823(5)
Te1	Cs1^3	4.028(4)	Cu1	Cu1^7	2.823(5)
Te1	Cs1	4.028(4)	Cs1	Cs1^3	3.9617(3)
Te1	Cs2^4	3.844(11)	Cs1	Cs1^1	3.9617(3)
Te1	Cs2	4.184(9)	Cs1	Cs2^8	3.764(10)
Te1	Cs2^3	4.184(9)	Cs1	Cs2^4	3.764(10)
Te2	Cu1	2.596(4)	Cs1	Cs2	2.743(12)
Te2	Cs1^2	3.789(5)	Cs2	Cs2^4	2.034(5)

Table S50. Bond Lengths for $CsCuHfTe_3$.

Atom	Atom	Length/Å	Atom	Atom	Length/Å
Te2	Cs1^{5}	3.950(4)	Cs2	Cs2^{3}	3.9617(3)
Te2	Cs1^{6}	3.950(4)	Cs2	Cs2^{1}	3.9617(3)
Te3	Cu1^{7}	2.636(4)	Cs2	Cs2^{8}	2.034(5)

^{1}+X,1+Y,+Z; ^{2}1-X,1-Y,-Z; ^{3}+X,-1+Y,+Z; ^{4}1-X,1-Y,1-Z; ^{5}1+X,+Y,+Z; ^{6}1+X,-1+Y,+Z; ^{7}2-X,1-Y,1-Z; ^{8}1-X,2-Y,1-Z; ^{9}2-X,-Y,1-Z

Table S51. Bond Angles for $CsCuHfTe_3$.

Atom	Atom	Atom	Angle/˚	Atom	Atom	Atom	Angle/˚
Te1	Hf1	Te1^{1}	86.14(5)	Cu1^{8}	Cu1	Cu1^{9}	89.1(2)
Te1	Hf1	Te1^{2}	84.55(5)	Te1^{1}	Cs1	Te1	58.91(6)
Te1^{1}	Hf1	Te1^{2}	84.55(5)	Te2^{2}	Cs1	Te1^{1}	61.93(6)
Te1	Hf1	Cu1^{1}	137.12(8)	Te2^{10}	Cs1	Te1	153.39(13)
Te1^{1}	Hf1	Cu1^{1}	84.06(6)	Te2^{11}	Cs1	Te1^{1}	153.39(13)
Te1^{2}	Hf1	Cu1^{1}	135.51(6)	Te2^{11}	Cs1	Te1	113.63(5)
Te1^{2}	Hf1	Cu1	135.51(6)	Te2^{2}	Cs1	Te1	61.93(6)
Te1	Hf1	Cu1	84.06(6)	Te2^{10}	Cs1	Te1^{1}	113.63(5)
Te1^{1}	Hf1	Cu1	137.12(8)	Te2^{2}	Cs1	Te2^{10}	91.71(9)
Te2^{1}	Hf1	Te1^{1}	92.48(3)	Te2^{2}	Cs1	Te2^{11}	91.71(9)
Te2	Hf1	Te1	92.48(3)	Te2^{11}	Cs1	Te2^{10}	60.20(7)
Te2	Hf1	Te1^{2}	87.83(5)	Te2^{2}	Cs1	Te3^{7}	146.63(6)
Te2^{1}	Hf1	Te1	172.36(6)	Te2^{2}	Cs1	Te3^{4}	146.63(6)
Te2^{1}	Hf1	Te1^{2}	87.83(5)	Te2^{2}	Cs1	Cs1^{1}	90.0
Te2	Hf1	Te1^{1}	172.36(6)	Te2^{11}	Cs1	Cs1^{1}	120.10(3)
Te2^{1}	Hf1	Te2	87.90(5)	Te2^{11}	Cs1	Cs1^{3}	59.90(3)
Te2^{1}	Hf1	Cu1^{1}	49.98(7)	Te2^{10}	Cs1	Cs1^{1}	59.90(3)
Te2^{1}	Hf1	Cu1	101.94(7)	Te2^{10}	Cs1	Cs1^{3}	120.10(3)
Te2	Hf1	Cu1	49.98(7)	Te2^{2}	Cs1	Cs1^{3}	90.0
Te2	Hf1	Cu1^{1}	101.94(7)	Te3^{7}	Cs1	Te1	143.03(13)
Te3	Hf1	Te1^{2}	169.43(6)	Te3^{7}	Cs1	Te1^{1}	107.49(6)
Te3	Hf1	Te1	87.73(5)	Te3^{4}	Cs1	Te1^{1}	143.03(13)
Te3	Hf1	Te1^{1}	87.73(5)	Te3^{4}	Cs1	Te1	107.49(6)
Te3	Hf1	Te2^{1}	99.73(5)	Te3^{7}	Cs1	Te2^{11}	92.53(9)
Te3	Hf1	Te2	99.73(5)	Te3^{4}	Cs1	Te2^{10}	92.53(9)
Te3	Hf1	Cu1	50.30(7)	Te3^{7}	Cs1	Te2^{10}	62.35(6)
Te3	Hf1	Cu1^{1}	50.30(7)	Te3^{4}	Cs1	Te2^{11}	62.35(6)
Cu1^{1}	Hf1	Cu1	75.51(9)	Te3^{7}	Cs1	Te3^{4}	60.89(7)
Hf1^{3}	Te1	Hf1^{2}	95.45(5)	Te3^{7}	Cs1	Cs1^{3}	120.45(3)

Table S51. Bond Angles for $CsCuHfTe_3$.

Atom	Atom	Atom	Angle/°	Atom	Atom	Atom	Angle/°
Hf1^3	Te1	Hf1	86.14(5)	Te3^4	Cs1	Cs1^3	59.55(3)
Hf1	Te1	Hf1^2	95.45(5)	Te3^7	Cs1	Cs1^1	59.55(3)
Hf1^3	Te1	Cs1^3	105.47(4)	Te3^4	Cs1	Cs1^1	120.45(3)
Hf1^2	Te1	Cs1	99.75(8)	Cs1^3	Cs1	Te1	60.54(3)
Hf1^2	Te1	Cs1^3	99.75(8)	Cs1^1	Cs1	Te1	119.46(3)
Hf1^3	Te1	Cs1	159.78(7)	Cs1^3	Cs1	Te1^1	119.46(3)
Hf1	Te1	Cs1	105.47(4)	Cs1^1	Cs1	Te1^1	60.54(3)
Hf1	Te1	Cs1^3	159.78(7)	Cs1^1	Cs1	Cs1^3	180.0
Hf1^3	Te1	Cs2^4	104.43(11)	Cs2^7	Cs1	Te1^1	59.00(16)
Hf1^3	Te1	Cs2^3	87.96(10)	Cs2^4	Cs1	Te1	59.00(16)
Hf1	Te1	Cs2	87.96(10)	Cs2	Cs1	Te1^1	73.6(2)
Hf1^2	Te1	Cs2	136.88(13)	Cs2^4	Cs1	Te1^1	90.14(15)
Hf1^3	Te1	Cs2	127.67(13)	Cs2	Cs1	Te1	73.6(2)
Hf1^2	Te1	Cs2^3	136.88(13)	Cs2^7	Cs1	Te1	90.14(15)
Hf1^2	Te1	Cs2^4	152.59(15)	Cs2^4	Cs1	Te2^2	120.81(17)
Hf1	Te1	Cs2^3	127.67(13)	Cs2^7	Cs1	Te2^{10}	107.92(11)
Hf1	Te1	Cs2^4	104.43(11)	Cs2^4	Cs1	Te2^{10}	146.67(18)
Cs1	Te1	Cs1^3	58.91(6)	Cs2	Cs1	Te2^{11}	131.27(19)
Cs1^3	Te1	Cs2	71.83(10)	Cs2^4	Cs1	Te2^{11}	107.92(11)
Cs1^3	Te1	Cs2^3	38.96(15)	Cs2	Cs1	Te2^{10}	131.27(19)
Cs1	Te1	Cs2	38.96(15)	Cs2^7	Cs1	Te2^{11}	146.67(18)
Cs1	Te1	Cs2^3	71.83(10)	Cs2	Cs1	Te2^2	128.4(2)
Cs2^4	Te1	Cs1	57.07(13)	Cs2^7	Cs1	Te2^2	120.81(17)
Cs2^4	Te1	Cs1^3	57.07(13)	Cs2	Cs1	Te3^4	69.5(2)
Cs2^4	Te1	Cs2^3	28.96(10)	Cs2^4	Cs1	Te3^7	89.09(16)
Cs2	Te1	Cs2^3	56.52(14)	Cs2^7	Cs1	Te3^4	89.09(16)
Cs2^4	Te1	Cs2	28.96(10)	Cs2^7	Cs1	Te3^7	56.69(15)
Hf1	Te2	Hf1^3	87.90(5)	Cs2	Cs1	Te3^7	69.5(2)
Hf1	Te2	Cs1^5	157.43(7)	Cs2^4	Cs1	Te3^4	56.69(15)
Hf1^3	Te2	Cs1^2	107.37(6)	Cs2	Cs1	Cs1^3	90.0
Hf1^3	Te2	Cs1^6	157.43(7)	Cs2^4	Cs1	Cs1^1	121.75(9)
Hf1	Te2	Cs1^2	107.37(6)	Cs2^7	Cs1	Cs1^1	58.25(9)
Hf1	Te2	Cs1^6	103.14(4)	Cs2^7	Cs1	Cs1^3	121.75(9)
Hf1^3	Te2	Cs1^5	103.14(4)	Cs2	Cs1	Cs1^1	90.000(1)
Cu1	Te2	Hf1^3	72.65(7)	Cs2^4	Cs1	Cs1^3	58.25(9)
Cu1	Te2	Hf1	72.65(7)	Cs2^7	Cs1	Cs2^4	63.50(18)
Cu1	Te2	Cs1^2	179.96(12)	Cs2	Cs1	Cs2^7	31.78(9)

Table S51. Bond Angles for $CsCuHfTe_3$.

Atom	Atom	Atom	Angle/°	Atom	Atom	Atom	Angle/°
Cu1	Te2	Cs1^{5}	91.68(10)	Cs2	Cs1	Cs2^{4}	31.78(9)
Cu1	Te2	Cs1^{6}	91.68(10)	Te1^{4}	Cs2	Te1^{1}	151.04(10)
Cs1^{2}	Te2	Cs1^{5}	88.29(9)	Te1^{4}	Cs2	Te3^{4}	61.56(15)
Cs1^{2}	Te2	Cs1^{6}	88.29(9)	Te1^{4}	Cs2	Te3^{7}	61.56(15)
Cs1^{6}	Te2	Cs1^{5}	60.20(7)	Te1^{4}	Cs2	Cs2^{3}	90.0
Hf1	Te3	Cs1^{4}	141.21(6)	Te1^{4}	Cs2	Cs2^{1}	90.0
Hf1	Te3	Cs1^{7}	141.21(6)	Te3^{7}	Cs2	Te1^{1}	104.47(15)
Hf1	Te3	Cs2^{4}	104.34(17)	Te3	Cs2	Te1^{1}	60.45(14)
Hf1	Te3	Cs2^{7}	104.34(17)	Te3^{4}	Cs2	Te1^{1}	136.9(3)
Hf1	Te3	Cs2	100.59(18)	Te3	Cs2	Te1^{4}	117.4(3)
Cu1^{8}	Te3	Hf1	113.00(11)	Te3	Cs2	Te3^{7}	149.02(9)
Cu1^{1}	Te3	Hf1	73.04(9)	Te3^{4}	Cs2	Te3^{7}	60.87(14)
Cu1	Te3	Hf1	73.04(9)	Te3	Cs2	Te3^{4}	149.02(9)
Cu1^{1}	Te3	Cu1	99.14(14)	Te3	Cs2	Cs1^{4}	63.66(16)
Cu1^{1}	Te3	Cu1^{8}	65.22(10)	Te3	Cs2	Cs1^{7}	63.66(16)
Cu1	Te3	Cu1^{8}	65.22(11)	Te3^{4}	Cs2	Cs2^{1}	120.44(7)
Cu1^{1}	Te3	Cs1^{7}	93.16(8)	Te3^{7}	Cs2	Cs2^{1}	59.56(7)
Cu1	Te3	Cs1^{4}	93.16(8)	Te3	Cs2	Cs2^{3}	90.0
Cu1^{1}	Te3	Cs1^{4}	145.74(11)	Te3^{4}	Cs2	Cs2^{3}	59.56(7)
Cu1	Te3	Cs1^{7}	145.74(11)	Te3^{7}	Cs2	Cs2^{3}	120.44(7)
Cu1^{8}	Te3	Cs1^{7}	91.98(11)	Te3	Cs2	Cs2^{1}	90.000(1)
Cu1^{8}	Te3	Cs1^{4}	91.98(11)	Cs1^{7}	Cs2	Te1^{4}	63.92(18)
Cu1^{1}	Te3	Cs2^{4}	159.42(11)	Cs1	Cs2	Te1^{1}	67.4(2)
Cu1	Te3	Cs2	128.53(8)	Cs1	Cs2	Te1^{4}	122.4(3)
Cu1^{1}	Te3	Cs2	128.53(8)	Cs1^{4}	Cs2	Te1^{1}	124.0(2)
Cu1^{1}	Te3	Cs2^{7}	99.50(10)	Cs1^{7}	Cs2	Te1^{1}	93.52(14)
Cu1^{8}	Te3	Cs2^{4}	131.56(16)	Cs1^{4}	Cs2	Te1^{4}	63.92(18)
Cu1^{8}	Te3	Cs2^{7}	131.56(16)	Cs1^{7}	Cs2	Te3^{7}	92.71(16)
Cu1	Te3	Cs2^{7}	159.42(11)	Cs1	Cs2	Te3^{4}	69.44(19)
Cu1	Te3	Cs2^{4}	99.50(10)	Cs1^{7}	Cs2	Te3^{4}	125.5(3)
Cu1^{8}	Te3	Cs2	146.41(19)	Cs1	Cs2	Te3^{7}	69.44(19)
Cs1^{7}	Te3	Cs1^{4}	60.89(7)	Cs1^{4}	Cs2	Te3^{4}	92.71(16)
Cs1^{4}	Te3	Cs2^{7}	76.08(13)	Cs1^{4}	Cs2	Te3^{7}	125.5(3)
Cs1^{7}	Te3	Cs2^{7}	41.07(17)	Cs1	Cs2	Te3	120.1(4)
Cs1^{4}	Te3	Cs2^{4}	41.07(17)	Cs1^{7}	Cs2	Cs1^{4}	63.50(18)
Cs1^{7}	Te3	Cs2^{4}	76.08(13)	Cs1	Cs2	Cs1^{4}	148.22(10)
Cs2	Te3	Cs1^{7}	59.65(16)	Cs1	Cs2	Cs1^{7}	148.22(10)

Table S51. Bond Angles for $CsCuHfTe_3$.

Atom	Atom	Atom	Angle/°	Atom	Atom	Atom	Angle/°
Cs2	Te3	Cs1^{4}	59.65(16)	Cs1^{4}	Cs2	Cs2^{1}	121.75(9)
Cs2^{4}	Te3	Cs2^{7}	60.87(14)	Cs1	Cs2	Cs2^{3}	90.000(2)
Cs2	Te3	Cs2^{4}	30.98(9)	Cs1^{7}	Cs2	Cs2^{3}	121.75(9)
Cs2	Te3	Cs2^{7}	30.98(9)	Cs1^{4}	Cs2	Cs2^{3}	58.25(9)
Hf1^{3}	Cu1	Hf1	75.51(9)	Cs1	Cs2	Cs2^{1}	90.000(1)
Te2	Cu1	Hf1^{3}	57.37(7)	Cs1^{7}	Cs2	Cs2^{1}	58.25(9)
Te2	Cu1	Hf1	57.37(7)	Cs2^{7}	Cs2	Te1^{4}	84.8(6)
Te2	Cu1	Te3^{3}	113.32(11)	Cs2^{3}	Cs2	Te1^{1}	118.26(7)
Te2	Cu1	Te3	113.32(11)	Cs2^{4}	Cs2	Te1^{4}	84.8(6)
Te2	Cu1	Te3^{8}	102.11(14)	Cs2^{1}	Cs2	Te1^{1}	61.74(7)
Te2	Cu1	Cu1^{8}	124.12(16)	Cs2^{4}	Cs2	Te1^{1}	121.2(6)
Te2	Cu1	Cu1^{9}	124.12(16)	Cs2^{7}	Cs2	Te1^{1}	66.2(5)
Te3^{3}	Cu1	Hf1^{3}	56.66(6)	Cs2^{4}	Cs2	Te3	81.7(5)
Te3^{3}	Cu1	Hf1	112.49(13)	Cs2^{7}	Cs2	Te3	81.7(5)
Te3	Cu1	Hf1^{3}	112.49(13)	Cs2^{4}	Cs2	Te3^{7}	126.9(6)
Te3^{8}	Cu1	Hf1	132.72(9)	Cs2^{7}	Cs2	Te3^{4}	126.9(6)
Te3	Cu1	Hf1	56.66(6)	Cs2^{7}	Cs2	Te3^{7}	67.3(4)
Te3^{8}	Cu1	Hf1^{3}	132.72(9)	Cs2^{4}	Cs2	Te3^{4}	67.3(4)
Te3^{3}	Cu1	Te3^{8}	114.78(10)	Cs2^{7}	Cs2	Cs1	103.0(6)
Te3^{3}	Cu1	Te3	99.14(14)	Cs2^{4}	Cs2	Cs1^{4}	45.2(5)
Te3	Cu1	Te3^{8}	114.78(11)	Cs2^{4}	Cs2	Cs1^{7}	108.7(7)
Te3^{8}	Cu1	Cu1^{8}	56.82(13)	Cs2^{4}	Cs2	Cs1	103.0(6)
Te3^{8}	Cu1	Cu1^{9}	56.82(13)	Cs2^{7}	Cs2	Cs1^{7}	45.2(5)
Te3^{3}	Cu1	Cu1^{9}	57.96(9)	Cs2^{7}	Cs2	Cs1^{4}	108.7(7)
Te3^{3}	Cu1	Cu1^{8}	122.5(2)	Cs2^{4}	Cs2	Cs2^{1}	166.8(6)
Te3	Cu1	Cu1^{9}	122.5(2)	Cs2^{7}	Cs2	Cs2^{1}	13.2(6)
Te3	Cu1	Cu1^{8}	57.96(9)	Cs2^{1}	Cs2	Cs2^{3}	180.0
Cu1^{9}	Cu1	Hf1^{3}	97.28(7)	Cs2^{7}	Cs2	Cs2^{4}	153.7(11)
Cu1^{8}	Cu1	Hf1	97.28(7)	Cs2^{7}	Cs2	Cs2^{3}	166.8(6)
Cu1^{8}	Cu1	Hf1^{3}	170.45(19)	Cs2^{4}	Cs2	Cs2^{3}	13.2(6)
Cu1^{9}	Cu1	Hf1	170.45(19)				

^{1}+X,1+Y,+Z; ^{2}1-X,1-Y,-Z; ^{3}+X,-1+Y,+Z; ^{4}1-X,1-Y,1-Z; ^{5}1+X,-1+Y,+Z; ^{6}1+X,+Y,+Z; ^{7}1-X,2-Y,1-Z; ^{8}2-X,1-Y,1-Z; ^{9}2-X,-Y,1-Z; ^{10}-1+X,1+Y,+Z; ^{11}-1+X,+Y,+Z

Table S52. Atomic Occupancy for $CsCuHfTe_3$.

Atom	Occupancy	Atom	Occupancy	Atom	Occupancy
Cs1	0.6667	Cs2	0.3333		
